# Endosomal phosphatidylinositol 3‐phosphate controls synaptic vesicle cycling and neurotransmission

**DOI:** 10.15252/embj.2021109352

**Published:** 2022-03-22

**Authors:** Guan‐Ting Liu, Gaga Kochlamazashvili, Dmytro Puchkov, Rainer Müller, Carsten Schultz, Albert I Mackintosh, Dennis Vollweiter, Volker Haucke, Tolga Soykan

**Affiliations:** ^1^ Leibniz‐Forschungsinstitut für Molekulare Pharmakologie (FMP) Berlin Germany; ^2^ European Molecular Biology Laboratory (EMBL) Cell Biology and Biophysics Unit Heidelberg Germany; ^3^ Department of Chemical Physiology & Biochemistry Oregon Health & Science University (OHSU) Portland OR USA; ^4^ Faculty of Biology, Chemistry, Pharmacy Freie Universität Berlin Berlin Germany; ^5^ NeuroCure Cluster of Excellence Charité Universitätsmedizin Berlin Berlin Germany

**Keywords:** endocytosis, endosomes, neurotransmission, phosphatidylinositol 3‐phosphate, synaptic vesicle, Membranes & Trafficking, Neuroscience

## Abstract

Neural circuit function requires mechanisms for controlling neurotransmitter release and the activity of neuronal networks, including modulation by synaptic contacts, synaptic plasticity, and homeostatic scaling. However, how neurons intrinsically monitor and feedback control presynaptic neurotransmitter release and synaptic vesicle (SV) recycling to restrict neuronal network activity remains poorly understood at the molecular level. Here, we investigated the reciprocal interplay between neuronal endosomes, organelles of central importance for the function of synapses, and synaptic activity. We show that elevated neuronal activity represses the synthesis of endosomal lipid phosphatidylinositol 3‐phosphate [PI(3)P] by the lipid kinase VPS34. Neuronal activity in turn is regulated by endosomal PI(3)P, the depletion of which reduces neurotransmission as a consequence of perturbed SV endocytosis. We find that this mechanism involves Calpain 2‐mediated hyperactivation of Cdk5 downstream of receptor‐ and activity‐dependent calcium influx. Our results unravel an unexpected function for PI(3)P‐containing neuronal endosomes in the control of presynaptic vesicle cycling and neurotransmission, which may explain the involvement of the PI(3)P‐producing VPS34 kinase in neurological disease and neurodegeneration.

## Graphical abstract

Enhanced neuronal activity lowers endosomal PI(3)P levels to perturb synaptic vesicle endocytosis and neuronal activity via Calpain 2‐mediated Cdk5 activation.

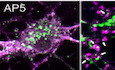

## Introduction

Neurotransmission is mediated by the stochastic calcium‐triggered fusion of presynaptic vesicles with the plasma membrane to release neurotransmitters (Sudhof & Rothman, 2009), which bind to and activate postsynaptic receptors, for example, ionotropic glutamate receptors. Neurotransmission is fine‐tuned in a synapse‐specific manner by short‐ and long‐term synaptic plasticity (Katz & Shatz, 1996; Abbott & Regehr, 2004), while the robustness of synaptic transmission is ensured by homeostatic adaptations such as presynaptic homeostatic plasticity that stabilize excitatory neurotransmission in response to alterations in postsynaptic function (Davis & Muller, 2015). For example, perturbation of postsynaptic excitability in *Drosophila* neuromuscular junctions (Davis & Goodman, 1998) is counteracted by a homeostatic facilitation of presynaptic neurotransmitter release. In addition to these pathways, the proper functioning of neuronal networks also requires mechanisms that limit excitatory neurotransmission. On the circuit level, glutamate‐induced postsynaptic depolarization is thought to be counteracted mainly by inhibitory synaptic inputs (Katz & Shatz, 1996; Abbott & Regehr, 2004; Marder & Goaillard, 2006; Hu *et al*, 2014). Consistently, impairment of inhibitory synapse function is associated with hyperexcitation and epileptiform activity in animal models and in humans (Nelson & Valakh, 2015). In parallel to circuit level organization, neurons may intrinsically monitor and adjust their excitability and neurotransmission via mechanisms that mediate crosstalk between synaptic activity and intracellular signaling. How such cell‐intrinsic control of neurotransmission is achieved at the molecular‐mechanistic level remains poorly understood, but could possibly involve alterations in the exo‐endocytosis of SVs, that is, the cell biological processes that underlie chemical neurotransmission.

SV cycling is based on the action potential (AP)‐triggered calcium‐driven exocytic fusion of SVs at active zone release sites (Jahn & Fasshauer, 2012; Sudhof, 2013). Exocytic SV fusion is followed by endocytosis of SV membranes and the endocytic reformation of functional SVs (Saheki & De Camilli, 2012; Rizzoli, 2014; Chanaday *et al*, 2019) from internal, possibly endosome‐like organelles via clathrin‐ and adaptor‐mediated budding (Cheung & Cousin, 2012; Kononenko *et al*, 2014; Watanabe *et al*, 2014). The speed and efficacy of SV endocytosis is facilitated by the calcium‐dependent phosphatase calcineurin (Saheki & De Camilli, 2012; Cheung & Cousin, 2013; Rizzoli, 2014; Wu *et al*, 2014; Soykan *et al*, 2016) and repressed by the synaptic protein kinase Cdk5 (Tan *et al*, 2003; Ferguson *et al*, 2007; Armbruster *et al*, 2013), a master regulatory switch for presynaptic neurotransmission (Kim & Ryan, 2010, 2013). In most cell types, endosomes are marked by high levels of phosphatidylinositol 3‐phosphate [PI(3)P], a signaling lipid that is crucial for endosome function (Di Paolo & De Camilli, 2006; Balla, 2013; Raiborg *et al*, 2013). Hence, endosomal PI(3)P may conceivably regulate SV cycling (see e.g., (Geumann *et al*, 2010; Uytterhoeven *et al*, 2011; Rizzoli, 2014; Jahne *et al*, 2015)) and, thereby, impinge on neuronal network activity, a hypothesis that we have tested in this study.

Here, we unravel a mechanism for the control of presynaptic function that involves a reciprocal interplay between synaptic activity and endosomal PI(3)P levels. We demonstrate that PI(3)P synthesis via VPS34 controls neurotransmission and SV cycling via a feedback‐regulated mechanism that determines the activity status of Calpain 2 and cyclin‐dependent kinase 5 (Cdk5), an enzyme crucial for tuning presynaptic neurotransmitter release and SV recycling (Tan *et al*, 2003; Kim & Ryan, 2010, 2013; Shah & Lahiri, 2014), to prevent hyperexcitation and epileptiform neuronal activity. Our data have implications for our understanding of neuronal network function and help to explain the role of VPS34 in neurological and neurodegenerative disorders.

## Results

### Neuronal activity represses endosomal PI(3)P synthesis mediated by the lipid kinase VPS34

**Figure 1:**
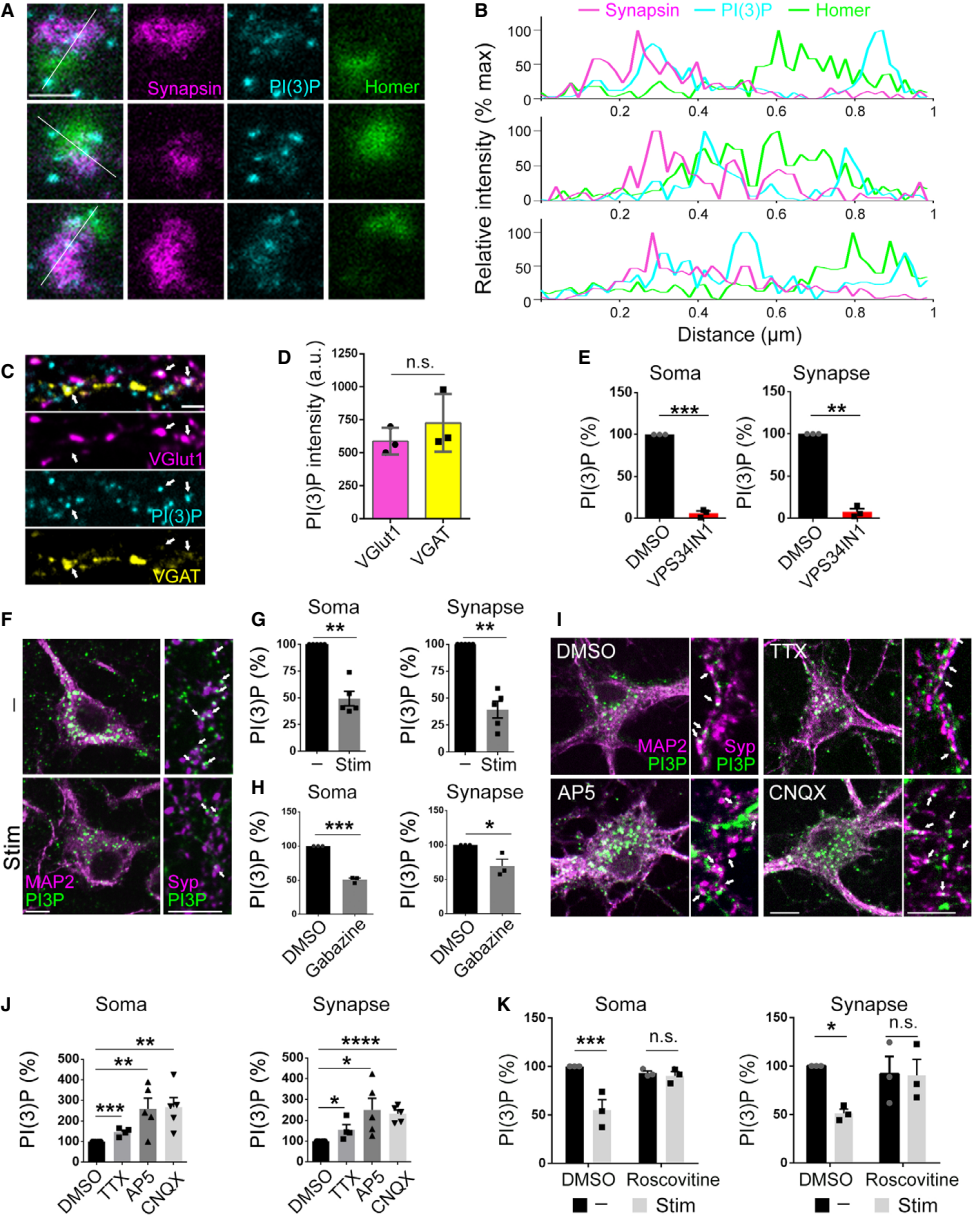
The abundance of somatic and synaptic PI(3)P‐positive endosomes is regulated by neuronal activity via Cdk5 Representative 3‐color STED images of synapses of cultured hippocampal neurons fixed and stained for Synapsin1/2 (presynaptic marker), PI(3)P, and Homer 1 (postsynaptic marker). Scale bar, 0.5 µm. PI(3)P‐positive structures appear in both pre‐ and postsynaptic compartments.Line scan fluorescence intensity profiles of single synapses from the images in A, normalized to maximum value for each channel.Representative 3‐color confocal images of cultured hippocampal neurons fixed and stained for VGlut1, VGAT, and PI(3)P. Arrows indicate PI(3)P puncta in VGlut1^+^ or VGAT^+^ presynaptic terminals. Scale bar, 10 µm.PI(3)P intensity in VGlut1^+^ and VGAT^+^ presynaptic terminals. PI(3)P is equally abundant in excitatory and inhibitory presynaptic terminals. *N* = 3 independent experiments (≥ 15 images per condition); Student’s *t*‐test.Relative PI(3)P levels in the MAP2^+^ (Soma) and Syp^+^ (Synapse) areas in DMSO and VPS34IN1‐treated neurons. *N* = 3 independent experiments (≥ 35 images per condition); Student’s *t*‐test.Cultured hippocampal neurons were field‐stimulated with four trains of 200 APs (40 Hz, 5 s; 90 s gap between each train) (Stim) or left unstimulated (−), fixed and stained for MAP2, Synaptophysin (Syp), and PI(3)P. Arrows indicate PI(3)P puncta in synapses. Scale bar, 10 µm. PI(3)P puncta that do not overlap with neuronal soma and synapses belong to astrocytes in culture and are disregarded from the quantification.Relative intensity of PI(3)P in neuronal somata (MAP2^+^) or synapses (Syp^+^) from the images in F. *N* = 5 independent experiments; (≥ 40 images per condition); Student’s *t*‐test.Relative intensity of PI(3)P in neuronal somata (MAP2^+^) or synapses (Syp^+^) of cultured hippocampal neurons treated with DMSO or Gabazine (10 µM) overnight. *N* = 3 independent experiments (≥ 65 images per condition); Student’s *t*‐test.Cultured hippocampal neurons treated with AP5 (20 µM), CNQX (10 µM) or TTX (0.2 µM) overnight, fixed and stained for MAP2, Syp and PI(3)P. Arrows indicate PI(3)P puncta in synapses. Scale bar, 10 µm.Relative intensity of PI(3)P in neuronal somata (MAP2^+^) or synapses (Syp^+^) from the images in I. *N* ≥ 4 independent experiments (≥ 60 images per condition); Student’s *t*‐test.Relative intensity of PI(3)P in neuronal somata (MAP2^+^) or synapses (Syp^+^) of cultured hippocampal neurons were treated with Roscovitine (10 µM) for 1 h and field‐stimulated with four trains of 200 APs (40 Hz, 5 s; 90 s gap between each train) (Stim) or left unstimulated (−). *N* = 3 independent experiments (≥ 40 images per condition); Two‐way ANOVA; Fisher’s Least Significant Difference Test. Data information: Data presented as mean ± SEM in all panels; n.s. not significant; *P < 0.05; **P < 0.01; ***P < 0.001; ****P < 0.0001; p, t, q, df values are provided in the Source Data Statistics file. Source data are available online for this figure.

Endosomes and endosome‐like organelles have been proposed to play important roles in synaptic function including the recycling of SV membranes, presynaptic protein sorting and quality control, and the exo‐endocytic trafficking of postsynaptic receptors during synaptic plasticity (Park *et al*, 2004; Uytterhoeven *et al*, 2011; van der Sluijs & Hoogenraad, 2011; Saheki & De Camilli, 2012; Rizzoli, 2014; Watanabe *et al*, 2014; Wu *et al*, 2014; Chanaday *et al*, 2019). Surprisingly little is known about the distribution and dynamics of neuronal endosomes marked by PI(3)P, a lipid of crucial importance for endosome and lysosome function in non‐neuronal cells (Simonsen *et al*, 1998; Di Paolo & De Camilli, 2006; Balla, 2013; Raiborg *et al*, 2013) and in neurons (Morel *et al*, 2013; Miranda *et al*, 2018). To close this knowledge gap, we first monitored the nanoscale distribution of PI(3)P in cultured hippocampal neurons by multicolor time‐gated stimulated emission depletion microscopy (gSTED) using the recombinant 2xFYVE domain of Hrs (Gaullier *et al*, 1998; Balla, 2013) (see also (Ketel *et al*, 2016) for specificity) as a specific probe. PI(3)P‐containing endosomes were detected throughout neuronal somata and neurites including pre‐ and postsynaptic compartments immunopositive for the SV‐associated protein Synapsin and postsynaptic Homer, respectively (Fig 1A and B). Further analysis by confocal microscopy imaging revealed similar levels of PI(3)P at excitatory synapses marked by the vesicular glutamate transporter 1 (vGLUT1) and at inhibitory synapses identified by the vesicular GABA transporter vGAT (Fig 1C and D). These data show that PI(3)P‐containing endosomes are present at both excitatory and inhibitory synapses, consistent with their presumed roles in synapse physiology and neurotransmission.

**Figure EV1:**
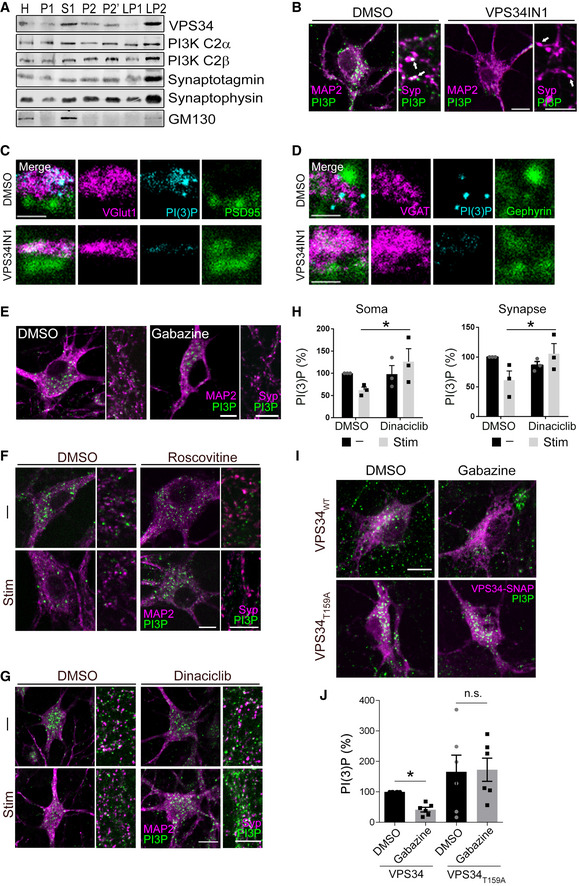
PI(3)P is localized at neuronal soma and synapses and endosomal PI(3)P levels are regulated by neuronal activity ASubcellular fractionation of mouse brains followed by western blot reveals the presence of Class II and Class III PI3Ks in the synaptic vesicle enriched LP2 fraction.BRepresentative 2‐color confocal images of cultured hippocampal neurons treated with DMSO (0.1%) or VPS34IN1 (10 µM) for 1 h, fixed and stained for MAP2, synaptophysin (Syp), and PI(3)P. Arrows indicate presynaptic terminals that contain (DMSO) or lack (VPS34IN1) PI(3)P. Scale bar, 10 µm.C, DRepresentative 3‐color STED images of synapses of cultured hippocampal neurons treated with DMSO (0.1%) or VPS34IN1 (10 µM) for 1 h, fixed and stained for PI(3)P and VGlut1‐PSD95 pair (markers for excitatory synapses) in C and VGAT‐Gephyrin pair (markers for inhibitory synapses) in D. Scale bar, 0.5 µm. Both excitatory and inhibitory synaptic terminals contain PI(3)P‐positive structures, which disappear upon VPS34 inhibition.ECultured hippocampal neurons treated with Gabazine (10 µM) overnight, fixed and stained for MAP2, Syp and PI(3)P. Scale bar, 10 µm.FCultured hippocampal neurons were treated with Roscovitine (10 µM) for 1 h and field‐stimulated with four trains of 200 APs (40 Hz, 5 s; 90 s gap between each train) (Stim) or left unstimulated (−), fixed and stained for MAP2, Syp, and PI(3)P. Scale bar, 10 µm.GCultured hippocampal neurons were treated with Dinaciclib (10 µM) for 1 h and field‐stimulated with four trains of 200 APs (40 Hz, 5 s; 90 s gap between each train) (Stim) or left unstimulated (−), fixed and stained for MAP2, Syp, and PI(3)P. Scale bar, 10 µm.HRelative intensity of PI(3)P in neuronal somata (MAP2^+^) or synapses (Syp^+^) of cultured hippocampal neurons were treated with Dinaciclib (10 µM) for 1 h and field‐stimulated with four trains of 200 APs (40 Hz, 5 s; 90 s gap between each train) (Stim) or left unstimulated (−). *N* = 3 independent experiments (≥ 15 images per condition); Mean ± SEM; *P < 0.05; Two‐way ANOVA.ICultured hippocampal neurons transfected with VPS34‐SNAP or VPS34_T159A_‐SNAP at DIV7, treated with Gabazine (10 µM) overnight at DIV13, treated 1 h with SNAP‐tag ligand JF646 to label VPS34‐SNAP at DIV14 and subsequently fixed and stained for PI(3)P. Scale bar, 10 µm.JRelative intensity of PI(3)P in neuronal somata of cultured hippocampal neurons expressing VPS34‐SNAP or VPS34_T159A_‐SNAP, treated with DMSO or Gabazine (10 µM) overnight. *N* = 6 independent experiments (≥ 30 images per condition); Mean ± SEM; *P < 0.05; Two‐way ANOVA. Source data are available online for this figure.

To obtain further insights into the dynamics and function of PI(3)P‐containing endosomes at synapses, we analyzed the mechanism of neuronal PI(3)P synthesis. Synthesis of PI(3)P in most mammalian cells and tissues is mediated predominantly by the endosomally localized class III PI 3‐kinase VPS34, an essential factor for neuronal survival to prevent neurodegeneration (Zhou *et al*, 2010; Morel *et al*, 2013), and, to a minor extent, by the class II PI 3‐kinases PI3K‐C2α and PI3K‐C2β (Di Paolo & De Camilli, 2006; Balla, 2013). We confirmed the expression of VPS34, PI3K‐C2α, and PI3K‐C2β in brain and found these enzymes to be enriched in crude SV fractions prepared from lysed presynaptic nerve terminals (Fig EV1A). Selective inhibition of VPS34 by the well‐established specific small molecule inhibitor VPS34IN1 (Bago *et al*, 2014; Ketel *et al*, 2016) effectively reduced somatic and synaptic PI(3)P to nearly undetectable levels (Figs 1E and EV1B). A similar efficacy of VPS34IN1‐mediated PI(3)P depletion was observed at excitatory and inhibitory presynaptic sites (Fig EV1C and D). Hence, the endosomal class III PI 3‐kinase VPS34 is the major enzyme that produces PI(3)P at excitatory and inhibitory synapses in hippocampal neurons.

Given these data and the alleged functions of neuronal or synaptic endosomes (Park *et al*, 2004; Uytterhoeven *et al*, 2011; van der Sluijs & Hoogenraad, 2011; Saheki & De Camilli, 2012; Rizzoli, 2014; Watanabe *et al*, 2014; Wu *et al*, 2014; Chanaday *et al*, 2019), we hypothesized that endosomal PI(3)P may impinge on or be controlled by neuronal network activity. Sustained stimulation of hippocampal neurons with repetitive trains of APs (200 APs, 40 Hz, 5 s) resulted in the depletion of PI(3)P from endosomal puncta present in neuronal somata and at presynaptic nerve terminals marked by the SV protein Synaptophysin (Fig 1F and G). In striking contrast, we confirmed the previously observed stimulation‐induced elevation of presynaptic phosphatidylinositol 4,5‐bisphosphate [PI(4,5)P_2_] levels at active synapses (not shown; (Micheva *et al*, 2001)). Elevation of neuronal network activity by disinhibition in the presence of the γ‐amino‐butyric acid (GABA) type A (GABA_A_) receptor antagonist Gabazine (Zhao *et al*, 2011; Jeans *et al*, 2017), led to a similar reduction in somatic and synaptic PI(3)P levels (Figs 1H and EV1E). Conversely, blockade of endogenous network activity by the N‐methyl‐D‐aspartate (NMDA) receptor antagonist AP5, the sodium channel blocker tetrodotoxin (TTX), or the α‐amino‐3‐hydroxy‐5‐methyl‐4‐isoxazolepropionic acid (AMPA) receptor inhibitor CNQX caused a profound elevation of somatic and synaptic PI(3)P levels (Fig 1I and J). Endosomal PI(3)P levels are, thus, inversely correlated with and, possibly, controlled by neuronal activity. In non‐neuronal cells, cyclin‐dependent kinases have been shown to repress VPS34‐mediated PI(3)P synthesis by phosphorylating VPS34 at Thr159 (Furuya *et al*, 2010). We therefore reasoned that the observed repression of PI(3)P synthesis by neuronal activity might be mediated by Cdk5, a major negative regulator of neurotransmission (Kim & Ryan, 2010, 2013) and SV endocytosis (Tan *et al*, 2003; Ferguson *et al*, 2007; Armbruster *et al*, 2013). Consistently, we found that pharmacological inhibition of Cdk5 activity by Roscovitine or Dinaciclib occludes the activity‐induced repression of VPS34‐mediated PI(3)P synthesis in neuronal somata and at synapses (Figs 1K and EV1F–H). Furthermore, overexpression of a nonphosphorylatable mutant of VPS34 (T159A) led to elevated PI(3)P levels in neuronal somata and occluded Gabazine‐induced downregulation of PI(3)P synthesis (Fig EV1I and J).

These results indicate that endosomal PI(3)P synthesis is repressed by neuronal activity via Cdk5‐mediated feedback regulation of VPS34.

### PI(3)P depletion reduces neurotransmission and perturbs SV endocytosis

**Figure 2:**
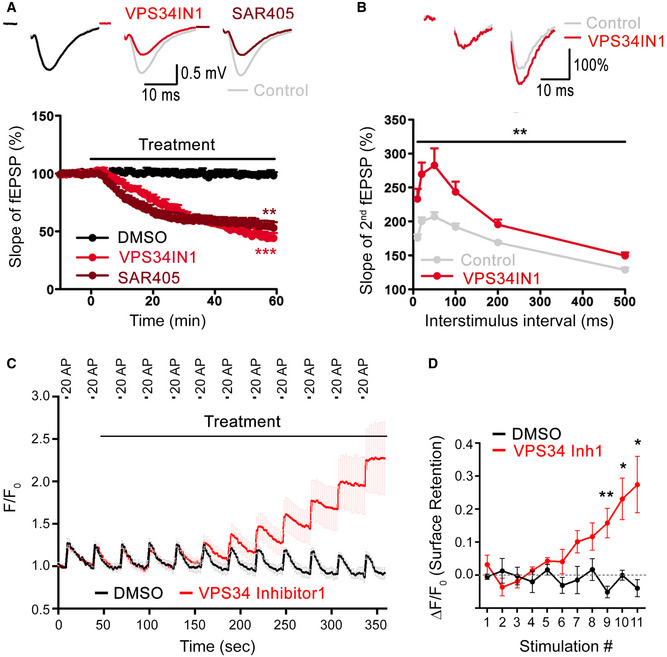
Loss of PI(3)P leads to reduced basal synaptic transmission and perturbs SV endocytosis Basal excitatory synaptic transmission of CA3‐CA1 connections in acute hippocampal slices treated with DMSO (0.03%) or the VPS34 inhibitors, VPS34IN1 (3 µM) or SAR405 (20 µM). Representative fEPSP traces (above) and the relative slope of fEPSPs (below) are shown. VPS34 inhibition causes reduced excitatory synaptic transmission down to 46.0 ± 4.1% and 55.0 ± 4.4% for VPS34IN1 and SAR405 at the end of 60‐min treatment. *N* = 6 slices per condition from ≥ 4 mice; Student’s *t*‐test.Increased paired paired‐pulse facilitation (PPF) at hippocampal CA3‐CA1 synapses after VPS34 inhibition. Representative traces of fEPSPs with a 20 ms interpulse interval is shown above. The ratio of the slope of the second to the first response over a range of interstimulus intervals (10–500 ms) is plotted below, indicating an increased facilitation of the second response in VPS34IN1‐treated acute hippocampal slices. PI(3)P depletion causes a significant facilitation of the second fEPSP, indicative of reduced initial release probability. *N* = 6 slices per condition from 6 mice; Two‐way ANOVA.Synaptophysin‐pHluorin responses normalized to baseline from neurons stimulated with 12 consecutive trains of 20 APs (20 Hz, 1 s) with 30 s gap between each stimulus. Neurons are acutely treated with DMSO (0.1%) or VPS34 inhibitor1 (50 µM) starting from the onset of 2^nd^ stimulation. The timing of the treatment and the stimulation trains are indicated.Quantification of data from C. Net difference of Synaptophysin‐pHluorin fluorescence 2 s before and 28 s after each stimulation (2 s before the next stimulation) to estimate relative surface retention of SVs. SV endocytosis is blocked within 5 min of treatment with VPS34 Inhibitor1, leading to progressive accumulation of Synaptophysin‐pHluorin signal. *N* = 4 or 5 independent measurements per condition; Student’s *t*‐test. Data information: Data presented as mean ± SEM in all panels; *P < 0.05; **P < 0.01; ***P < 0.001; p, t, q, df values are provided in the Source Data Statistics file. Source data are available online for this figure.

To analyze the functional consequences of reduced PI(3)P synthesis at synapses, we probed the effects of pharmacological inhibition of VPS34‐mediated PI(3)P synthesis on neurotransmission by electrophysiological recordings in acute hippocampal slice preparations from genetically unperturbed wild‐type mice. Depletion of PI(3)P in the presence of VPS34IN1 resulted in a rapid and progressive decline of field excitatory postsynaptic potentials (fEPSP) (Figs 2A, Appendix Fig S1A and B). This was paralleled by an increased paired pulse ratio (Figs 2B and Appendix Fig S1C), a surrogate measure of presynaptic release probability. A similar depression of basal excitatory neurotransmission (Fig 2A) and elevated paired‐pulse response (Appendix Fig S1D) was observed upon application of another VPS34 inhibitor, SAR405 (Ronan *et al*, 2014). These data indicate that repression of VPS34‐mediated PI(3)P synthesis reduces basal excitatory neurotransmission in response to depolarizing stimuli, likely via alterations in presynaptic release probability.

**Figure 3:**
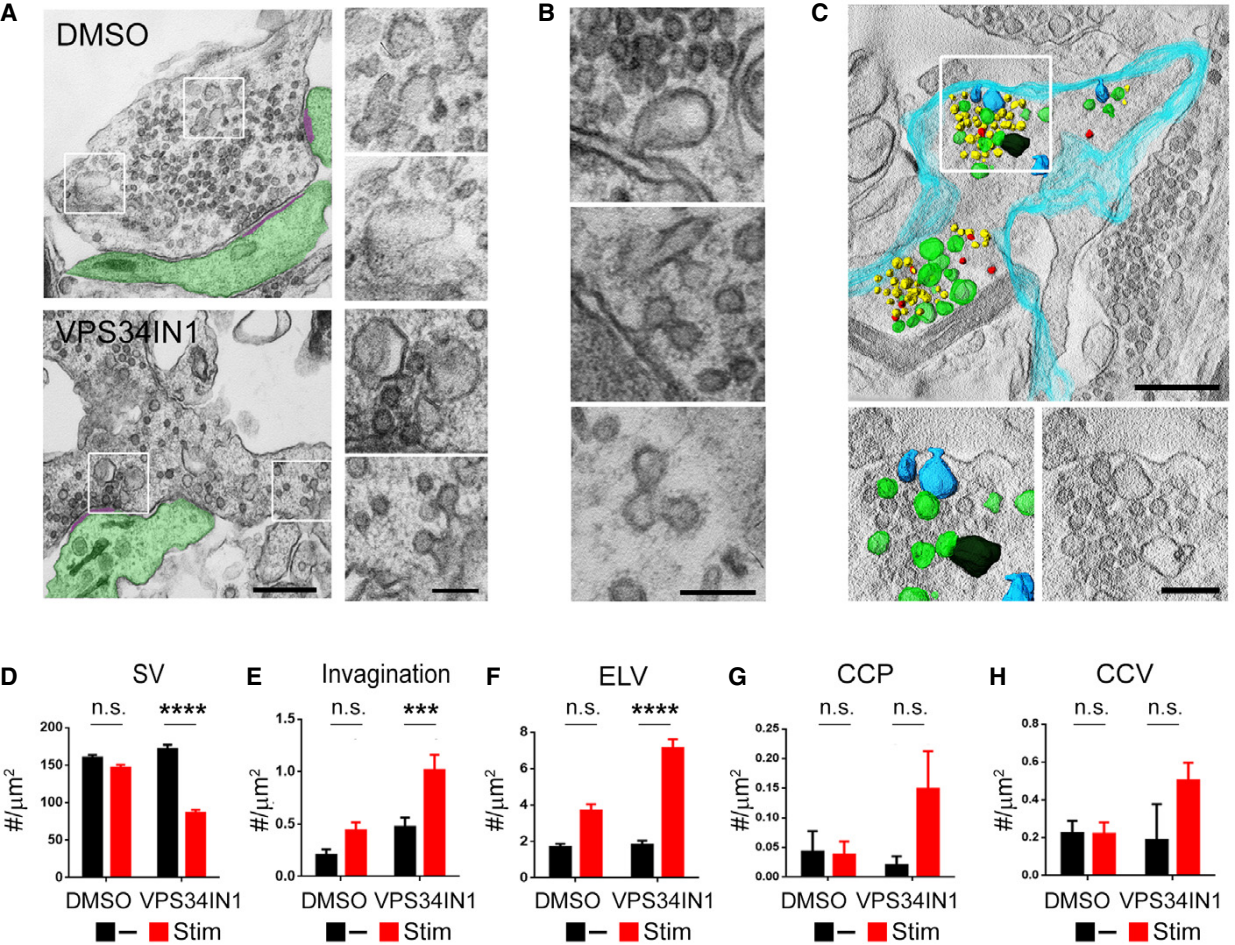
Loss of PI(3)P leads to accumulation of SV recycling intermediates in stimulated synapses AElectron micrographs of VPS34IN1‐ and DMSO‐treated synapses following four consecutive trains of 200 AP (40 Hz, 5 s; 90 s gap between each train) stimulation. The postsynaptic compartment and the postsynaptic density are highlighted in green and purple, respectively. Note the reduced SV numbers and plasma membrane‐derived endocytic intermediates in VPS34IN1‐treated synapses. Scale bars, 500 nm (left) and 200 nm (right).BHigh magnification views of endocytic intermediates accumulated in stimulated hippocampal neurons treated with VPS34IN1. Scale bar, 200 nm.C3D‐reconstruction of a synaptic terminal treated with VPS34IN1 and stimulated as in A. The plasma membrane is colored in turquoise. Synaptic vesicles (yellow), plasma membrane‐derived invaginations (blue), endosome‐like vacuoles (green), and CCVs (red) are highlighted. Scale bar, 500 nm (top) and 200 nm (bottom).D–HMorphometric quantification of data shown in A. Synapses of VPS34IN1‐treated stimulated neurons display reduced numbers of SVs/ bouton area in (D) and an accumulation of endocytic membrane invaginations (E), endosome like vacuoles (ELV) (F), CCPs (G), and CCVs (H). *n* = 100 (SV) and *n* = 200 (invagination, ELV, CCP, CCV) profiles per condition; Two‐way ANOVA; Tukey’s Multiple Comparison Test. Data information: Data presented as mean ± SEM in all panels; n.s. not significant; ***P < 0.001; ****P < 0.0001; p, t, q, df values are provided in the Source Data Statistics file. Source data are available online for this figure.

**Figure 4:**
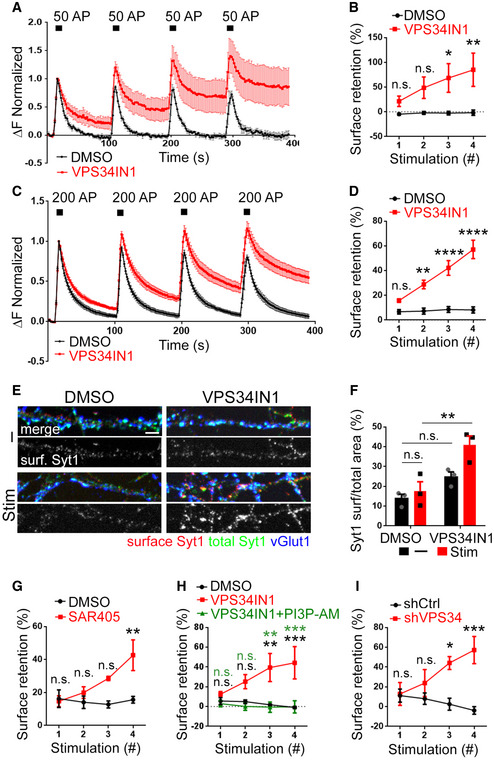
Depletion of PI(3)P impairs SV endocytosis in stimulated synapses A–DNormalized Synaptophysin‐pHluorin responses from neurons treated with VPS34IN1 (10 µM for 1 h prior to imaging) and stimulated with four consecutive trains of 50 APs (10 Hz, 5 s) in A or 200 APs (40 Hz, 5 s) in C. PI(3)P depletion in VPS34IN1‐treated neurons causes the progressive accumulation of Synaptophysin‐pHluorin on the neuronal surface in neurons stimulated with consecutive trains of 50 APs in B or 200 APs in D. Surface retention of Synaptophysin‐pHluorin 90 s poststimulus is plotted for each of the four successive stimulation trains. *N* ≥ 4 independent experiments; Two‐way ANOVA; Sidak’s Multiple Comparison Test.ECultured hippocampal neurons pretreated with DMSO (0.1%) or VPS34IN1 (10 µM, 1 h) were either field‐stimulated with four trains of 200 APs (40 Hz, 5 s; 90 s gap between each train) (Stim) or left unstimulated (−), fixed and stained for surface‐stranded Synaptotagmin‐1, total Synaptotagmin‐1, and vGlut1. Scale bar, 10 µm.FFraction of endogenous Synaptotagmin‐1 stranded on the surface of presynaptic nerve terminals from the images shown in E. *N* = 3 independent experiments (≥ 40 images per condition); Two‐way ANOVA; Tukey’s Multiple Comparison Test.GSurface retention of Synaptophysin‐pHluorin 90 s poststimulus is plotted for each of the four successive 200 AP stimulation trains in neurons treated with SAR405 (20 µM). Data are similar to those shown in C and D. *N* = 3 independent experiments (≥ 11 images per condition); Two‐way ANOVA; Sidak’s Multiple Comparison Test.HExogenous supply of membrane‐permeant PI(3)P acutely rescues defective Synaptophysin‐pHluorin endocytosis induced by pharmacological inhibition of VPS34‐mediated PI(3)P synthesis in hippocampal neurons. Data are similar to those shown in C and D; where indicated exogenous PI(3)P‐AM (20 µM) was supplied in parallel to VPS34IN1 (10 µM) for 1 h prior to imaging. *N* = 3 independent experiments (15 images per condition); Two‐way ANOVA; Tukey’s Multiple Comparison Test.IKinetic block of Synaptophysin‐pHluorin endocytosis at hippocampal synapses depleted of VPS34. Hippocampal neurons depleted of endogenous VPS34 by specific shRNA (shVPS34) or treated with nontargeting control shRNA (shCtrl) and stimulated as in C and analyzed as in D. *N* = 3 independent experiments (≥ 10 images per condition); Two‐way ANOVA; Sidak’s Multiple Comparison Test. Data information: Data presented as mean ± SEM in all panels; n.s. not significant; *P < 0.05; **P < 0.01; ***P < 0.001; ****P < 0.0001; p, t, q, df values are provided in the Source Data Statistics file. Source data are available online for this figure.

As VPS34 operates mainly on endosomes and PI(3)P is absent from the plasma membrane (Gaullier *et al*, 1998; Di Paolo & De Camilli, 2006; Balla, 2013; Morel *et al*, 2013; Raiborg *et al*, 2013; Ketel *et al*, 2016), we speculated that reduced basal neurotransmission in PI(3)P‐depleted neurons might be accompanied or partially caused by impaired SV endocytosis and/ or recycling, which may involve endosome‐like intermediates (Rizzoli, 2014; Watanabe *et al*, 2014; Jahne *et al*, 2015; Chanaday *et al*, 2019). We tested this by monitoring SV endocytosis using the pH‐sensitive reporter Synaptophysin‐pHluorin, a chimera between pH‐sensitive super‐ecliptic GFP and the SV protein Synaptophysin (Miesenbock *et al*, 1998; Kavalali & Jorgensen, 2014). Pharmacological inhibition of endosomal PI(3)P synthesis caused a progressive impairment of Synaptophysin‐pHluorin endocytosis in hippocampal neurons stimulated with brief trains of APs (20APs, 20 Hz) (Fig 2C). Delayed kinetics of Synaptophysin‐pHluorin retrieval were paralleled by a pronounced retention of nonretrieved Synaptophysin‐pHluorin molecules on the neuronal surface (Fig 2D). Under these conditions of repeated AP train stimulation (see also Fig 4A–D) no overt defect in SV exocytosis was observed, suggesting that AP train‐induced presynaptic calcium elevation can override a reduction in release probability caused by depletion of PI(3)P. To corroborate these results from optical imaging experiments, we analyzed the ultrastructure of synapses from VPS34IN1‐treated hippocampal neurons stimulated with trains of 200 APs or kept at rest by electron microscopy (EM) and quantitative morphometry and by tomographic 3D reconstructions (Fig 3). Synapses from stimulated neurons depleted of PI(3)P by application of VPS34IN1 displayed a profound reduction in the SV pool size (Fig 3A and D) and an accumulation of endocytic plasma membrane invaginations (Fig 3B and E) and endosome‐like vacuoles (Fig 3C and F), in addition to clathrin‐coated endocytic pits and vesicles (Fig 3B, G and H) compared to DMSO‐treated controls. No significant SV depletion or accumulation of unresolved intermediates of SV endocytosis were observed in nonstimulated hippocampal neurons (Fig 3D–H).

These data show that PI(3)P depletion perturbs SV endocytosis and reduces presynaptic release probability.

### Depletion of endosomal PI(3)P impairs SV endocytosis via a mechanism that depends on neuronal activity

**Figure EV2:**
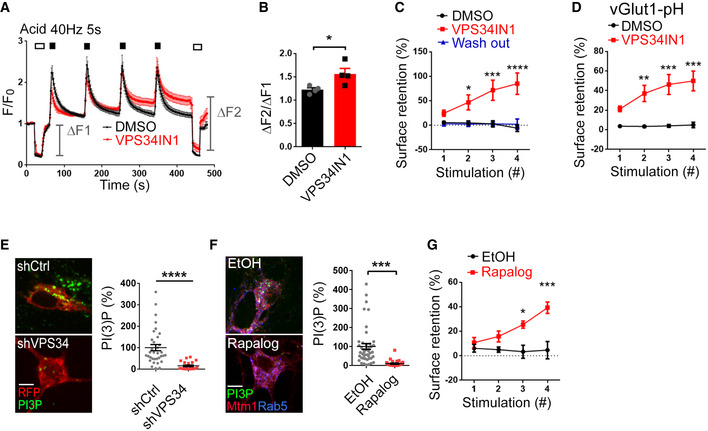
PI(3)P depletion inhibits SV endocytosis monitored by Synaptophysin‐pHluorin Normalized Synaptophysin‐pHluorin responses in cultured hippocampal neurons stimulated with four trains of 200 APs (40 Hz, 5 s) with 90 s intervals after 1 h pretreatment with DMSO or VPS34IN1 (10 µM) and subjected to low pH image buffer (Acid) before and after the stimulation trains.The ratio of surface fluorescence of Synaptophysin‐pHluorin before and after 4 × 200 APs stimulation in DMSO‐ and VPS34IN1‐treated neurons. Mean ± SEM; 15 images per condition from 4 independent experiments; *P < 0.05; Student’s *t*‐test.Surface retention of Synaptophysin‐pHluorin 90 s poststimulus is plotted for each of the four successive 200 AP stimulation trains in neurons treated with VPS34IN1 (10 µM, 1 h) and washed overnight with conditioned medium. Mean ± SEM; ≥ 11 images per condition from 3 independent experiments; *P < 0.05; ***P < 0.001; ****P < 0.0001; Two‐way ANOVA.Surface retention of vGlut1‐pHluorin 90 s poststimulus plotted for each 200 AP stimulation (40 Hz, 5 s) in neurons treated with VPS34IN1 (10 µM, 1 h). Mean ± SEM; ≥ 15 images per condition from 3 independent experiments; **P < 0.01; ***P < 0.001; Two‐way ANOVA.Cultured hippocampal neurons transfected with mRFP and control shRNA or shRNA against VPS34, stained for PI(3)P. Scale bar, 10 µm. Mean ± SEM; *n* = 34 (shCtrl) and *n* = 21 (shVPS34); ****P < 0.0001; Student’s *t*‐test.Confocal images of cultured hippocampal neurons transfected with mRFP‐FKBP‐hMTM1 and FRB*‐iRFP‐Rab5, treated with EtOH or Rapalog and stained for PI(3)P. Scale bar, 10 µm. Mean ± SEM; ≥ 35 images per condition; ***P < 0.001; Student’s *t*‐test.Surface retention of Synaptophysin‐pHluorin 90 s poststimulus is plotted for each of the four successive 200 AP stimulation trains in hippocampal neurons expressing mRFP‐FKBP‐hMTM1 and FRB*‐iRFP‐Rab5 and treated with EtOH or Rapalog. Mean ± SEM; ≥ 13 images per condition from 3 independent experiments; *P < 0.05; ***P < 0.001; Two‐way ANOVA. Source data are available online for this figure.

We reasoned that the observed defects in SV endocytosis in hippocampal neurons depleted of PI(3)P likely originate at internal, that is, endosomal compartments known to play a role in SV recycling in response to sustained stimulation (Saheki & De Camilli, 2012; Rizzoli, 2014; Wu *et al*, 2014; Chanaday *et al*, 2019). We therefore tested whether PI(3)P depletion impairs SV endocytosis not only in response to brief AP trains (Fig 2C and D) but also under conditions of sustained high‐frequency stimulation. Pretreatment of Synaptophysin‐pHluorin‐expressing hippocampal neurons with VPS34IN1 (10 µM, 1 h) followed by repetitive strong stimulation with trains of 50 APs or 200 APs resulted in a progressive kinetic delay in SV endocytosis (Fig 4A and C) and an accumulation of nonendocytosed Synaptophysin‐pHluorin molecules on the neuronal surface (Fig 4B and D), as further evidenced by their accessibility to externally added low pH buffer (Fig EV2A and B). A similar stimulation‐induced surface accumulation was observed for the endogenous SV protein Synaptotagmin 1 (Sudhof & Rothman, 2009) (Fig 4E and F) or if pHluorin‐tagged vesicular glutamate transporter 1 (vGlut1) was used as a reporter (Fig EV2D). Washout of the drug (Fig EV2C) or restoring PI(3)P levels by bath application of synthetic cell permeable PI(3)P (Subramanian *et al*, 2010) reversed the phenotype (Fig 4H). The kinetics of SV endocytosis was also perturbed upon VPS34 inhibition by SAR405 (Fig 4G). As pharmacological inhibitors may display off‐target effects, we challenged our findings by two further independent approaches. First, we depleted neurons of VPS34 kinase by shRNA‐mediated knockdown, which led to profoundly reduced PI(3)P levels (Fig EV2E). Second, we acutely depleted PI(3)P from Rab5‐positive neuronal endosomes by Rapalog‐induced recruitment of the PI(3)P 3‐phosphatase MTM1 (Fili *et al*, 2006) (Fig EV2F). Either of these treatments caused a progressive kinetic delay of SV endocytosis (Figs 4I and EV2G) akin to pharmacological inhibition of VPS34‐mediated PI(3)P synthesis. These collective data demonstrate that genetic or pharmacological depletion of endosomal PI(3)P impairs SV endocytosis in response to either brief AP trains (Fig 2) or sustained repetitive high‐frequency stimulation (Figs 3 and 4).

**Figure 5:**
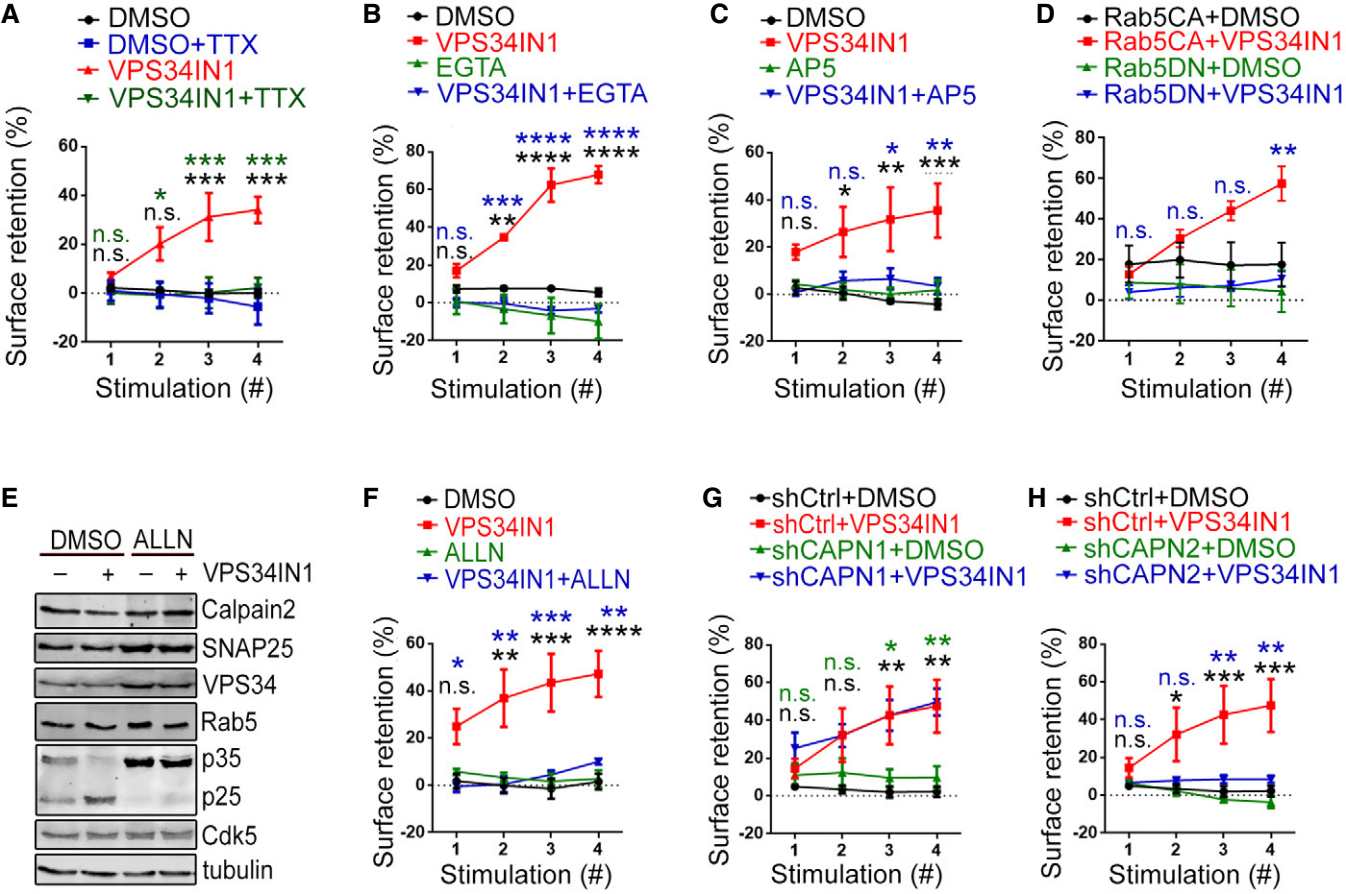
Impaired SV endocytosis upon PI(3)P depletion is activity‐ and Calpain 2‐dependent A–CThe kinetic block of SV endocytosis at excitatory synapses depleted of PI(3)P depends on prior neuronal activity and Ca^2+^. VPS34IN1‐induced defects in SV endocytosis are reverted by parallel treatment with TTX (0.2 µM) in A, EGTA (2 mM) in B and AP5 (20 µM) in C, in Synaptophysin‐pHluorin‐expressing neurons stimulated and analyzed as in Fig 4C and D. *N* = 3 independent experiments (≥ 10 images per condition); Two‐way ANOVA; Tukey’s Multiple Comparison Test.DOverexpression of dominant‐negative Rab5 (Rab5DN), but not constitutively active Rab5 (Rab5CA), rescues the progressive accumulation of Synaptophysin‐pHluorin on the surface of VPS34IN1‐treated hippocampal neurons stimulated and analyzed as in A–C. *N* = 3 independent experiments (≥ 10 images per condition); Two‐way ANOVA; Tukey’s Multiple Comparison Test.ELevels of various synaptic proteins in DMSO (0.1%) and VPS34IN1 (10 µM) treated cultured cerebellar granule neurons analyzed by western blot. VPS34 inhibition results in p35 processing to hyperactive p25, a reaction blocked by Calpain inhibitor ALLN (100 µM).F–HCalpain inhibition by ALLN (100 µM) in F or Calpain 2 knockdown via lentiviral shRNA (shCAPN2) in H rescue the progressive accumulation of Synaptophysin‐pHluorin on the neuronal surface induced by VPS34IN1. Calpain 1 knockdown via lentiviral shRNA (shCAPN1) in G had no impact on VPS34IN1‐induced kinetic delay of SV endocytosis. Data are similar to those shown in A–C. *N* = 3 independent experiments (≥ 11 images per condition); Two‐way ANOVA; Tukey’s Multiple Comparison Test. Data information: Data presented as mean ± SEM in all panels; n.s. not significant; *P < 0.05; **P < 0.01; ***P < 0.001; ****P < 0.0001; p, t, q, df values are provided in the Source Data Statistics file. Source data are available online for this figure.

What might be the molecular explanation for the requirement for endosomal PI(3)P for SV endocytosis? One possibility is that depletion of PI(3)P perturbs SV endocytosis because PI(3)P regulates so far unknown components of the SV endocytosis machinery. Given that VPS34 and its product PI(3)P are not required for endocytosis in non‐neuronal cells (Di Paolo & De Camilli, 2006; Balla, 2013; Raiborg *et al*, 2013; Ketel *et al*, 2016), we followed the alternative hypothesis that PI(3)P levels impact on the activity or nanoscale distribution of cell surface signaling molecules, for example, receptors, that might regulate directly or indirectly neurotransmission and SV cycling. We therefore examined if the effects of VPS34IN1‐mediated loss of PI(3)P on SV endocytosis depend on prior network activity. To this aim, we pre‐incubated hippocampal neurons simultaneously with VPS34IN1 and the activity blocker TTX (Fig 5A) or the calcium chelator EGTA (Fig 5B). Strikingly, application of either TTX or EGTA occluded the inhibitory effect of PI(3)P depletion on SV endocytosis. Given the activity and calcium dependence of this phenotype, we analyzed next whether NMDA receptor activity, a major postsynaptic regulator of excitatory neurotransmission, is involved in the pathway. Blockade of NMDA receptor activity with AP5 led to a near full rescue of the effects of VPS34IN1 on SV endocytosis (Fig 5C). Finally, VPS34 inhibition also failed to impair SV endocytosis in hippocampal neurons expressing a dominant‐negative version of the endosomal protein Rab5 (Fig 5D), a Ras‐related GTPase that is a fundamental component of endocytic recycling and intracellular trafficking of cell surface receptors and known to be hyperactivated upon depletion of PI(3)P (Law *et al*, 2017).

These results identify a PI(3)P‐dependent pathway that controls SV cycling via a mechanism that depends on neuronal activity, NMDA receptors, and the endosomal GTPase Rab5.

### Impaired SV endocytosis and reduced neurotransmission upon depletion of PI(3)P are caused by Calpain 2‐mediated hyperactivation of Cdk5

**Figure EV3:**
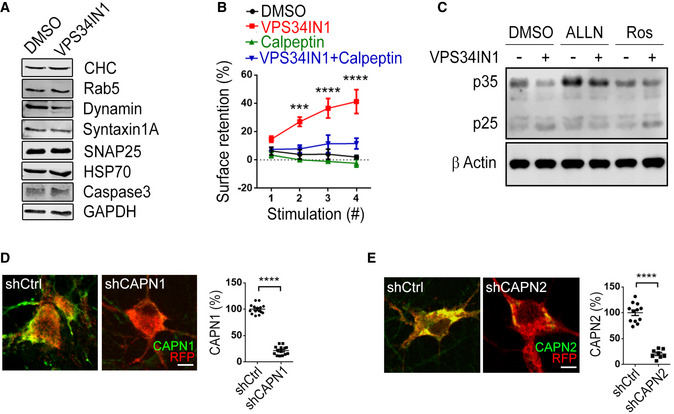
PI(3)P loss inhibits SV endocytosis via calpain 2 activation ALevels of various synaptic proteins in DMSO (0.1%) and VPS34IN1 (10 µM) treated cultured cerebellar granule neurons analyzed by western blot.BSurface retention of Synaptophysin‐pHluorin 90 s poststimulus is plotted for each of the four successive stimulation trains in neurons treated with VPS34IN1 (10 µM) and Calpeptin (10 µM). Mean ± SEM; ≥ 9 images per condition from 3 independent experiments; ***P < 0.001; ****P < 0.0001; Two‐way ANOVA.CProcessing of p35 to p25 in cultured hippocampal neurons treated with VPS34IN1 (10 µM) for 1 h, analyzed by western blot. Cleavage of p35 to p25 was reduced in neurons simultaneously treated with ALLN (100 µM) but not with Roscovitine (Ros, 10 µM).D, ECultured hippocampal neurons were cotransfected with an mRFP expression plasmid and shCtrl, shCAPN1, or shCAPN2 and stained for Calpain 1 in D or Calpain 2 in E. Scale bar, 10 µm. The relative mean fluorescence intensity is plotted. ≥ 8 images per condition; ****P < 0.0001; Student’s *t*‐test. Source data are available online for this figure.

An accumulation of endocytic pits and plasma membrane invaginations akin to those observed in PI(3)P‐depleted synapses (Fig 3) has previously been observed at synapses from dynamin 1 single or dynamin 1/3 double knockout mice defective in SV endocytosis (Ferguson *et al*, 2007; Raimondi *et al*, 2011). We therefore hypothesized that the activity‐dependent kinetic delay of SV endocytosis in PI(3)P‐depleted neurons may result from the dysregulation of a signaling pathway that impinges on the endocytic protein dynamin or its regulators. To identify the molecules involved in this pathway, we resorted to the biochemical analysis of the effects of PI(3)P depletion in cerebellar granule neurons (CGNs) that can be cultured in the virtual absence of other cell types, for example, astrocytes. Treatment of CGNs with VPS34IN1 did not alter the levels of major exocytic and endocytic proteins including Syntaxin 1A, SNAP‐25, Dynamin, or Clathrin (Fig EV3A). Instead, we found a prominent change in the ratio of the p35/p25 subunit of Cdk5 (Fig 5E), a synaptic protein kinase that negatively regulates SV recycling via phosphorylation of dynamin 1 (Tan *et al*, 2003; Ferguson *et al*, 2007; Armbruster *et al*, 2013) and represses presynaptic neurotransmission (Kim & Ryan, 2010, 2013; Rothman *et al*, 2016). In PI(3)P‐depleted neurons, p35•Cdk5 appeared to be proteolytically converted to the hyperactive p25•Cdk5 isoform, a reaction known to be catalyzed by the calcium‐dependent protease Calpain (Shah & Lahiri, 2014). Consistent with this, we observed that specific pharmacological inhibition of Calpain by ALLN (Baudry & Bi, 2016) prevented p35‐to‐p25 conversion in CGNs (Fig 5E) and in hippocampal neurons (Fig EV3C). Importantly, ALLN completely occluded the inhibitory effect of PI(3)P depletion on SV endocytosis (Fig 5F). Occlusion of VPS34IN1‐mediated inhibition of SV endocytosis was also observed, if Calpain activity was blocked by Calpeptin (Fig EV3B) or depletion of Calpain 2, but not Calpain 1 by shRNA‐mediated knockdown (Figs 5G and H, and EV3D and E).

**Figure 6:**
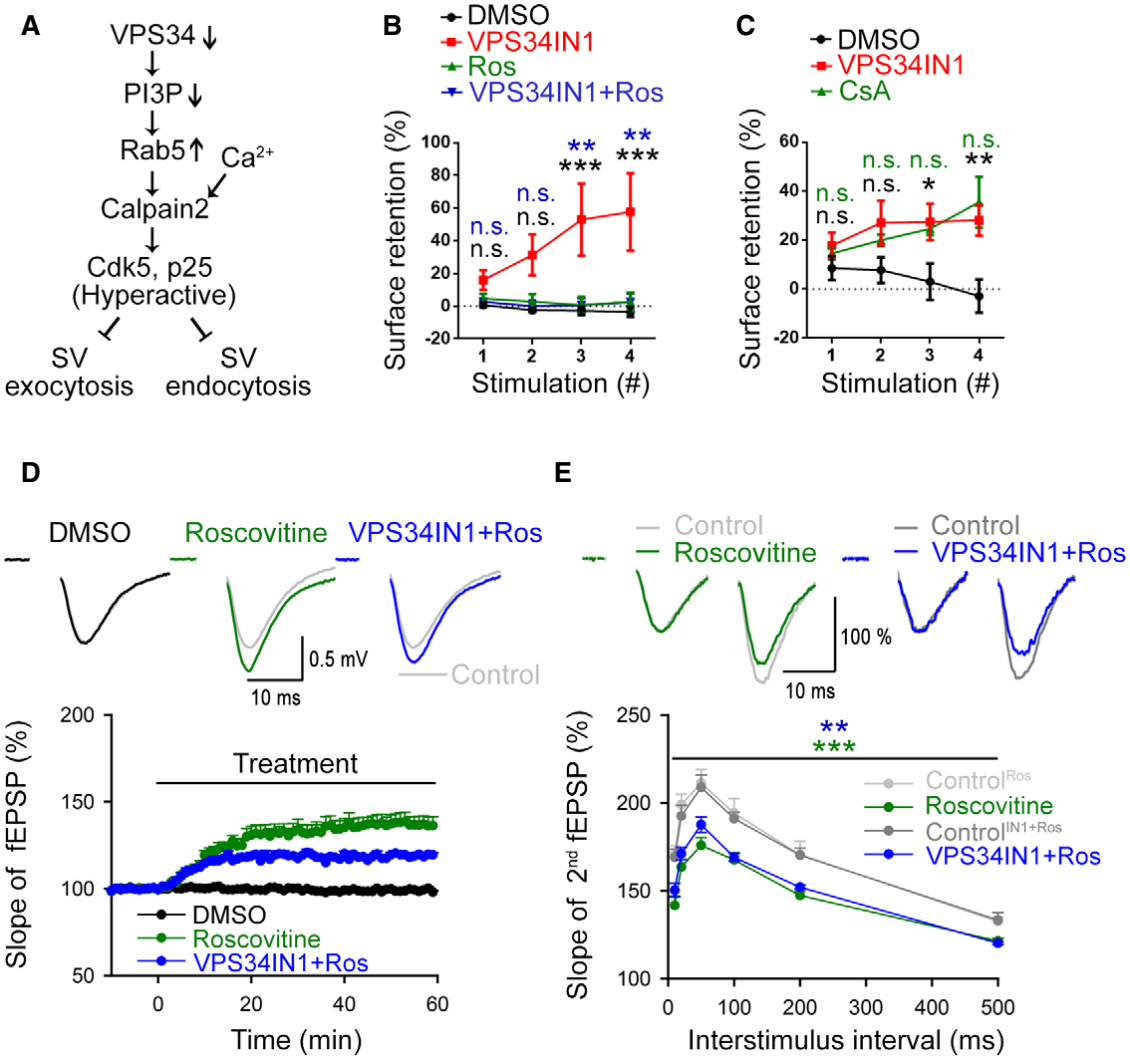
Reduced neurotransmission and block of SV endocytosis due to PI(3)P depletion is Cdk5‐dependent Hypothetical signaling pathway for the induction of hyperactive Cdk5 induced by repression of VPS34 downstream of neuronal activity.Cdk5 inhibition by Roscovitine (Ros, 10 µM) rescues the progressive accumulation of Synaptophysin‐pHluorin on the neuronal surface induced by VPS34IN1 (10 µM). Neurons are stimulated and analyzed as in Fig 4C and D. *N* = 4 independent experiments (20 images per condition); Two‐way ANOVA; Tukey’s Multiple Comparisons Test.Calcineurin inhibition by Cyclosporin A (10 µM) phenocopies VPS34IN1‐induced loss of PI(3)P with respect to the accumulation of Synaptophysin‐pHluorin on the neuronal surface after four successive stimulation trains (40 Hz, 5 s). Data were analyzed as in B. *N* = 3 independent experiments (15 images per condition); Two‐way ANOVA; Tukey’s Multiple Comparison Test.Basal excitatory synaptic transmission of CA3‐CA1 connections in acute hippocampal slices treated with DMSO (0.03%), Roscovitine (Ros, 10 µM) or VPS34IN1 (3 µM) plus Roscovitine. Representative fEPSP traces (above) and the relative slope of fEPSPs (below) are shown. Concomitant application of VPS34IN1 and Roscovitine facilitates excitatory synaptic transmission, akin to Roscovitine alone, and in contrast to VPS34IN1 alone (compare Fig 2A). *N* = 6 slices per condition from 6 mice; Student’s *t*‐test.Decreased paired paired‐pulse facilitation (PPF) at hippocampal CA3‐CA1 synapses treated with Roscovitine alone (Ros, 10 µM) or VPS34IN1 (3 µM) plus Roscovitine. Representative traces of fEPSPs with a 20 ms interpulse interval is shown above. The ratio of the slope of the second to the first response over a range of interstimulus intervals (10–500 ms) is plotted below. The data show decreased facilitation of the second response in Roscovitine or VPS34IN1 plus Roscovitine‐treated acute hippocampal slices, unlike VPS34IN1 alone (compare Fig 2B). *N* = 6 slices per condition from 6 mice; Two‐way RM ANOVA. Data information: Data presented as mean ± SEM in all panels; n.s. not significant; *P < 0.05; **P < 0.01; ***P < 0.001; p, t, q, df values are provided in the Source Data Statistics file. Source data are available online for this figure.

**Figure EV4:**
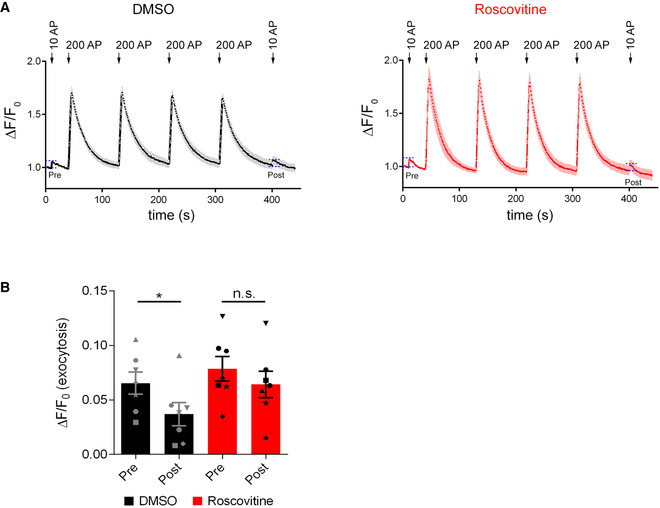
Repetitive high frequency stimulation reduces the responsiveness of synapses to mild physiological stimulation in a Cdk5‐dependent manner Normalized Synaptophysin‐pHluorin responses in cultured hippocampal neurons treated with DMSO (0.1%, left) or Roscovitine (10 µM; right) stimulated with 10 APs (40 Hz, 0.25 s), 4 × 200 APs (40 Hz, 5 s), and 10 APs (40 Hz, 0.25 s). The 10 AP stimulations before and after the train of 200 AP stimulations were labeled pre and post, respectively.The amplitude of SV release detected by measuring the increase in Synaptophysin‐pHluorin fluorescence in response to 10 AP before (pre) and after (post) high frequency stimulation (4 × 200 APs) as indicated by dashed lines in A. Repetitive high frequency stimulation reduces SV release, an effect occluded by Cdk5 inhibition. Mean ± SEM; *N* = 7 independent experiments; *P < 0.05; One‐way ANOVA; Tukey’s Multiple Comparison Test. Source data are available online for this figure.

Our data described thus far suggest that PI(3)P depletion via activation of Calpain 2 hyperactivates Cdk5 to repress SV endocytosis and excitatory neurotransmission (Fig 6A). We directly tested this model in hippocampal neurons in culture and in acute slice preparations by pharmacological inhibition of Cdk5. Cdk5 hyperactivation is known to kinetically impair SV endocytosis (Tan *et al*, 2003; Ferguson *et al*, 2007; Armbruster *et al*, 2013) and to reduce presynaptic neurotransmitter release (Kim & Ryan, 2010, 2013). If hyperactivation of Cdk5 would causally underlie defective SV endocytosis and reduced neurotransmission in VPS34IN1‐treated neurons, repression of its activity should rescue impaired SV endocytosis and neurotransmitter release. Pharmacological blockade of Cdk5 by the specific inhibitor Roscovitine (Tan *et al*, 2003; Shah & Lahiri, 2014) indeed rescued defective SV endocytosis under conditions of PI(3)P loss (Fig 6B). Conversely, the effects of VPS34IN1 on the kinetics of SV endocytosis in response to repetitive train stimulation were phenocopied by application of Cyclosporin A, a selective inhibitor of Calcineurin phosphatase activity known to counteract Cdk5 in the presynaptic compartment (Tan *et al*, 2003; Kim & Ryan, 2013) (Fig 6C). Blockade of Cdk5 activity by Roscovitine also largely occluded the adverse effects of VPS34IN1 on excitatory neurotransmission in acute hippocampal slice preparations. Instead, fEPSPs were facilitated in the presence of Roscovitine. This facilitatory effect of Roscovitine was similar albeit less pronounced than that seen in recordings from Roscovitine‐treated control slices lacking VPS34IN1 (Fig 6D). Furthermore, Roscovitine reverted the increase in paired pulse ratios induced by VPS34IN1 alone (Fig 2B) and, instead, caused paired‐pulse depression (Fig 6E). Finally, hippocampal neurons subjected to repetitive high frequency stimulation (4 × 200 APs), that is, conditions which repress endosomal PI(3)P synthesis (Fig 1F and G), displayed reduced synaptophysin‐pHluorin exocytosis in response to mild 10 AP stimuli. This train stimulation‐induced exocytic depression was occluded by inhibition of Cdk5 in the presence of Roscovitine (Fig EV4A and B).

These results from optical imaging and electrophysiological recordings in acute hippocampal slice preparations show that PI(3)P depletion impairs SV endocytosis and reduces excitatory neurotransmission via Calpain 2‐mediated hyperactivation of Cdk5.

### VPS34‐mediated endosomal PI(3)P synthesis is part of an autoregulated cell‐intrinsic pathway that controls SV recycling and neuronal network activity via Cdk5

**Figure EV5:**
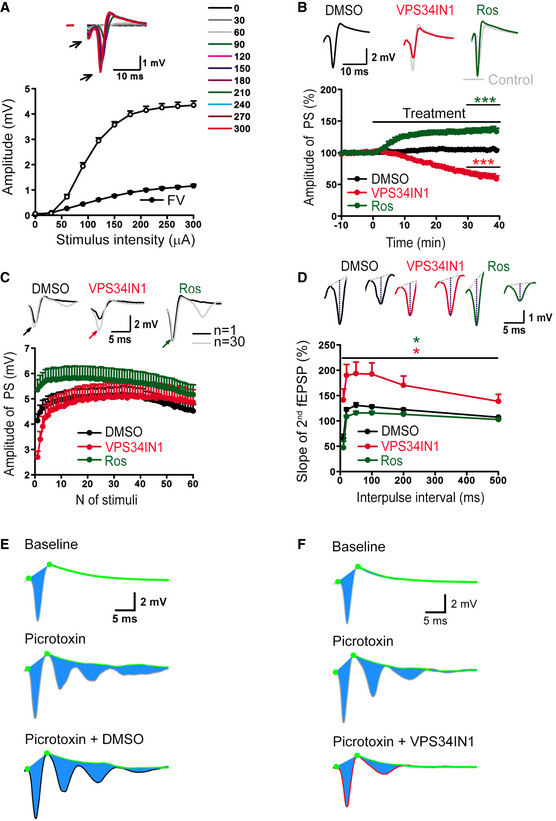
Effects of pharmacological inhibition of VPS34 and Cdk5 on hippocampal network activity AInput/output relationships between stimulus intensities and amplitudes of population spikes (PS) or fiber volleys (FV) show normal basal excitability of neuronal populations in CA1 *stratum pyramidale*. Insert sample (above) shows representative PS responses evoked by increasing stimulation strength (from 0 to 300 µA with a 30 µA step). Mean ± SEM; 36 slices from 12 mice.BRecordings of PS over time did not show significant changes in PS amplitudes in the presence of DMSO (0.03%). PS amplitude is reduced in the presence of VPS34IN1 (3 µM) and facilitated in the presence of Roscovitine (10 µM). Insert samples (above) show the average of 30 subsequent PSs before (from −10 to 0 min) and after (from 30 to 40 min) pharmacological treatments. Mean ± SEM; six slices per condition from six animals;***P < 0.001; One‐way ANOVA.CMeasurement of activity‐dependent facilitation of PSs. DMSO (0.03%) does not affect the facilitation of PS amplitudes during 1 Hz stimulation. VPS34IN1 treatment led to stronger facilitation of PS amplitudes, whereas facilitation was less pronounced in the presence of Roscovitine. Note that under all conditions 1 Hz stimulation eventually increased PS amplitudes to nearly identical maximal levels. Top panels show representative traces N1 (i.e., 1^st^ stimulus response) and N30 (i.e., 30^th^ stimulus response) with PS peaks indicated by color‐coded arrows. Mean ± SEM; 12 slices per condition from 12 animals.DRecordings of paired‐pulse modulation of PS at different interpulse intervals (from 10 to 500 ms). VPS34IN1 led to an elevated paired‐pulse ratio of PSs compared to DMSO, whereas Roscovitine reduced it. Mean ± SEM; 12 slices per condition from 12 animals; *P < 0.05; Two‐way ANOVA.E, FRepresentative traces show baseline averages of 30 traces before treatment (−10 to 0 min, top), after picrotoxin (50 µM) application (20–30 min, middle) and following treatment with DMSO or VPS34IN1 (60–70 min, bottom). Source data are available online for this figure.

**Figure 7:**
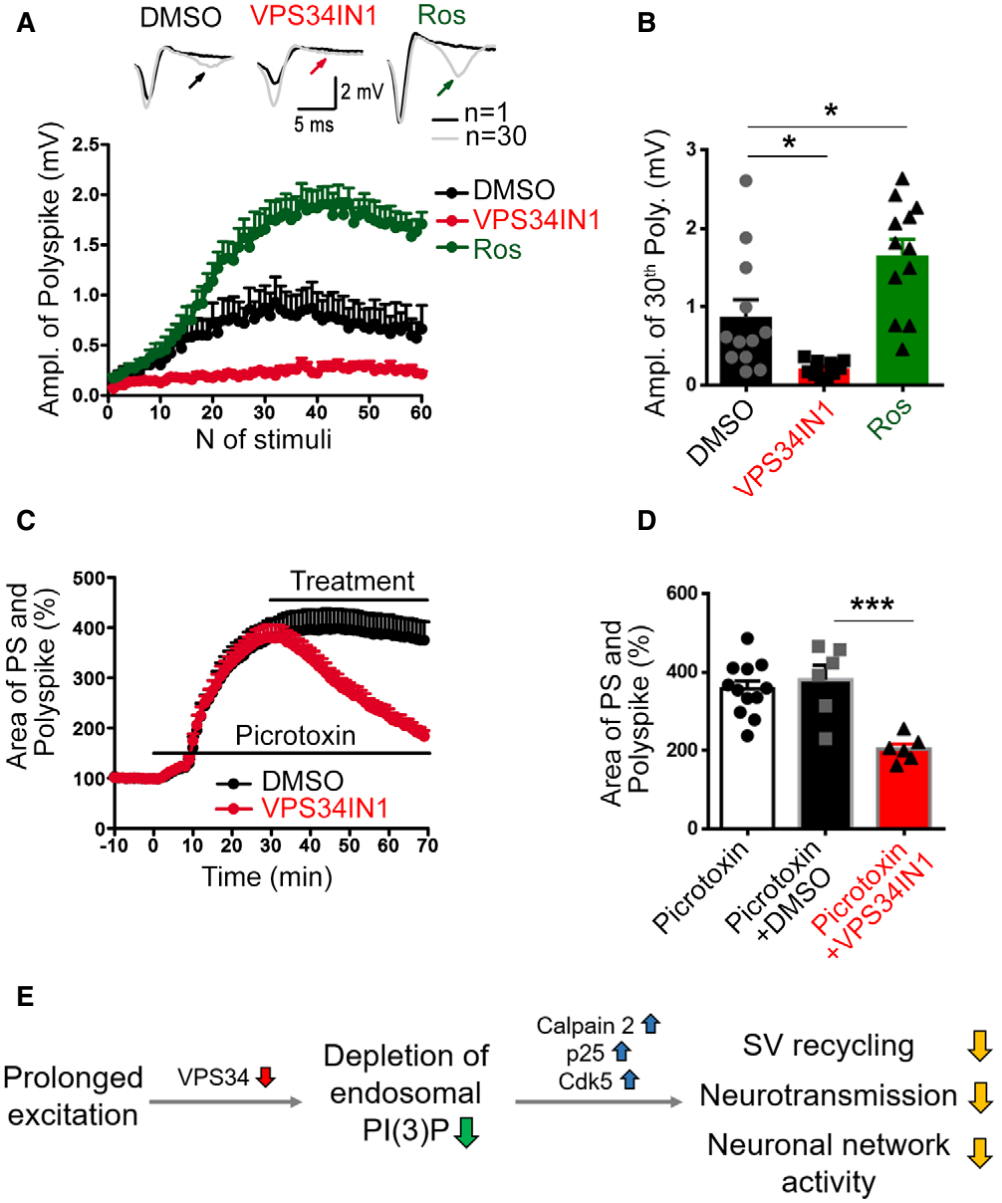
VPS34 is part of an autoregulated cell‐intrinsic pathway that controls neuronal network activity via Calpain 2, p25 and Cdk5 Activity‐dependent polyspiking of population spikes (PS) during 1 Hz stimulation is reduced by VPS34IN1 and facilitated by Roscovitine compared to DMSO‐treated control slices. Top, representative traces of the 1^st^ and 30^th^ stimulus responses with polyspikes indicated by arrows. *N* = 12 slices per condition from 12 mice.Quantification of polyspiking amplitudes in response to the 30^th^ stimulus in the presence of DMSO, VPS34IN1, or Roscovitine. Polyspiking amplitudes are increased by Roscovitine and reduced by VPS34IN1. *N* = 12 slices per condition from 12 mice; One‐way ANOVA.The relative changes in the PS and polyspike areas (i.e., the area indicated in blue in Fig EV5E and F) in the presence of picrotoxin (50 µM) and a subsequent treatment with DMSO or VPS34IN1 (10 µM). VPS34IN1 significantly reduced the PS and polyspike area compared to DMSO. *N* = 6 slices per condition from 6 mice.Mean area of PS and polyspikes in picrotoxin‐treated slices 40 min postapplication of DMSO or VPS34IN1. *N* ≥ 6 slices per condition from ≥ 6 mice; Student’s *t*‐test.A working‐model for the feedback regulation of synaptic activity. Prolonged neuronal excitation downregulates VSP34‐mediated synthesis of endosomal PI(3)P. PI(3)P depletion causes the activation of Calpain 2 and Cdk5, resulting in repression of SV recycling, neurotransmission and kinetic impairment of SV endocytosis to limit SV re‐supply and downregulate neuronal network activity. Data information: Data presented as mean ± SEM in all panels; *P < 0.05; ***P < 0.001. Source data are available online for this figure.

Depressed excitatory neurotransmission induced by activity‐dependent PI(3)P loss may be of particular importance to control and restrict neuronal network activity, for example, to prevent epileptic seizures under physiological or pathophysiological conditions. We tested this hypothesis by recording population spikes (PS) in CA1 *stratum pyramidale* (Fig EV5A), which are potently modulated by inhibitory transmission to prevent polyspiking. Depletion of PI(3)P in the presence of VPS34IN1 decreased baseline PS amplitudes, whereas Cdk5 inhibition by Roscovitine resulted in elevated PS amplitudes (Fig EV5B), akin to results from fEPSP recordings in *stratum radiatum*. To probe whether PI(3)P loss restricts excitatory neurotransmission when the inhibitory network is impaired, we disinhibited hippocampal networks and measured polyspiking to probe neuronal network excitability. Activity‐dependent disinhibition induced by 1 Hz stimulation revealed a profound repression of polyspiking activity in VPS34IN1‐treated slices, while Roscovitine treatment facilitated polyspiking (Fig 7A and B). During the 1 Hz stimulation train, the PS amplitudes were facilitated to similar values under all conditions (Fig EV5C). Hence, the opposite effects of VPS34 *vs*. Cdk5 inhibition on polyspiking were not an indirect consequence of the altered baseline PS responses (Fig EV5B). Next, we determined whether the opposing effects of VPS34IN1 and Roscovitine on polyspiking activity might result from altered synaptic inhibition of local neuronal networks. To this aim, we probed the regulation of PS amplitudes via feedback inhibition using a paired stimulation protocol, in which the amplitude of the second PS is modulated by synaptic feedback inhibition. Measurements of paired PS amplitudes did not reveal evidence for reduced feedback inhibition. In fact, paired PS ratios rather than being facilitated were reduced by Roscovitine treatment (Fig EV5D), in agreement with the effects of Roscovitine on baseline PS amplitudes (Fig EV5B) and paired‐pulse facilitation (Fig 6E). In contrast, VPS34IN1 treatment led to elevated paired PS ratios (Fig EV5D), consistent with the reduced baseline PS amplitudes (Fig EV5B) and increased paired‐pulse facilitation of synaptic transmission (Fig 2B). These data suggest that loss of endosomal PI(3)P restricts neuronal network activity by repressing excitatory neurotransmission, independent of synaptic inhibition. To further challenge this hypothesis, we induced polyspike waves by applying the GABA_A_ receptor antagonist Picrotoxin to block inhibitory transmission (Fig EV5E and F). Acute inhibition of VPS34‐mediated PI(3)P synthesis potently repressed the strong polyspiking activity under these conditions (Fig 7C and D).

These data show that endosomal PI(3)P depletion restricts neuronal network activity independent of the effects of synaptic inhibition. Collectively, our findings suggest a crucial hitherto unknown function for endosomal PI(3)P in the control of SV recycling and neuronal network activity (Fig 7E).

## Discussion

Our findings reported here suggest that neurotransmitter release and vesicle cycling are cell‐intrinsically controlled by an autoregulatory pathway that is based on the endosomal lipid PI(3)P. Repression of PI(3)P synthesis by VPS34 on endosomes is shown to trigger the calcium‐ and Calpain‐driven activation of Cdk5, resulting in reduced neurotransmission and impaired SV endocytosis. The VPS34‐Cdk5 pathway appears to be feedback‐controlled by Cdk5 itself as pharmacological inhibition of Cdk5 (Figs 1K and EV1F) prevents the activity‐induced repression of VPS34‐mediated PI(3)P synthesis on endosomes in hippocampal neurons. We, thus, hypothesize that endosomal VPS34 and Cdk5 are part of an autoregulated signaling circuit (Fig 7E) that limits neurotransmission and SV cycling within neuronal networks to prevent hyperexcitation and epileptiform activity. We speculate this VPS34‐Cdk5 pathway that may contain additional elements such as lipid phosphatases, to be part of a hypothetical two‐stage emergency response system, in which excessive neurotransmitter release is first counteracted by Calpain 2‐mediated activation of p25•Cdk5 to restrict SV exo‐endocytosis. If hyperactive neuronal signaling persists, such neurons may eventually be eliminated by Calpain 2‐triggered apoptosis to restore neural network function. Consistent with this scenario, it has been shown that neuronal loss of VPS34 results in severe neurodegeneration *in vivo* (Zhou *et al*, 2010), a phenotype linked to Caspase activation downstream of active Calpain (Baudry & Bi, 2016). From a neural network perspective, the endosomal VPS34‐Cdk5 pathway thus plays a complementary function to homeostatic scaling, a process in which excitatory neurotransmitter release is facilitated under conditions of reduced postsynaptic excitability to homeostatically restore neural circuit activity (Davis & Goodman, 1998; Turrigiano & Nelson, 2004; Davis & Muller, 2015; Delvendahl *et al*, 2019). While our analysis focuses on the study of neurotransmission in the hippocampus, we postulate that the basic principles of the autoregulatory VPS34‐Cdk5 signaling circuit are conserved in other networks, for example, in the neocortex and in the cerebellum.

At present, several open questions remain regarding the precise molecular machinery involved in this pathway. For example, it is unclear how exactly PI(3)P depletion results in the activity‐ and calcium‐dependent activation of Calpain 2. One possibility is that PI(3)P levels affect the localization and/ or activity of calcium‐permeant NMDA receptors or other types of calcium channels. Consistently, we find that pharmacological inhibition of NMDA receptor activity occludes the adverse effects of PI(3)P depletion on SV cycling (Fig 5C). Alternatively, PI(3)P may alter calcium homeostasis or release from internal stores such as the endoplasmic reticulum that is linked to endosomes via membrane contact sites (Wu *et al*, 2018). A third possibility is that PI(3)P directly or indirectly affects the localization of Calpain 2 itself. Such a scenario is supported by data from non‐neuronal cells suggesting that Calpain 2 activation is mediated by Rab5‐mediated recruitment to early endosomes (Mendoza *et al*, 2018), that is, organelles that contain PI(3)P. These scenarios will need to be studied in detail in the future. Second, given the multiple roles of Cdk5 in presynaptic neurotransmission and SV cycling, it is possible that hyperactivation of Cdk5 impinges not only on SV endocytosis (Tan *et al*, 2003; Armbruster *et al*, 2013) but may involve additional target proteins such as voltage‐gated calcium channels (Kim & Ryan, 2010), crucial factors for the regulation of presynaptic release probability. Consistently, we observe that AP train‐induced presynaptic calcium elevation can override the reduction in release probability caused by depletion of PI(3)P but not the defects in SV endocytosis (see Figs 2C and 4A–D). Further studies will be needed to unravel the exact mechanism by which Cdk5 activation in response to PI(3)P depletion represses presynaptic neurotransmission and vesicle cycling.

The neuronal function of VPS34 and its lipid product PI(3)P in the regulation of neurotransmission and SV cycling described here is distinct from the known roles of PI(3)P in non‐neuronal cells such as cell division (Sagona *et al*, 2010), protein sorting, and membrane recycling and turnover (Simonsen *et al*, 1998; Raiborg *et al*, 2013; Ketel *et al*, 2016). The fact that acute inhibition of Cdk5 activity rescues defects in synaptophysin‐pHluorin endocytosis during multiple trains of APs (compare Fig 6B) in the near complete absence of PI(3)P further indicates that PI(3)P‐containing endosomes are unlikely to be absolutely essential for SV recycling in excitatory hippocampal neurons. However, other functions of endosomes in synaptic (e.g., postsynaptic receptor recycling (van der Sluijs & Hoogenraad, 2011)) and/or axonal membrane homeostasis (Jahne *et al*, 2015) or neurotrophin signaling (Yamashita & Kuruvilla, 2016) are not precluded by our data.

Finally, our findings regarding the role of PI(3)P in the control of neurotransmission and SV cycling can explain the involvement of PI 3‐phosphate metabolizing enzymes in autism‐spectrum disorders (Enriquez‐Barreto & Morales, 2016; Krishnan *et al*, 2016), that is, neurodevelopmental diseases characterized by excitatory/inhibitory imbalance (Nelson & Valakh, 2015), and in neurodegeneration (Zhou *et al*, 2010). For example, it is conceivable that loss of PI(3)P observed in the brains of Alzheimer's disease patients (Morel *et al*, 2013) may impact on tau phosphorylation via hyperactivation of Cdk5. Pharmacological targeting of endosomal PI(3)P synthesis and turnover may thus open new avenues for the treatment of these diseases and other neurological disorders associated with imbalanced excitatory/inhibitory neurotransmission or epileptic forms of activity.

## Materials and Methods

### Reagents and Tools table

**Table:** 

Reagent/Resource	Reference or source	Identifier or catalog number
**Recombinant DNA**		
Synaptophysin‐pHluorin	L. Lagnado, University of Sussex, UK	N/A
VGlut1‐pHluorin	S. Voglmaier, UCSF, San Francisco, CA	N/A
mRFP‐FKBP‐hMTM1	T. Balla, NIH, Bethesda, MD	N/A
FRB‐iRFP‐Rab5	This paper	N/A
VPS34‐SNAP	This paper	N/A
VPS34‐SNAP T159A	This paper	N/A
mCherry‐Rab5 S34N (DN)	Addgene	Cat#: 35139
mCherry‐Rab5 Q79L (CA)	Addgene	Cat#: 35140
MISSION^®^ pLKO.1‐puro Non‐Mammalian shRNA Control Plasmid DNA	Sigma‐Aldrich	Cat#: SHC002
pLKO.1‐puro shRNA against mouse PIK3C3 (VPS34)	Sigma‐Aldrich	Cat#: TRCN000025373
pLKO.1‐puro shRNA against mouse CAPN1 (Calpain 1)	Sigma‐Aldrich	Cat#: TRCN0000030664
pLKO.1‐puro shRNA against mouse CAPN2 (Calpain 2)	Sigma‐Aldrich	Cat#: TRCN0000030672
**Antibodies**		
GFP (rabbit polyclonal, used at 1:1,000 in IF)	Abcam	Cat# ab6556, RRID:AB_305564
GFP (mouse monoclonal, used at 1:500 in IF)	Invitrogen	Cat# A11120, RRID: AB_221568
DsRed (rabbit polyclonal, used at 1:300 in IF)	Takara Bio Clontech	Cat# 632496, RRID:AB_10013483
MAP2 (guinea pig polyclonal, used at 1:500 in IF)	Synaptic Systems	Cat# 188 004, RRID:AB_2138181
Synapsin 1/2 (guinea pig polyclonal, used 1:200 in IF)	Synaptic Systems	Cat# 106 004, RRID:AB1106784
Homer1 (mouse monoclonal, used at 1:100 in IF)	Synaptic Systems	Cat# 160 011, RRID:AB_2120992
vGlut1 (guinea pig polyclonal, used at 1:500 in IF)	Synaptic Systems	Cat# 135 304, RRID:AB_887878
PSD95 (mouse monoclonal, used at 1:100 in IF)	Thermo‐Fisher	Cat# MA1‐045, RRID:AB_325399
vGAT (guinea pig polyclonal, used at 1:100 in IF)	Synaptic Systems	Cat# 131 011, RRID:AB_887872
Gephyrin (mouse monoclonal, used at 1:100 in IF)	Synaptic Systems	Cat# 147 111, RRID:AB_887719
vGlut1 (rabbit polyclonal, used at 1:1,000 in IF)	Synaptic Systems	Cat# 135 302, RRID:AB_887877
Synaptophysin (mouse monoclonal, used at 1:500 in IF)	Synaptic Systems	Cat# 101 011, RRID:AB_887824
Synaptotagmin 1 (rabbit polyclonal, used at 1:100 in IF)	Synaptic Systems	Cat# 105 103, RRID:AB_11042457
Calpain 1 (mouse monoclonal, used at 1:100 in IF)	Thermo‐Fisher	Cat# MA3‐940, RRID:AB_2069338
Calpain 2 (rabbit polyclonal, used at 1:100 in IF)	Cell Signaling	Cat# 2539, RRID:AB_2069843
Calpain 2 (mouse polyclonal, used at 1:50 in IF)	Santa Cruz	Cat# SC‐373966, RRID:AB_10917913
Goat anti mouse IgG Alexa Fluor 488 (used at 1:400 in IF)	Thermo‐Fisher	Cat# A‐11001, RRID:AB_2534069
Goat anti rabbit IgG Alexa Fluor 488 (used at 1:400 in IF)	Thermo‐Fisher	Cat# A‐11008, RRID:AB_143165
Goat anti mouse IgG Alexa Fluor 568 (used at 1:400 in IF)	Thermo‐Fisher	Cat# A‐11004, RRID:AB_2534072
Goat anti rabbit IgG Alexa Fluor 568 (used at 1:400 in IF)	Thermo‐Fisher	Cat# A‐11011, RRID:AB_143157
Goat anti mouse IgG Alexa Fluor 647 (used at 1:400 in IF)	Thermo‐Fisher	Cat# A‐21235, RRID:AB_2535804
Goat anti guinea pig IgG Alexa Fluor 568 (used in 1:400)	Thermo‐Fisher	Cat# A‐11075, RRID:AB_141954
Goat anti rabbit Alexa 594 (used at 1:200 in IF)	Thermo‐Fisher	Cat# A‐32740, RRID:AB_2762824
Goat anti mouse Alexa 594 (used at 1:200 in IF)	Thermo‐Fisher	Cat# A‐32744, RRID:AB_2762826
Donkey anti guinea pig Atto647N (used at 1:200 in IF)^†^	Jackson	Cat# 706‐005‐148, RRID:AB_2340443
Donkey anti mouse Atto542 (used at 1:200 in IF)^†^	Jackson	Cat# 715‐005‐151, RRID:AB_2340759
Donkey anti rabbit Atto542 (used at 1:200 in IF)^†^	Jackson	Cat# 711‐005‐152, RRID:AB_2340585
VPS34 (rabbit monoclonal, used at 1:250 in WB)	Cell Signaling	Cat# 4263, RRID:AB_2299765
Synaptotagmin 1 (mouse monoclonal, used at 1:500 in WB)	Synaptic Systems	Cat# 105 011, RRID:AB_887832
Clathrin heavy chain (mouse, used at 1:10 in WB)	Volker Haucke Lab	N/A
Syntaxin 1A (mouse monoclonal, used at 1:500 in WB)	Synaptic Systems	Cat# 110 001, RRID:AB_887843
SNAP25 (mouse monoclonal, used at 1:500 in WB)	Synaptic Systems	Cat# 111 011, RRID:AB_887794
Dynamin 1‐2‐3 (rabbit polyclonal, used at 1:500 in WB)	Synaptic Systems	Cat# 115 002, RRID:AB_887714
Dynamin 1 (rabbit polyclonal, used 1:1,000 in WB)	Pietro De Camilli, Yale University, New Haven, CT	N/A
Dynamin 1 phospho S774 (rabbit polyclonal, used 1:4,000 in WB)	Abcam	Cat# ab55324, RRID:AB_879828
Dynamin 1 phospho S778 (sheep polyclonal, used 1:1,000 in WB)	Rockland/Biomol	Cat#: 612‐601‐D33, RRID:AB_11182908
HSC70 (mouse monoclonal, used at 1:1,000 in WB)	Thermo‐Fisher	Cat# MA3‐006, RRID:AB_325454
PI3KC2α (rabbit polyclonal, used at 1:100 in WB)	Abcam	Cat#: ab154583, RRID:AB_2861168
PI3KC2β (mouse monoclonal, used at 1:100 in WB)	BD transduction	Cat# 611342, RRID:AB_398864
Rab5 (mouse monoclonal, used at 1:200 in WB)	BD transduction	Cat# 610724, RRID:AB_398047
GM130 (mouse monoclonal, used at 1:5,000 in WB)	BD transduction	Cat# 610822, RRID:AB_398141
Cdk5 (mouse monoclonal, used at 1:250 in WB)	Santa Cruz	Cat# sc‐6247, RRID:AB_627241
p35/p25 (rabbit monoclonal, used at 1:250 in WB)	Cell Signaling	Cat# 2680, RRID:AB_1078214
Active capase3 (rabbit polyclonal, used at 1:100 in WB)	Cell Signaling	Cat# 9661, RRID:AB_2341188
Tau (mouse monoclonal, used at 1:1,000 in WB)	Thermo Fisher	Cat# MN1000, RRID:AB_2314654
Tau phospho S199, S202 (rabbit polyclonal, used at 1:500 in WB)	Thermo Fisher	Cat# 44‐768G, RRID:AB_2533749
Tau phospho S202, T205 (mouse monoclonal, used 1:500 in WB)	Thermo Fisher	Cat# MN1020, RRID:AB_223647
β3‐tubulin (rabbit polyclonal, used at 1:10,000 in WB)	Synaptic Systems	Cat# 302 302, RRID:AB_10637424
GAPDH (mouse monoclonal, used at 1:5,000 in WB)	Sigma‐Aldrich	Cat# G8795, RRID:AB_1078991
β‐Actin (mouse monoclonal, used at 1:5,000 in WB)	Sigma‐Aldrich	Cat# A5441, RRID:AB_476744
**Chemicals and other reagents**		
VPS34IN1 (used at 3–10 µM)	MRC PPU Reagents	N/A
SAR405 (used at 20–30 µM)	MRC PPU Reagents	N/A
VPS34 inhibitor 1 (used at 50 µM)	SelleckChem	Cat#: S8456
ALLN (used at 100 µM)	Sigma‐Aldrich	Cat#: A6185
Calpeptin (used at 10 µM)	Sigma‐Aldrich	Cat#: C8999
Roscovitine (used at 10 µM)	Sigma‐Aldrich	Cat#: R7772
Dinaciclib (used at 10 µM)	SelleckChem	Cat#: S2768
Cyclosporin A (used at 10 µM)	Sigma‐Aldrich	Cat#: C3662
Gabazine (used at 10 µM)	Sigma‐Aldrich	Cat#: S106
D‐AP5 (used at 20 µM)	Abcam	Cat#: ab120003
CNQX (used at 10 µM)	Sigma‐Aldrich	Cat#: C127
Picrotoxin (used at 50 µM)	Sigma‐Aldrich	Cat#: P1675
Tetrodotoxin (TTX) (used at 0.2–0.5 µM)	Tocris	Cat#: 1078
EGTA (used at 2 mM)	Sigma‐Aldrich	Cat#: E3889
Rapalog (used at 2 µM)	Takara Bio	Cat#: 635056
SNAP‐ligand JF646 (used at 1 µM)	J. Broichhagen, FMP, Berlin	Poc *et al* (2020)
PI(3)P‐AM (used at 20 µM)	C. Schultz, OHSU, Portland, OR	Subramanian *et al* (2020)

^†^ Unconjugated antibodies were purchased from Jackson and custom labeled with Atto542 or Atto647N by Atto‐Tec.

### Methods and Protocols

#### Animals for neuron cultures and acute slice preparations

All animal experiments were reviewed and approved by the ethics committee of the “Landesamt für Gesundheit und Soziales” (LAGeSo) Berlin and were conducted accordingly to the committee’s guidelines. Mice used for all experiments (C57BL/6 strain) had a normal health and immune status and were regularly checked for standard pathogens. Mice were housed under 12/12‐h light/dark cycle and up to five animals per cage, with access to food and water *ad libitum*.

#### Preparation of primary hippocampal and cerebellar neuron cultures and transfection

Hippocampal neurons were used for transfection, live‐imaging, immunocytochemistry, and electron microscopy. The assessment of synaptic protein levels by immunoblotting after pharmacological treatments were carried out using cerebellar neurons. To prepare neuronal cultures, hippocampus or cerebellum were surgically removed from postnatal mice at p1‐3 or p6, respectively. This was followed by trypsin digestion and dissociation into single neurons. Primary neurons were plated onto PLL‐coated coverslips for 6‐well plates and cultured in MEM medium (Thermo Fisher) containing 2% B27 and 5% FCS. The medium for cerebellar cultures additionally contained 25 mM of KCl. To avoid astrocyte growth, hippocampal cultures were treated with 2 µM of AraC. Neurons were transfected with overexpression or shRNA knockdown plasmids at DIV7‐9 using the calcium phosphate method (Promega). Briefly, 2× HEPES buffer and 6 µg of plasmid DNA were mixed with 250 mM of CaCl_2_ and incubated for 20 min to allow precipitate formation. In parallel, neurons were starved in osmolarity‐adjusted, serum‐free NBA medium (Gibco) at 37°C, 5% CO_2_. Precipitates were added to the neurons and incubated for 30 min at 37°C, 5% CO_2_. Finally, the coverslips were washed twice with osmolarity‐adjusted HBSS (Gibco) and returned to the original neuronal medium.

#### Hippocampal slice preparation and instrumentation for electrophysiological recordings

Acute hippocampal slices were prepared from 2–3 months‐old C57BL/6 mice. Mice were quickly decapitated after cervical dislocation, and brains extracted into ice‐cold dissection artificial cerebrospinal fluid (ACSF) containing: 2.5 mM of KCl, 1.25 mM of NaH_2_PO_4_, 24 mM of NaHCO_3_, 1.5 mM of MgSO_4_, 2 mM of CaCl_2_, 25 mM of glucose, 250 mM of sucrose. 350 µm thick transversal slices (Leica, VT 1200S) were prepared from both hemispheres in oxygenated (95% O_2_/5% CO_2_) dissection ACSF at low temperature (4°C). Hippocampal slices were collected in a chamber (Harvard apparatus, BSC‐PC) filled with resting/ recording ACSF supplemented with 120 mM of NaCl instead of 250 mM of sucrose. Resting solution was continuously oxygenated and kept at room temperature (22–24°C) letting slices to recover for at least 1.5 h before recordings (pH 7.35–7.40). After recovery, slices were transferred to a recording chamber (Warner instruments RC‐27L), filled with oxygenated ACSF at room temperature (22–24°C) with solution exchange of 3–5 ml per min. Slices were fixed to prevent floating and an upright microscope (Olympus, BX61WI) was used for positioning the stimulating and recording electrodes. A glass stimulating electrode (Hilgenberg) filled with ACSF (1–1.5 MΩ) was positioned to stimulate the Schaffer collaterals of the CA1 stratum radiatum. A similar glass electrode (1.5–2.5 MΩ) placed in the distal part of the CA1 region was used for fEPSP and PS recordings. Data were recorded at a sampling rate of 10 kHz, low‐pass filtered at 3 kHz and analyzed using PatchMaster software (Heka Elektronics).

#### fEPSP recordings

The recording electrode was placed 300–500 μm away from the stimulating electrode in *stratum radiatum* for fEPSP recordings and slowly advanced until largest responses were obtained. Stable baseline recordings of 30–50% of the maximal responses were monitored at 0.2 ms stimulus duration and 0.05 Hz frequency for at least 10 min before recordings. Once stabile baseline recordings were obtained, input–output stimulus response curves were recorded as a measure of basal excitatory synaptic transmission. Slopes of the fEPSP were plotted against fiber volley (FV) amplitudes as a function of increasing stimulation intensity. Stimulation intensity was increased by 20 µA steps until the maximal fEPSP were obtained, defined as a response with superimposed population spike (PS) component on decaying fEPSP responses (Appendix Fig S1A). Synaptic transmission was recorded at 0.05 Hz frequency and 50% of maximal stimulation intensity for 1 h to evaluate the effect of different treatments on basal synaptic transmission. As a control treatment 0.03% of DMSO in ACSF was used similar to what was needed to dissolve chemicals. The mean slopes of fEPSPs recorded 10 min before treatment were taken as 100% and effects measured as mean average values between 50 and 60 min (Appendix Fig S1B). Paired‐pulse facilitation as a surrogate measure for release probability was tested by delivering two pulses at time intervals 10, 20, 50, 100, 200, and 500 ms at a stimulation intensity, which induced one third of the maximal responses. PPF was calculated as a percentage increase of the slope of the second response compared to the first (Appendix Fig S1C).

#### Population spike recordings

Output APs from pyramidal cells were measured as a function of the excitability of neuronal networks. For these sets of experiments, the recording electrode was placed ~300 μm away from the stimulating electrode in the CA1 *stratum pyramidale*. Once stable baseline recordings were obtained at a stimulation intensity that yielded about one third of the maximal responses, input–output stimulus response curves from 0 to 300 µA, with 30 µA steps were recorded. The amplitudes of PS and fiber volleys (FV) were plotted as a function of increasing stimulation intensity (Fig EV5A). To probe feedback GABAergic inhibitory modulation of PS amplitudes, we delivered two pulses of stimulation at a supramaximal intensity of 200 µA at different time intervals (from 10 to 500 ms). Paired pulse modulation of the amplitudes of the second to the first response were calculated (as indicated in Fig EV5D) and expressed in %. Each trace measured for the stimulus response curve and paired pulse parameters is an average of three consecutive stimulations delivered every 20 and 30 s for stimulus response curves and paired pulse protocols, respectively, for both fEPSP and PS recordings. To analyze activity‐dependent disinhibition of hippocampal networks, we recorded PSs by delivering 60 pulses at 1 Hz with the same supramaximal intensity of 200 µA. The amplitudes of the PSs and the appearance of polyspikes were analyzed as a measure of activity‐dependent disinhibition of hippocampal networks. Polyspiking induced by the GABAergic antagonist picrotoxin were calculated as the sum area changes of PS and polyspikes using a custom‐written algorithm implemented in R (version 3.6.0) using the “area.between.curves”‐function as indicated in Fig EV5E and F.

#### Live imaging of synaptic vesicle exo‐ and endocytosis via pHluorins

pHluorin‐based assays (e.g., synaptophysin‐pHluorin or vGLUT1‐pHluorin) were used for tracking SV exo‐/ endocytosis. Cultured neurons at DIV12‐16 were pre‐incubated with different pharmacological inhibitors and placed into an RC‐47FSLP stimulation chamber (Warner instruments) for electrical field stimulation using a Digitimer Ltd MultiStim System‐D330 electrical stimulator (100 mA pulses with 1 ms width). Imaging was performed at 37°C in basic imaging buffer (170 mM of NaCl, 3.5 mM of KCl, 20 mM of N‐Tris[hydroxyl‐methyl]‐methyl‐2‐aminoethane‐sulfonic acid (TES), 0.4 mM of KH_2_PO4, 5 mM of glucose, 5 mM of NaHCO_3_, 1.2 mM of MgCl_2_, 1.2 mM of Na_2_SO_4_, 1.3 mM of CaCl_2_, 50 mM of AP5, and 10 mM of CNQX, pH 7.4). To distinguish whether increased surface retention of pHluorin‐tagged SV proteins is due to defective endocytosis or impaired re‐acidification of endocytosed vesicles, acid quench assays were performed by locally perfusing neurons with acidic imaging buffer (where TES was replaced by 2‐(N‐morpholino) ethane‐sulfonic acid, adjusted to pH 5.5) for 20 s before and after electrical stimulation (Fig EV2A). All images were obtained by epifluorescence microscopy (Nikon Eclipse Ti) using an eGFP filter set F36‐526, 40Χ oil‐immersion objective, and a sCMOS camera (Neo, Andor), and analyzed by ImageJ using custom‐written macros (available at https://github.com/DennisVoll/pHluorin_ROI_selector/). The fluorescent changes were normalized to the average fluorescence signal of the first 10 s before electrical stimulation.

For stimulations with multiple trains (e.g., 4 × 200 APs, 40 Hz, 5 s), the surface levels of synaptopHluorin at 90 s after each stimulation was plotted. Surface retention was assessed by normalizing the surface pHluorin levels to baseline fluorescence.

#### Immunocytochemistry of hippocampal neurons in culture

For PI(3)P labeling, DIV12–16 neurons were fixed with 2% PFA for 15 min at RT, washed three times for 5 min with PBS containing 50 mM of NH_4_Cl and permeabilized for 5 min by 20 µM of Digitonin in Buffer A (20 mM of pipes, 137 mM of NaCl and 2.7 mM of KCl, pH 6.8). Excess digitonin was removed by washing neurons in Buffer A. To block the antibody epitopes and to label PI(3)P, neurons were treated for 30 min with Buffer A containing 5% normal goat serum and 0.5 µg/ml of GFP‐2XFYVE probe [purified from bacteria as previously described (Hammond *et al*, 2009)]. To remove the unbound probe, neurons were washed for 5 min with Buffer A, followed by 1 h incubation with primary antibodies against GFP, MAP2 and Synaptophysin (Table EV1) and a 45 min incubation at room temperature with corresponding secondary antibodies coupled to Alexa Flour 488, 568, 647 for confocal imaging and to Alexa 594, Atto542, and Atto647N for STED imaging. Unbound antibodies were removed by washing with Buffer A after each step. Stained neurons were postfixed with 2% PFA for 5 min and mounted on glass slides using Immumount (Thermo Fisher) for confocal imaging and ProLong Gold Antifade Mountant (Thermo Fisher) for STED imaging.

#### Confocal and gSTED imaging of hippocampal neurons

For confocal imaging, neurons were imaged by Zeiss Axiovert 200 M spinning disk confocal microscope equipped with the Perkin‐Elmer Ultra View ERS system and a Hamamatsu C9100 EM‐CCD camera under the control of Volocity software (Perkin‐Elmer). The cumulative fluorescence intensities in a region of interest are calculated using the built‐in analyze particles function of ImageJ (NIH). Where indicated, neurons were stimulated with 4 × 200 APs (40 Hz, 5 s) at 90 s interstimulus train intervals using RC‐47FSLP stimulation chambers (Warner instruments) and a Digitimer Ltd MultiStim System‐D330 electrical stimulator (100 mA pulses with 1 ms width). STED imaging was performed on a Leica SP8 TCS 3× STED microscope (Leica Microsystems) equipped with a pulsed white‐light excitation laser (WLL; ~80 ps pulse width, 80 MHz repetition rate; NKT Photonics) and a STED laser for depletion at 775 nm. DMSO and VPS34IN1‐treated neurons were acquired with equal settings. Three‐channel STED imaging was performed by sequentially imaging at 540 nm excitation/550–600‐nm emission for Atto542; 594 nm excitation/604–650‐nm emission for Alexa594; and 646‐nm excitation, 656–750‐nm emission for Atto647N. The 775‐nm STED laser was used to deplete all fluorophores. Fluorescence signals were detected sequentially by hybrid detectors at appropriate spectral regions separated from the STED laser. Images were acquired with an HC PL APO CS2 100×1.40 N.A. oil objective (Leica Microsystems), a scanning format of 1,024 × 1,024 pixels and six‐fold zoom, resulting in 18.9‐nm pixel size. Raw data obtained from three‐channel time gated STED (gSTED) imaging were analyzed with ImageJ (NIH). Synapses, where a clear apposition between presynaptic and postsynaptic clusters that appeared parallel to the focal plane, were selected for image representation. To determine the localization of the synaptic PI(3)P clusters, multicolor line profiles were generated perpendicular to the Synapsin and Homer 1 cocluster with a length of 1 µm.

#### Electron microscopy and tomography

DIV14 neurons were treated with 0.1% DMSO or 10 µM VPS34IN1 for 1 h and electrically stimulated with 4 × 200 APs (40 Hz, 5 s) at 90 s interstimulus train intervals using RC‐47FSLP stimulation chambers (Warmer instruments) and a Digitimer Ltd MultiStim System‐D330 electrical stimulator (100 mA pulses with 1 ms width). The stimulation chambers were disassembled and 20 s after the end of the stimulation the coverslips were immersed into PBS with 2% glutaraldehyde. The coverslips were then postfixed with 1% OsO_4_ and 1.5% potassium hexacyanoferrat (III) and stained *en bloc* with 1% uranyl acetate, followed by dehydration in a methanol gradient, propylene oxide, and Epoxy resin infiltration. After polymerization, coverslips were removed and 50‐nm sections were cut and contrasted with uranyl acetate and lead citrate. Images were obtained using a Zeiss 900 transmission electron microscope (TEM). The density of synaptic vesicles (SVs), clathrin‐coated vesicles (CCVs), clathrin‐coated pits (CCPs), endosome‐like vacuoles, and noncoated invaginations were measured from synaptic profiles with a clearly visible active zone and adjacent SVs. For TEM tomography, 250‐nm sections were cut and collected on coated slotted grids with 10‐nm gold fiducials. Series of images from +60° to −60° were taken with a 1° step at Tecnai G20 microscope. Etomo/IMOD https://bio3d.colorado.edu/imod/ (Kremer *et al*, 1996) and Microscopy imaging browser MIB http://mib.helsinki.fi/index.html were used to work with 3D volumes and render 3D models of subsynaptic structures (Belevich *et al*, 2016).

#### Immunoblotting and quantification of protein content

Cerebellar granule neurons at DIV7 were treated with different inhibitors for 1 h, and washed 2× with ice‐cold PBS. Cell lysates were harvested in sample lysis buffer (20 mM of Tris pH 7.4, 1% SDS, 5% glycerol, 5 mM of β‐mercaptoethanol, 0.01% bromophenol blue, 0.3% protease inhibitor cocktail (Sigma‐Aldrich), and phosphatase inhibitors (cocktails 2 and 3, Sigma‐Aldrich). Equal protein amounts of lysates were analyzed by SDS‐PAGE and immunoblotting. Primary antibodies against various synaptic proteins were used in immunoblotting, followed by LI‐COR 800CW and 680RD as secondary antibodies. Immunoblots were imaged by LI‐COR‐Odyssey FC detection with Image Studio Lite Version 4.0.

#### Brain fractionation

Fifteen to twenty brains from 8‐ to 10‐weeks‐old C57 mice were collected and homogenized in ice‐cold sucrose buffer (320 mM of sucrose, 4 mM of HEPES, pH 7.4). Homogenate fraction (H) was taken and the rest of the samples were centrifuged at 800 *g* for 10 min. Pellets were resuspended in lysis buffer (10% sucrose, 4 mM of HEPES, 1 mM of PMSF, and protease inhibitor cocktail) as P1 fraction. The supernatant (S1) was further centrifuged at 9,200 *g* for 15 min. Supernatant was collected again (S2) and pellets (P2) were washed by sucrose buffer. The washed pellets were centrifuged at 10,200 *g* for 15 min. P2’ pellets were resuspended in sucrose buffer and further homogenized. Homogenized samples were initially centrifuged at 25,000 *g* for 20 min to obtain pellets (LP1), and the supernatant was further centrifuged at 165,000 *g* for 2 h. The pellets were collected and suspended through 27 G needle with lysis buffer as LP2 fraction as the crude SV fraction. All steps were performed at 4°C.

#### Statistics and reproducibility

All data are presented as mean ± SEM and were obtained from multiple independent experiments (e.g., independent animals or mouse cultures), with total sample numbers provided in the figure legends as N (biological replicates) or n (technical replicates). No statistical method was used to predetermine sample size as sample sizes were not chosen based on prespecified effect size. Instead, multiple independent experiments were carried out using several sample replicates as detailed in the figure legends. No blinding strategy was applied during experimentation. In the experiments that involve light or electron microscopy, the statistical significance between two groups for all normally distributed data (e.g., effects of chemical inhibitors or genetic knockdowns on pHluorin surface retention levels, relative PI(3)P intensity) was evaluated with a two‐tailed Student’s *t*‐test. The statistical significance between more than two groups for all normally distributed data (e.g., percentage of pHluorin surface retention where inhibitor‐treatment, protein overexpression, and gene knockdown constructs are combined) was determined by a two‐way ANOVA using GraphPad6 (Prism) and Sidak’s Multiple Comparison Test (when two groups are compared) or Tukey’s Multiple Comparison Test (when more than two groups are compared) were applied for significance determination between different groups. SigmaPlot (Systat Software Inc.) and IGOR Pro (WaveMetrics, Inc.) were used for electrophysiological data analyses and presentation. For PPF measurements, significance was evaluated using Two Way RM ANOVA. For PS and polyspike amplitude and area measurements significance was evaluated using One Way ANOVA or Student’s *t*‐test. The level of significance is indicated in the figures by asterisks (*P < 0.05; **P < 0.01; ***P < 0.001; ****P < 0.0001). The exact mean ± SEM values for each figure panel, as well as the sample size, the exact statistical test for comparison and significance determination, p, t (or q), and df values for each experiment are provided in the Source Data file.

## Author contributions

**Volker Haucke:** Conceptualization; Resources; Supervision; Funding acquisition; Writing—original draft; Project administration; Writing—review & editing. **Guan‐Ting Liu:** Investigation; Methodology. **Gaga Kochlamazashvili:** Data curation; Formal analysis; Investigation; Methodology. **Dmytro Puchkov:** Conceptualization; Data curation; Investigation; Methodology. **Rainer Mueller:** Resources; Methodology. **Carsten Schultz:** Supervision; Funding acquisition; Methodology. **Albert I Mackintosh:** Investigation. **Dennis Vollweiter:** Investigation; Methodology. **Tolga Soykan:** Conceptualization; Supervision; Investigation; Methodology; Writing—original draft; Writing—review & editing.

In addition to the CRediT author contributions listed above, the contributions in detail are:

G‐TL and TS conducted pHluorin imaging experiments, GK performed all electrophysiology experiments with help in data analysis from DV, DP performed electron microscopy analysis, AIM performed brain fractionations, CS and RM contributed synthetic membrane‐permeant PI(3)P. VH supervised the study and together with TS wrote the paper with input from all authors.

## Disclosure and competing interests statement

The authors have no relevant financial or nonfinancial interests to disclose and declare no competing interests.

## Supporting information



Appendix

Expanded View Figures PDF

Source Data for Expanded View and Appendix

Source Data for Figure 1

Source Data for Figure 2

Source Data for Figure 3

Source Data for Figure 4

Source Data for Figure 5

Source Data for Figure 6

Source Data for Figure 7

## Data Availability

Requests for materials should be addressed to V.H. (haucke@fmp-berlin.de). Source data are available online. All other data are contained in the main manuscript, expanded view, and Appendix. This study includes no data deposited in external repositories.
