# Supplementary material for: Endosomal phosphatidylinositol 3‐phosphate controls synaptic vesicle cycling and neurotransmission
Source: EMBO J. 2022 Mar 22;41(9):e109352. doi: 10.15252/embj.2021109352 (PMC9058544; doi:10.15252/embj.2021109352)
Supplement: Supplementary file 1 — Appendix [file EMBJ-41-e109352-s011.pdf]

Appendix

for

Endosomal phosphatidylinositol 3-phosphate levels control presynaptic vesicle cycling and neurotransmission

Guan-Ting Liu, Gaga Kochlamazashvili, Dmytro Puchkov, Rainer Müller, Carsten Schultz, Albert I. Mackintosh, Dennis Vollweiter, Volker Haucke*, Tolga Soykan*

Correspondence to: soykan@fmp-berlin.de (T.S.) or haucke@fmp-berlin.de (V.H.)

This file includes:

Appendix Figure S1

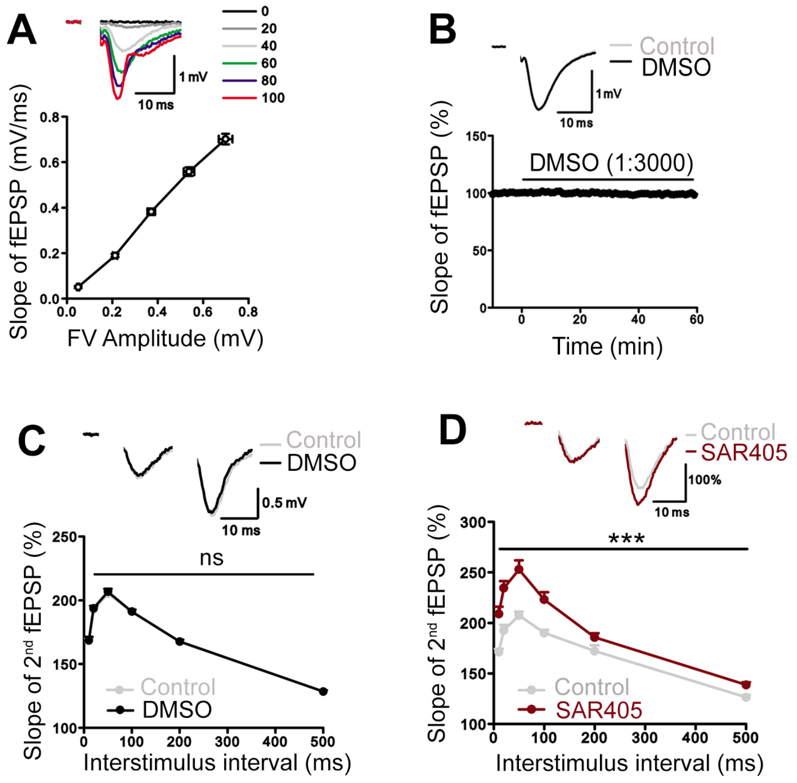

Appendix Figure S1. Pharmacological depletion of PI(3)P synthesis via VPS34 increases basal synaptic transmission.

A Input/output relationships between presynaptic fiber volleys and slopes of fEPSPs show normal basal excitatory synaptic transmission in slices from C57BL/6J mice. Insert samples show fEPSPs representing responses evoked by increasing stimulation strength (from 0 to 100 µA with a 20 µA step). A total of 42 slices from 22 animals.

B Basal synaptic transmission of CA3-CA1 connections in the acute hippocampal slices show no changes after DMSO (0.03%) treatment. Insert samples show the average of 30 consecutive fEPSPs before (-10 to 0 min) and after DMSO treatment (50 to 60 min). The mean slopes of fEPSPs recorded 10 min before treatment was taken as 100%. Mean ± SEM; 18 slices from 18 animals.

C Measurements of paired-pulse facilitation (PPF) with different interstimulus intervals (10 to 500 ms) show no change in the slope of the second fEPSP following DMSO application (0.03%). Mean ± SEM; 18 slices from 18 animals; n.s not significant; Two-way RM ANOVA.

D Measurements of PPF with different interstimulus intervals in CA3-CA1 connections of acute slices treated with SAR405 (20 µM). Representative fEPSP traces with 20 ms intervals before and after application of SAR405 (above) and the relative slope of 2nd fEPSP with different interstimulus intervals (below) are shown. SAR405 application leads to a significant facilitation of the second fEPSP, indicating a lower initial release probability. Mean ± SEM; 6 slices per condition from 4 animals; ***$p < 0.001$; Two-way ANOVA.
