# Supplementary material for: Endosomal phosphatidylinositol 3‐phosphate controls synaptic vesicle cycling and neurotransmission
Source: EMBO J. 2022 Mar 22;41(9):e109352. doi: 10.15252/embj.2021109352 (PMC9058544; doi:10.15252/embj.2021109352)
Supplement: Supplementary file 2 — Expanded View Figures PDF [file EMBJ-41-e109352-s004.pdf]

Expanded View Figures

Figure EV1. PI(3)P is localized at neuronal soma and synapses and endosomal PI(3)P levels are regulated by neuronal activity.

A Subcellular fractionation of mouse brains followed by western blot reveals the presence of Class II and Class III PI3Ks in the synaptic vesicle enriched LP2 fraction.

B Representative 2-color confocal images of cultured hippocampal neurons treated with DMSO (0.1%) or VPS34IN1 (10 μM) for 1 h, fixed and stained for MAP2, synaptophysin (Syp), and PI(3)P. Arrows indicate presynaptic terminals that contain (DMSO) or lack (VPS34IN1) PI(3)P. Scale bar, 10 μm.

C, D Representative 3-color STED images of synapses of cultured hippocampal neurons treated with DMSO (0.1%) or VPS34IN1 (10 μM) for 1 h, fixed and stained for PI (3)P and VGlut1-PSD95 pair (markers for excitatory synapses) in C and VGAT-Gephyrin pair (markers for inhibitory synapses) in D. Scale bar, 0.5 μm. Both excitatory and inhibitory synaptic terminals contain PI(3)P-positive structures, which disappear upon VPS34 inhibition.

E Cultured hippocampal neurons treated with Gabazine (10 μM) overnight, fixed and stained for MAP2, Syp and PI(3)P. Scale bar, 10 μm.

F Cultured hippocampal neurons were treated with Roscovitine (10 μM) for 1 h and field-stimulated with four trains of 200 APs (40 Hz, 5 s; 90 s gap between each train) (Stim) or left unstimulated (–), fixed and stained for MAP2, Syp, and PI(3)P. Scale bar, 10 μm.

G Cultured hippocampal neurons were treated with Dinaciclib (10 μM) for 1 h and field-stimulated with four trains of 200 APs (40 Hz, 5 s; 90 s gap between each train) (Stim) or left unstimulated (–), fixed and stained for MAP2, Syp, and PI(3)P. Scale bar, 10 μm.

H Relative intensity of PI(3)P in neuronal somata ($MAP2^+$) or synapses (Syp^+) of cultured hippocampal neurons were treated with Dinaciclib (10 μM) for 1 h and field-stimulated with four trains of 200 APs (40 Hz, 5 s; 90 s gap between each train) (Stim) or left unstimulated (–). $N = 3$ independent experiments (≥ 15 images per condition); Mean ± SEM; $*P < 0.05$; Two-way ANOVA.

I Cultured hippocampal neurons transfected with VPS34-SNAP or $VPS34_{T159A}$-SNAP at DIV7, treated with Gabazine (10 μM) overnight at DIV13, treated 1 h with SNAP-tag ligand JF646 to label VPS34-SNAP at DIV14 and subsequently fixed and stained for PI(3)P. Scale bar, 10 μm.

J Relative intensity of PI(3)P in neuronal somata of cultured hippocampal neurons expressing VPS34-SNAP or $VPS34_{T159A}$-SNAP, treated with DMSO or Gabazine (10 μM) overnight. $N = 6$ independent experiments (≥ 30 images per condition); Mean ± SEM; $*P < 0.05$; Two-way ANOVA.

Source data are available online for this figure.

 © 2022 The Authors

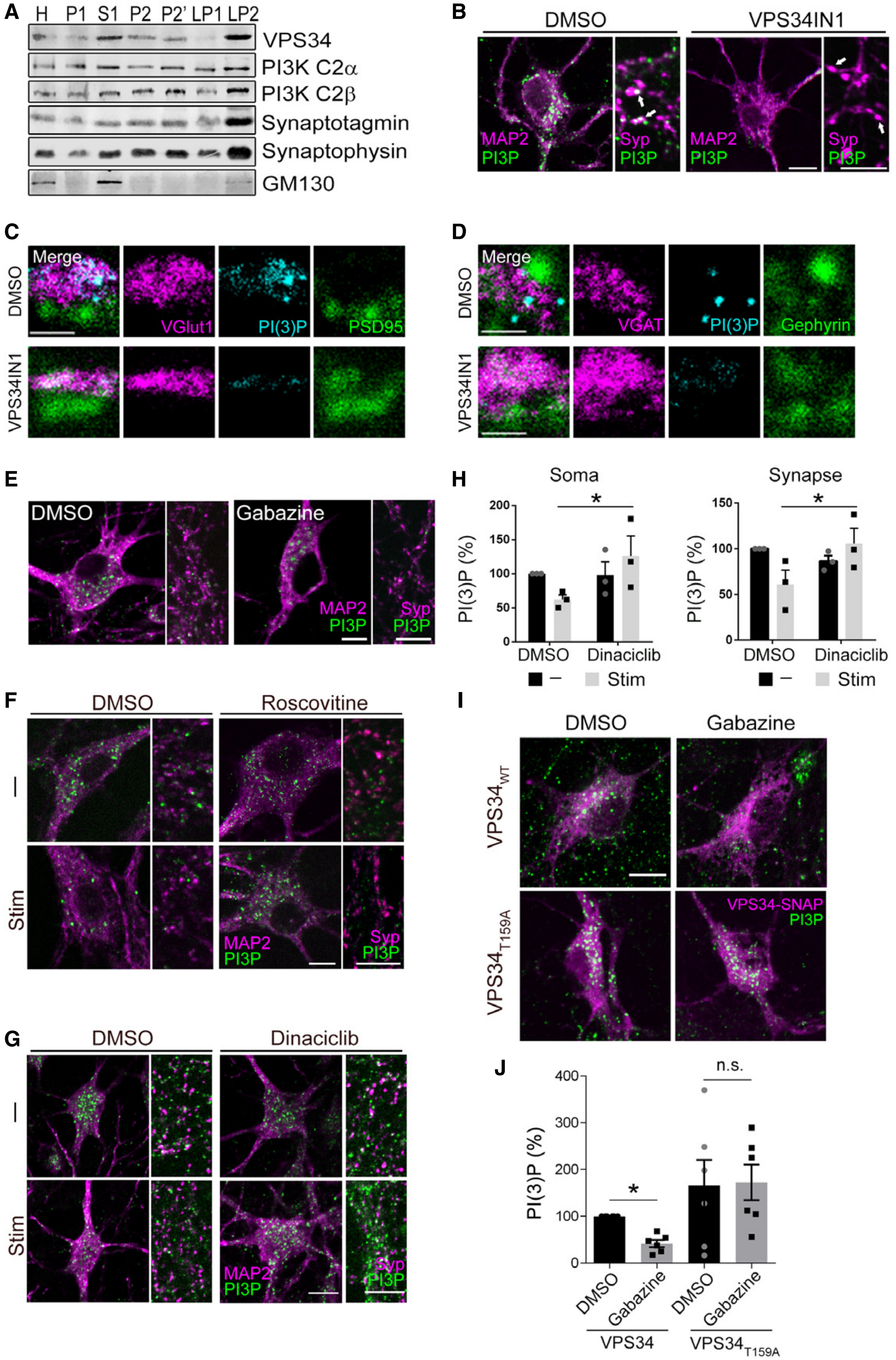
A
H P1 S1 P2 P2' LP1 LP2
VPS34
PI3K C2α
PI3K C2β
Synaptotagmin
Synaptophysin
GM130
B
DMSO
VPS34IN1
MAP2
PI3P
Syp
PI3P
C
Merge
DMSO
VPS34IN1
VGlut1
PI(3)P
PSD95
D
Merge
DMSO
VPS34IN1
VGAT
PI(3)P
Gephyrin
E
DMSO
Gabazine
MAP2
PI3P
Syp
PI3P
H
Soma
Synapse
PI(3)P (%)
DMSO
Dinaciclib
Stim
F
DMSO
Roscovitine
Stim
G
DMSO
Dinaciclib
Stim
I
DMSO
Gabazine
VPS34WT
VPS34T159A
VPS34-SNAP
PI3P
J
n.s.
PI(3)P (%)
DMSO
Gabazine
VPS34
VPS34T159A

Figure EV1.

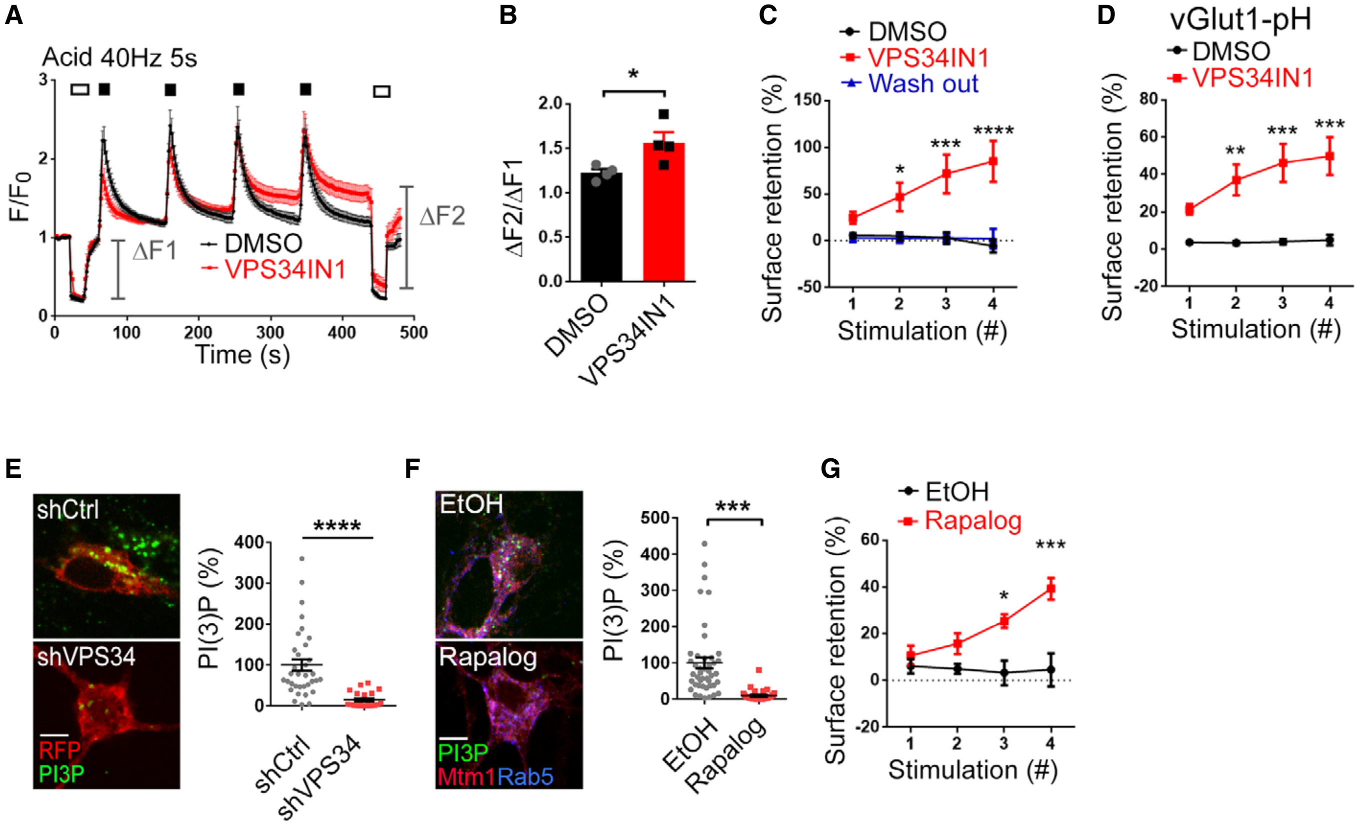

Figure EV2. PI(3)P depletion inhibits SV endocytosis monitored by Synaptophysin-pHluorin.

A Normalized Synaptophysin-pHluorin responses in cultured hippocampal neurons stimulated with four trains of 200 APs (40 Hz, 5 s) with 90 s intervals after 1 h pretreatment with DMSO or VPS34IN1 (10 μM) and subjected to low pH image buffer (Acid) before and after the stimulation trains.

B The ratio of surface fluorescence of Synaptophysin-pHluorin before and after 4 × 200 APs stimulation in DMSO- and VPS34IN1-treated neurons. Mean ± SEM; 15 images per condition from 4 independent experiments; $*P < 0.05$; Student's *t*-test.

C Surface retention of Synaptophysin-pHluorin 90 s poststimulus is plotted for each of the four successive 200 AP stimulation trains in neurons treated with VPS34IN1 (10 μM, 1 h) and washed overnight with conditioned medium. Mean ± SEM; ≥ 11 images per condition from 3 independent experiments; $*P < 0.05$; $***P < 0.001$; $****P < 0.0001$; Two-way ANOVA.

D Surface retention of vGlut1-pHluorin 90 s poststimulus plotted for each 200 AP stimulation (40 Hz, 5 s) in neurons treated with VPS34IN1 (10 μM, 1 h). Mean ± SEM; ≥ 15 images per condition from 3 independent experiments; $**P < 0.01$; $***P < 0.001$; Two-way ANOVA.

E Cultured hippocampal neurons transfected with mRFP and control shRNA or shRNA against VPS34, stained for PI(3)P. Scale bar, 10 μm. Mean ± SEM; $n = 34$ (shCtrl) and $n = 21$ (shVPS34); $****P < 0.0001$; Student's *t*-test.

F Confocal images of cultured hippocampal neurons transfected with mRFP-FKBP-hMTM1 and FRB*-iRFP-Rab5, treated with EtOH or Rapalog and stained for PI(3)P. Scale bar, 10 μm. Mean ± SEM; ≥ 35 images per condition; $***P < 0.001$; Student's *t*-test.

G Surface retention of Synaptophysin-pHluorin 90 s poststimulus is plotted for each of the four successive 200 AP stimulation trains in hippocampal neurons expressing mRFP-FKBP-hMTM1 and FRB*-iRFP-Rab5 and treated with EtOH or Rapalog. Mean ± SEM; ≥ 13 images per condition from 3 independent experiments; $*P < 0.05$; $***P < 0.001$; Two-way ANOVA.

Source data are available online for this figure.

 © 2022 The Authors

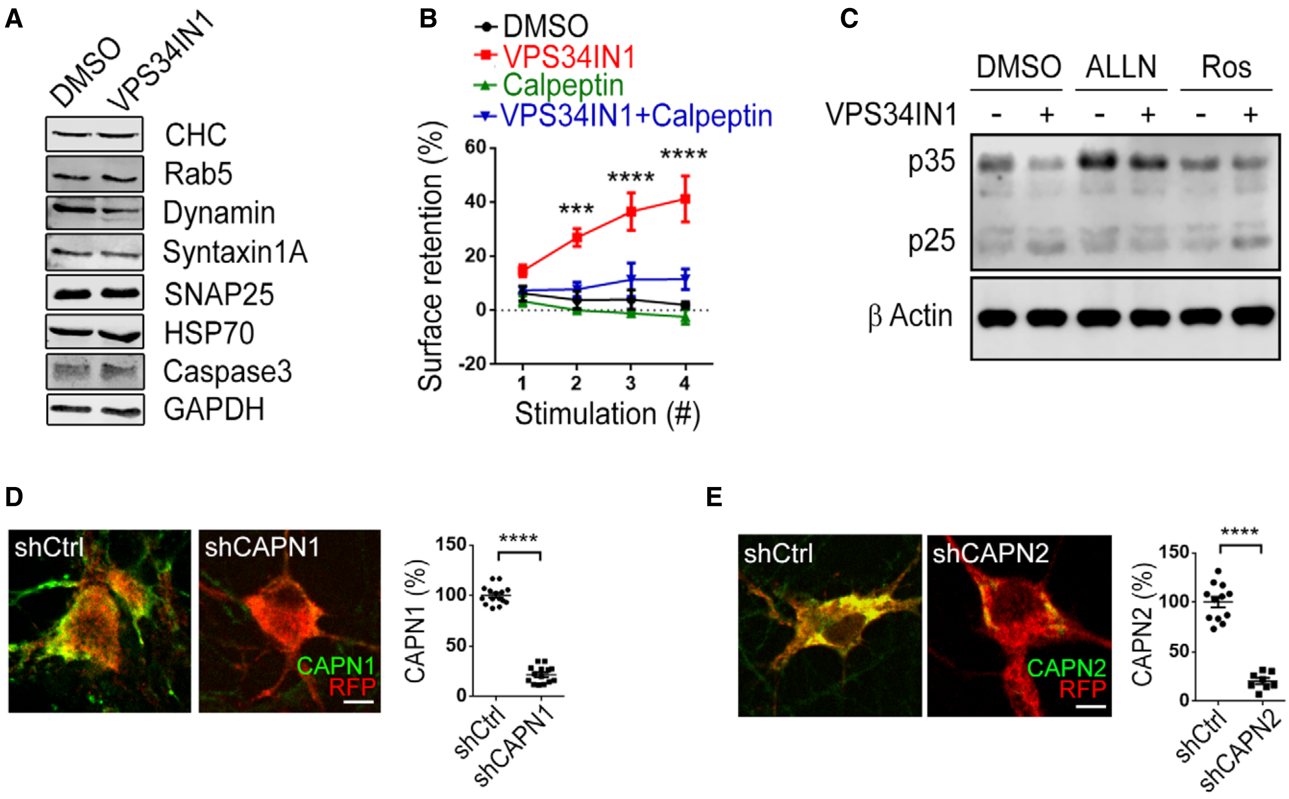

Figure EV3. PI(3)P loss inhibits SV endocytosis via calpain 2 activation.

A Levels of various synaptic proteins in DMSO (0.1%) and VPS34IN1 (10 µM) treated cultured cerebellar granule neurons analyzed by western blot.

B Surface retention of Synaptophysin-pHluorin 90 s poststimulus is plotted for each of the four successive stimulation trains in neurons treated with VPS34IN1 (10 µM) and Calpeptin (10 µM). Mean ± SEM; ≥ 9 images per condition from 3 independent experiments; $^{***}P < 0.001$; $^{****}P < 0.0001$; Two-way ANOVA.

C Processing of p35 to p25 in cultured hippocampal neurons treated with VPS34IN1 (10 µM) for 1 h, analyzed by western blot. Cleavage of p35 to p25 was reduced in neurons simultaneously treated with ALLN (100 µM) but not with Roscovitine (Ros, 10 µM).

D, E Cultured hippocampal neurons were cotransfected with an mRFP expression plasmid and shCtrl, shCAPN1, or shCAPN2 and stained for Calpain 1 in D or Calpain 2 in E. Scale bar, 10 µm. The relative mean fluorescence intensity is plotted. ≥ 8 images per condition; $^{****}P < 0.0001$; Student's *t*-test.

Source data are available online for this figure.

© 2022 The Authors

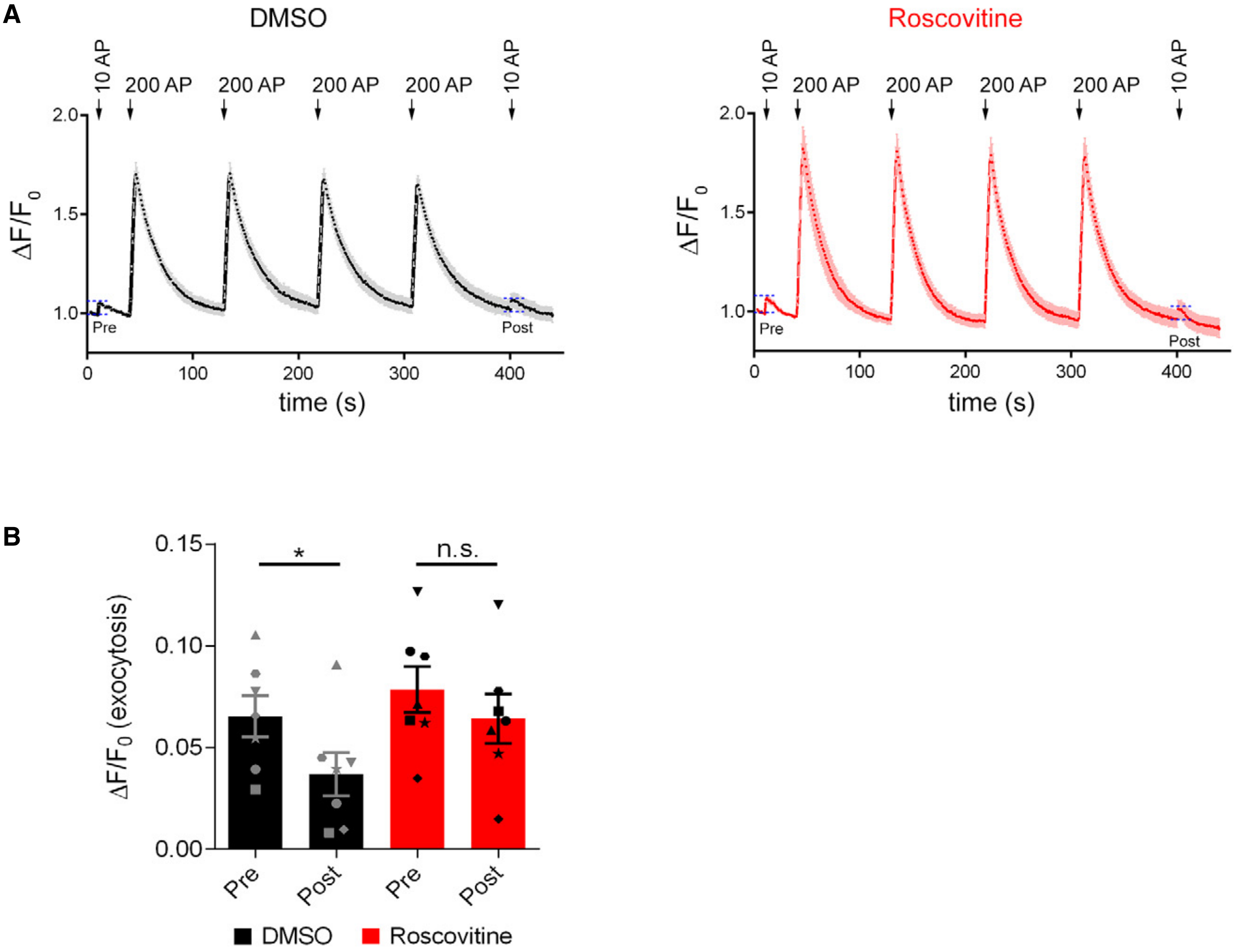

Figure EV4. Repetitive high frequency stimulation reduces the responsiveness of synapses to mild physiological stimulation in a Cdk5-dependent manner.

A Normalized Synaptophysin-pHluorin responses in cultured hippocampal neurons treated with DMSO (0.1%, left) or Roscovitine (10 μM; right) stimulated with 10 APs (40 Hz, 0.25 s), 4 × 200 APs (40 Hz, 5 s), and 10 APs (40 Hz, 0.25 s). The 10 AP stimulations before and after the train of 200 AP stimulations were labeled pre and post, respectively.

B The amplitude of SV release detected by measuring the increase in Synaptophysin-pHluorin fluorescence in response to 10 AP before (pre) and after (post) high frequency stimulation (4 × 200 APs) as indicated by dashed lines in A. Repetitive high frequency stimulation reduces SV release, an effect occluded by Cdk5 inhibition. Mean ± SEM; $N = 7$ independent experiments; $^{*}P < 0.05$; One-way ANOVA; Tukey's Multiple Comparison Test.

Source data are available online for this figure.

 © 2022 The Authors

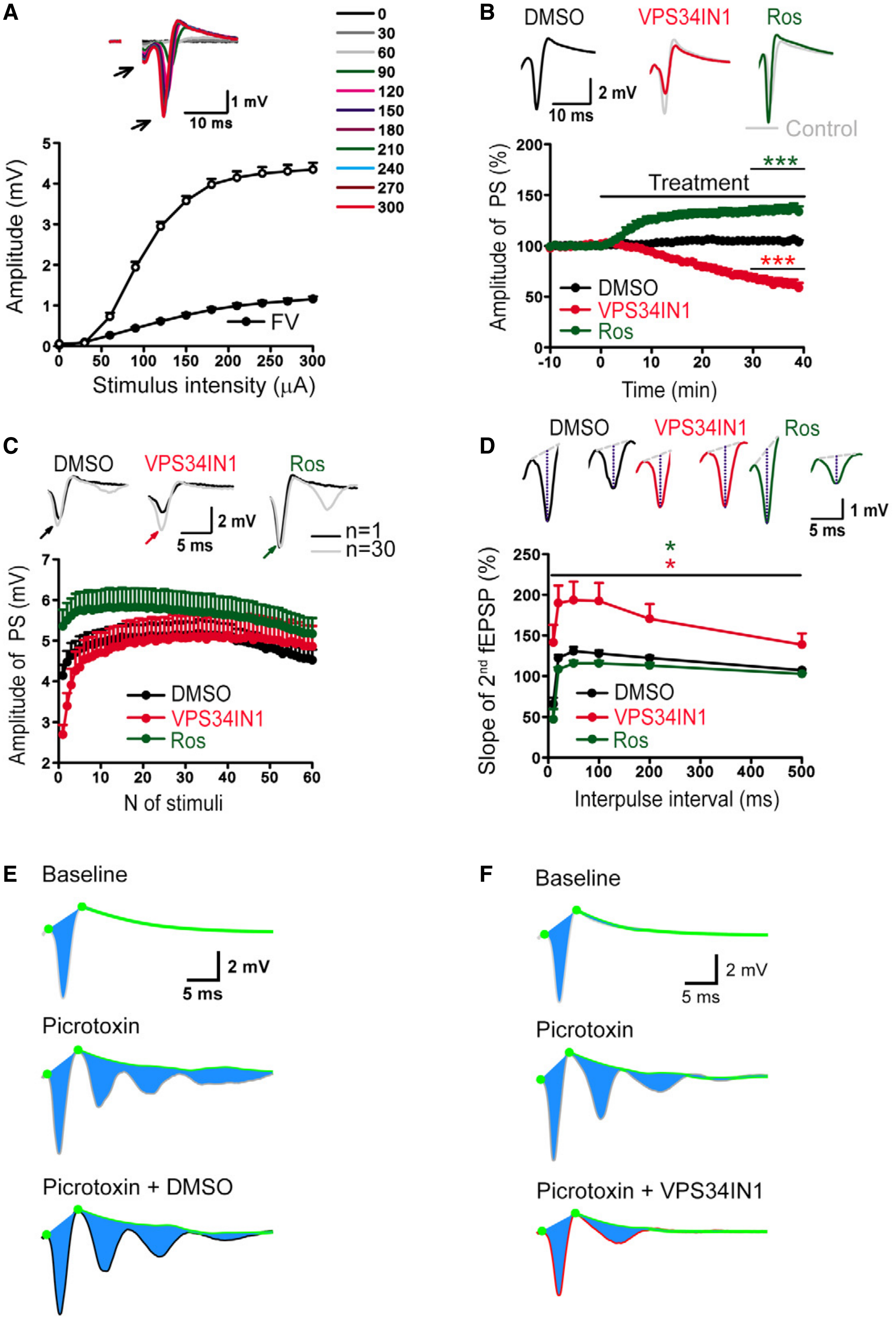
A
0
30
60
90
120
150
180
210
240
270
300
1 mV
10 ms
Amplitude (mV)
FV
Stimulus intensity (μA)
B
DMSO
VPS34IN1
Ros
2 mV
10 ms
Control
Amplitude of PS (%)
Treatment
***
***
Time (min)
C
DMSO
VPS34IN1
Ros
2 mV
5 ms
n=1
n=30
Amplitude of PS (mV)
N of stimuli
D
DMSO
VPS34IN1
Ros
1 mV
5 ms
Slope of 2nd fEPSP (%)
Interpulse interval (ms)
E
Baseline
2 mV
5 ms
Picrotoxin
Picrotoxin + DMSO
F
Baseline
2 mV
5 ms
Picrotoxin
Picrotoxin + VPS34IN1

Figure EV5.

◀ **Figure EV5. Effects of pharmacological inhibition of VPS34 and Cdk5 on hippocampal network activity.**

A Input/output relationships between stimulus intensities and amplitudes of population spikes (PS) or fiber volleys (FV) show normal basal excitability of neuronal populations in CA1 *stratum pyramidale*. Insert sample (above) shows representative PS responses evoked by increasing stimulation strength (from 0 to 300 μA with a 30 μA step). Mean ± SEM; 36 slices from 12 mice.

B Recordings of PS over time did not show significant changes in PS amplitudes in the presence of DMSO (0.03%). PS amplitude is reduced in the presence of VPS34IN1 (3 μM) and facilitated in the presence of Roscovitine (10 μM). Insert samples (above) show the average of 30 subsequent PSs before (from −10 to 0 min) and after (from 30 to 40 min) pharmacological treatments. Mean ± SEM; six slices per condition from six animals;***$P < 0.001$; One-way ANOVA.

C Measurement of activity-dependent facilitation of PSs. DMSO (0.03%) does not affect the facilitation of PS amplitudes during 1 Hz stimulation. VPS34IN1 treatment led to stronger facilitation of PS amplitudes, whereas facilitation was less pronounced in the presence of Roscovitine. Note that under all conditions 1 Hz stimulation eventually increased PS amplitudes to nearly identical maximal levels. Top panels show representative traces N1 (i.e., 1st stimulus response) and N30 (i.e., 30th stimulus response) with PS peaks indicated by color-coded arrows. Mean ± SEM; 12 slices per condition from 12 animals.

D Recordings of paired-pulse modulation of PS at different interpulse intervals (from 10 to 500 ms). VPS34IN1 led to an elevated paired-pulse ratio of PSs compared to DMSO, whereas Roscovitine reduced it. Mean ± SEM; 12 slices per condition from 12 animals; *$P < 0.05$; Two-way ANOVA.

E, F Representative traces show baseline averages of 30 traces before treatment (−10 to 0 min, top), after picrotoxin (50 μM) application (20–30 min, middle) and following treatment with DMSO or VPS34IN1 (60–70 min, bottom).

Source data are available online for this figure.

© 2022 The Authors
