# Supplementary figures and images for: Endosomal phosphatidylinositol 3‐phosphate controls synaptic vesicle cycling and neurotransmission
Source: EMBO J. 2022 Mar 22;41(9):e109352. doi: 10.15252/embj.2021109352 (PMC9058544; doi:10.15252/embj.2021109352)

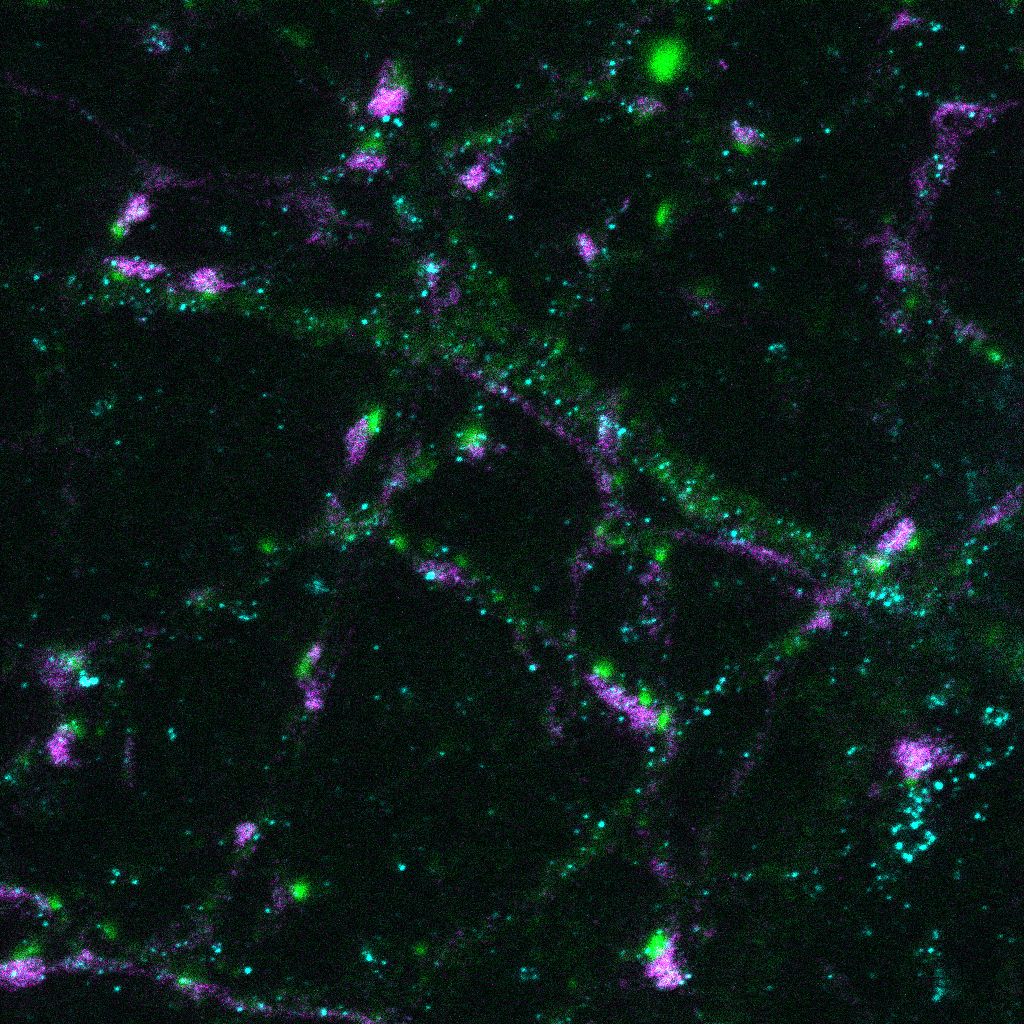

Supplement: Supplementary file 4 — Source Data for Figure 1 [file EMBJ-41-e109352-s009.zip › Figure_1_Source_Data+Statistics/Figure_1A/Figure_1A_Full_image.tif]

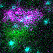

Supplement: Supplementary file 4 — Source Data for Figure 1 [file EMBJ-41-e109352-s009.zip › Figure_1_Source_Data+Statistics/Figure_1A/Figure_1A_Synapse1_merged.tif]

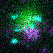

Supplement: Supplementary file 4 — Source Data for Figure 1 [file EMBJ-41-e109352-s009.zip › Figure_1_Source_Data+Statistics/Figure_1A/Figure_1A_Synapse2_merged.tif]

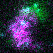

Supplement: Supplementary file 4 — Source Data for Figure 1 [file EMBJ-41-e109352-s009.zip › Figure_1_Source_Data+Statistics/Figure_1A/Figure_1A_Synapse3_merged.tif]

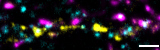

Supplement: Supplementary file 4 — Source Data for Figure 1 [file EMBJ-41-e109352-s009.zip › Figure_1_Source_Data+Statistics/Figure_1C-D/Figure_1C_Crop_Merge.png]

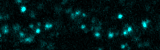

Supplement: Supplementary file 4 — Source Data for Figure 1 [file EMBJ-41-e109352-s009.zip › Figure_1_Source_Data+Statistics/Figure_1C-D/Figure_1C_Crop_PI3P.png]

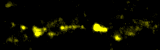

Supplement: Supplementary file 4 — Source Data for Figure 1 [file EMBJ-41-e109352-s009.zip › Figure_1_Source_Data+Statistics/Figure_1C-D/Figure_1C_Crop_VGAT.png]

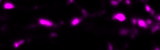

Supplement: Supplementary file 4 — Source Data for Figure 1 [file EMBJ-41-e109352-s009.zip › Figure_1_Source_Data+Statistics/Figure_1C-D/Figure_1C_Crop_VGlut1.png]

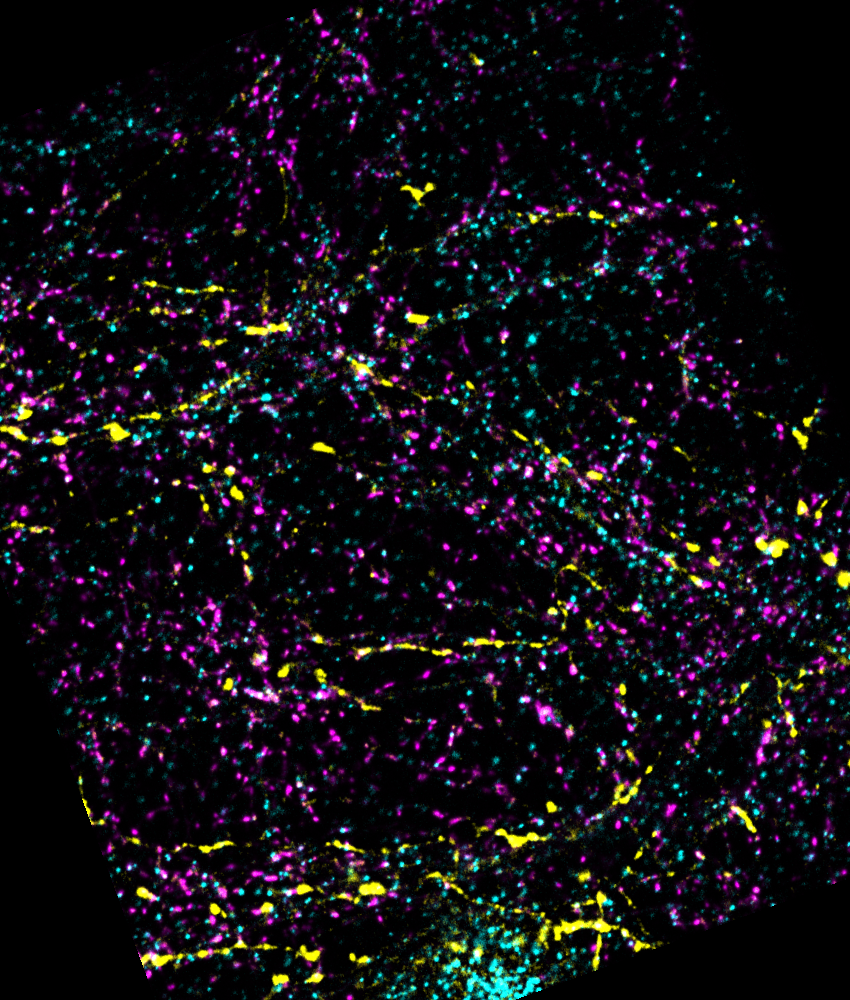

Supplement: Supplementary file 4 — Source Data for Figure 1 [file EMBJ-41-e109352-s009.zip › Figure_1_Source_Data+Statistics/Figure_1C-D/Figure_1C_Full_Image.tif]

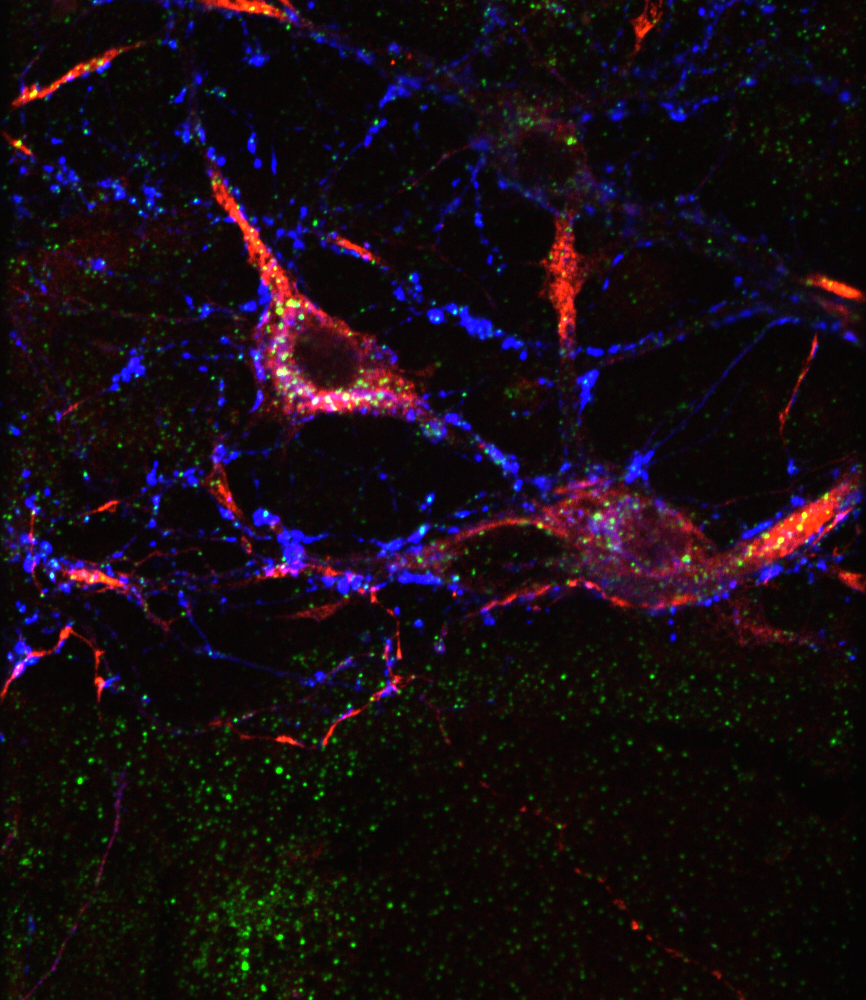

Supplement: Supplementary file 4 — Source Data for Figure 1 [file EMBJ-41-e109352-s009.zip › Figure_1_Source_Data+Statistics/Figure_1F-G/Figure_1F_Full_Image_nonStim.tif]

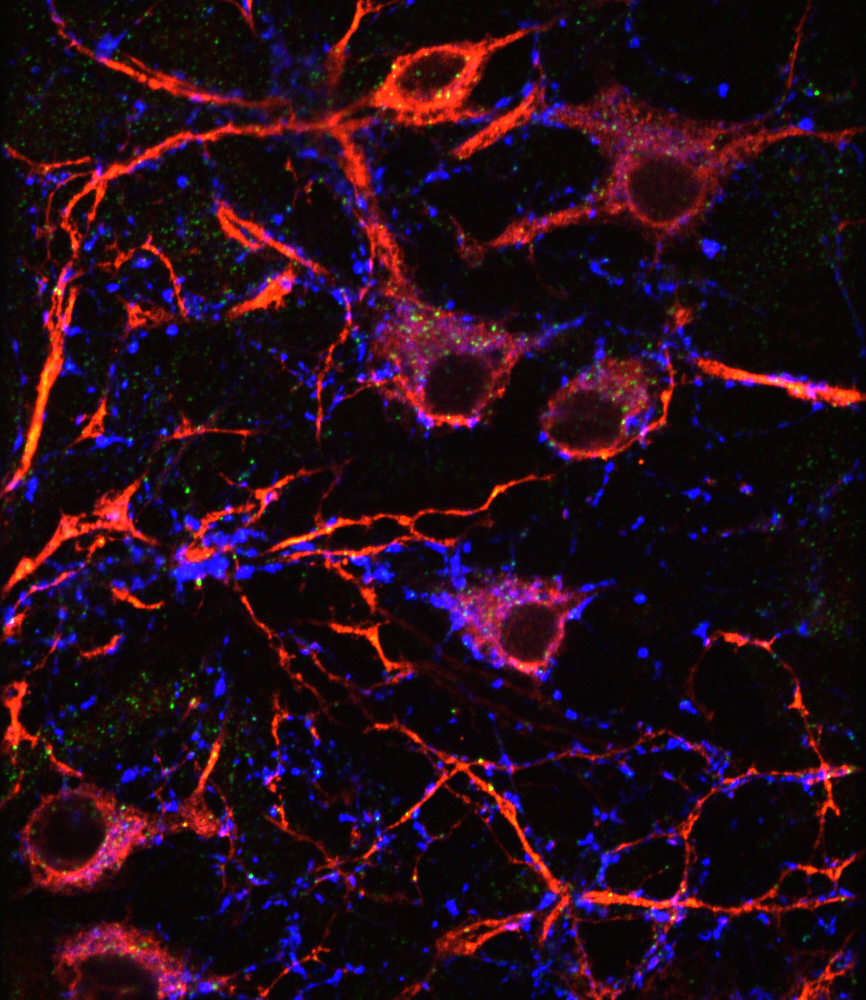

Supplement: Supplementary file 4 — Source Data for Figure 1 [file EMBJ-41-e109352-s009.zip › Figure_1_Source_Data+Statistics/Figure_1F-G/Figure_1F_Full_Image_Stim.tif]

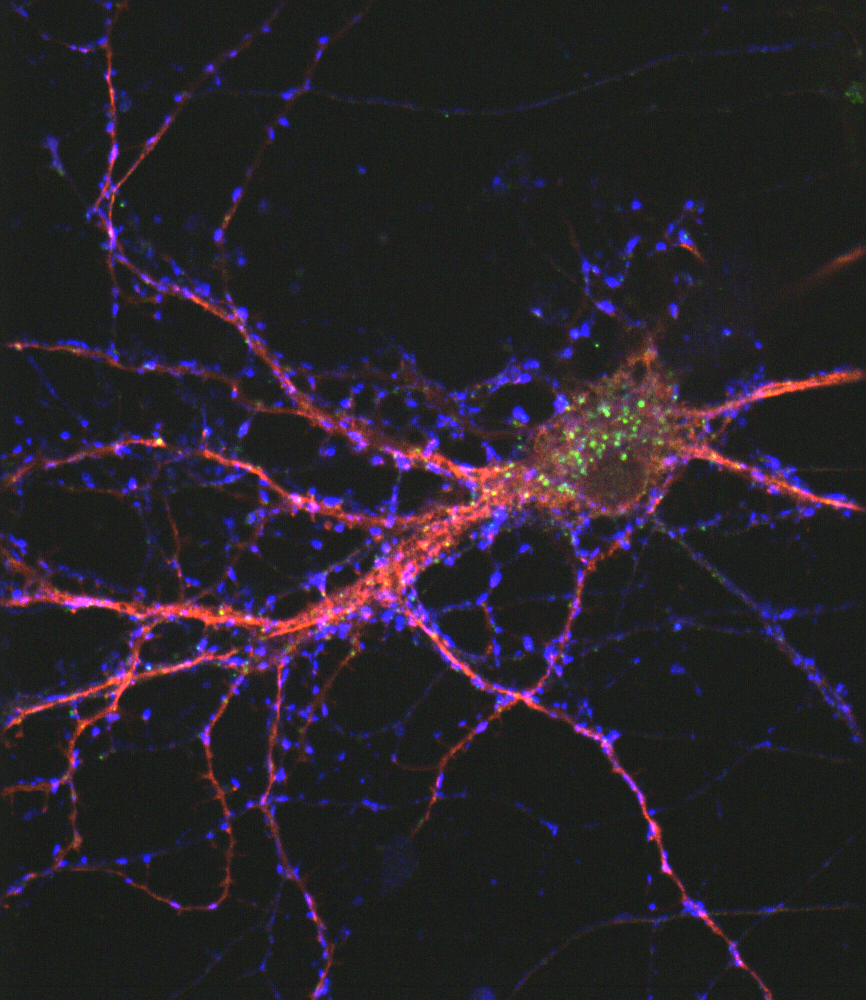

Supplement: Supplementary file 4 — Source Data for Figure 1 [file EMBJ-41-e109352-s009.zip › Figure_1_Source_Data+Statistics/Figure_1I-J/Figure_1I_Full_Image_AP5.tif]

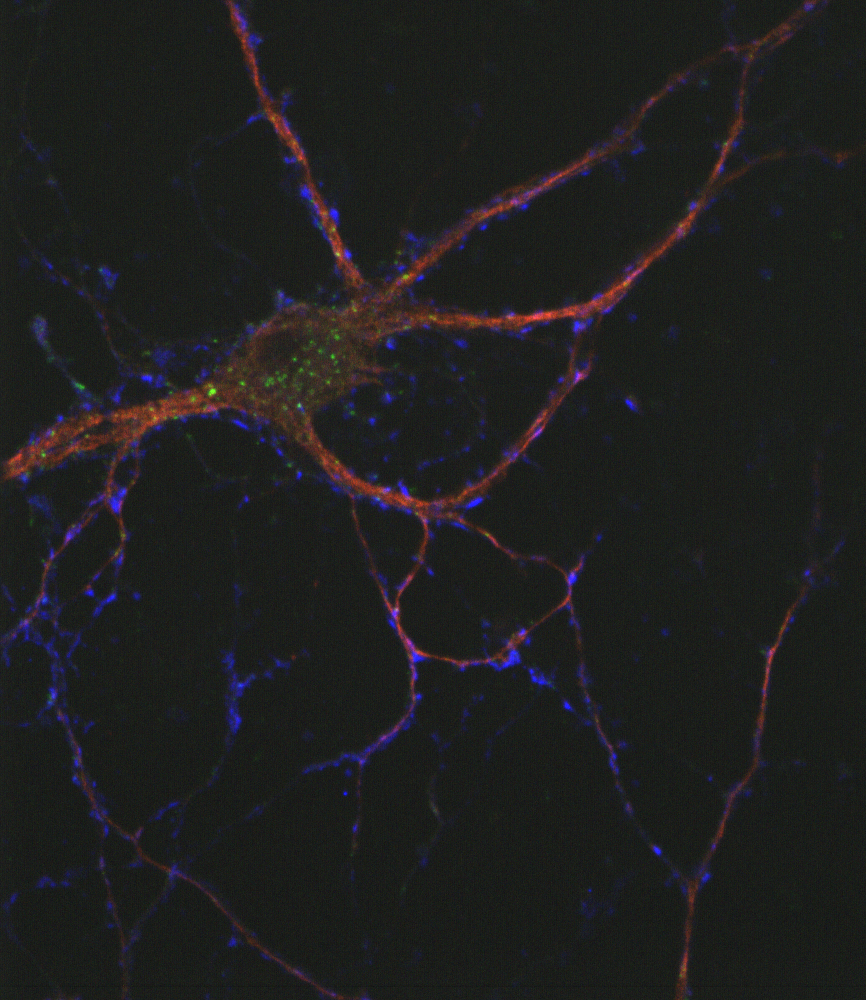

Supplement: Supplementary file 4 — Source Data for Figure 1 [file EMBJ-41-e109352-s009.zip › Figure_1_Source_Data+Statistics/Figure_1I-J/Figure_1I_Full_Image_CNQX.tif]

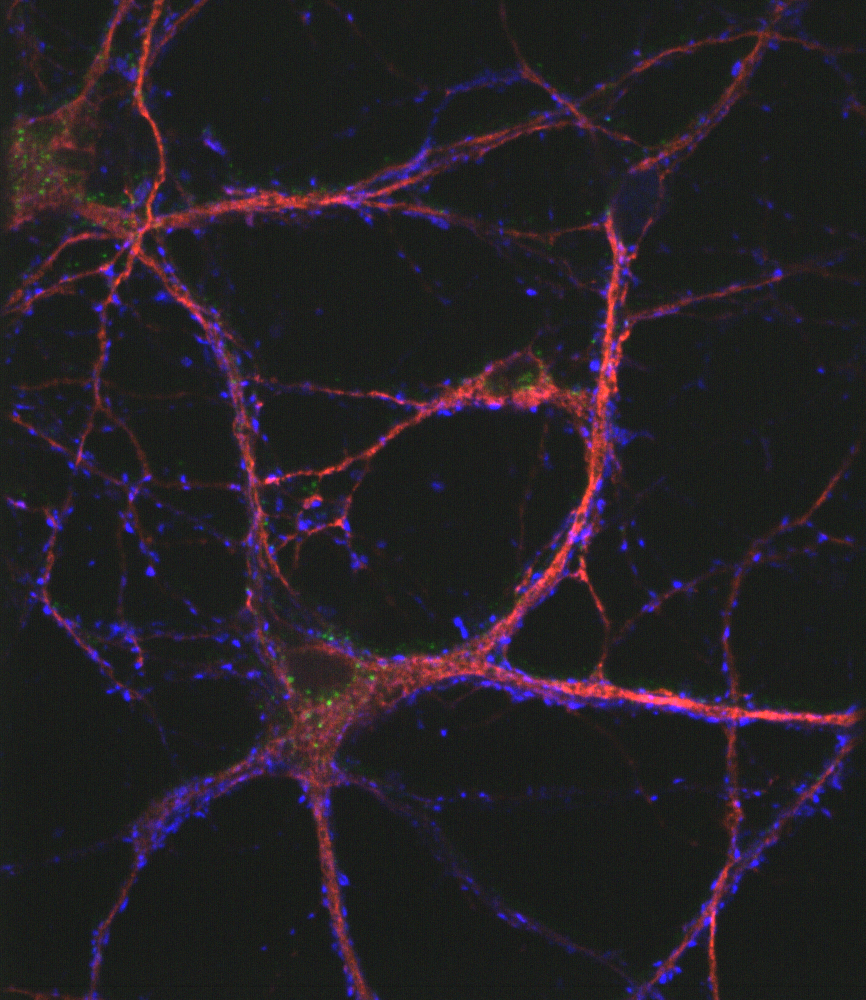

Supplement: Supplementary file 4 — Source Data for Figure 1 [file EMBJ-41-e109352-s009.zip › Figure_1_Source_Data+Statistics/Figure_1I-J/Figure_1I_Full_Image_DMSO.tif]

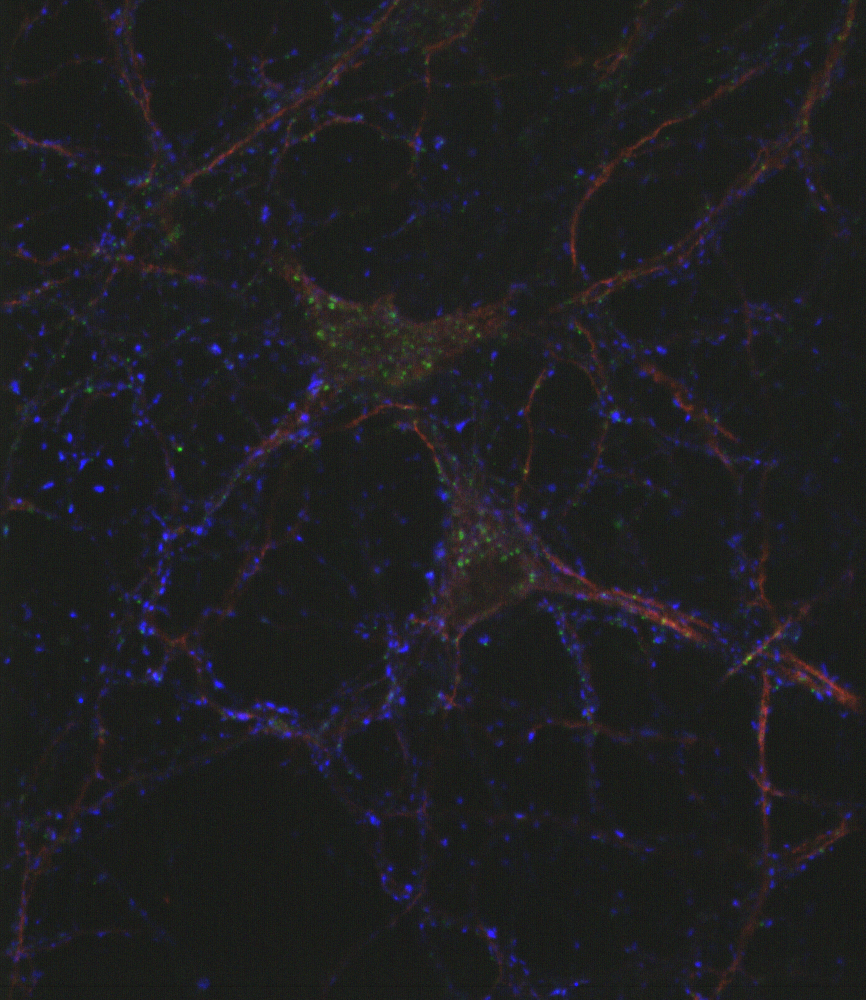

Supplement: Supplementary file 4 — Source Data for Figure 1 [file EMBJ-41-e109352-s009.zip › Figure_1_Source_Data+Statistics/Figure_1I-J/Figure_1I_Full_Image_TTX.tif]

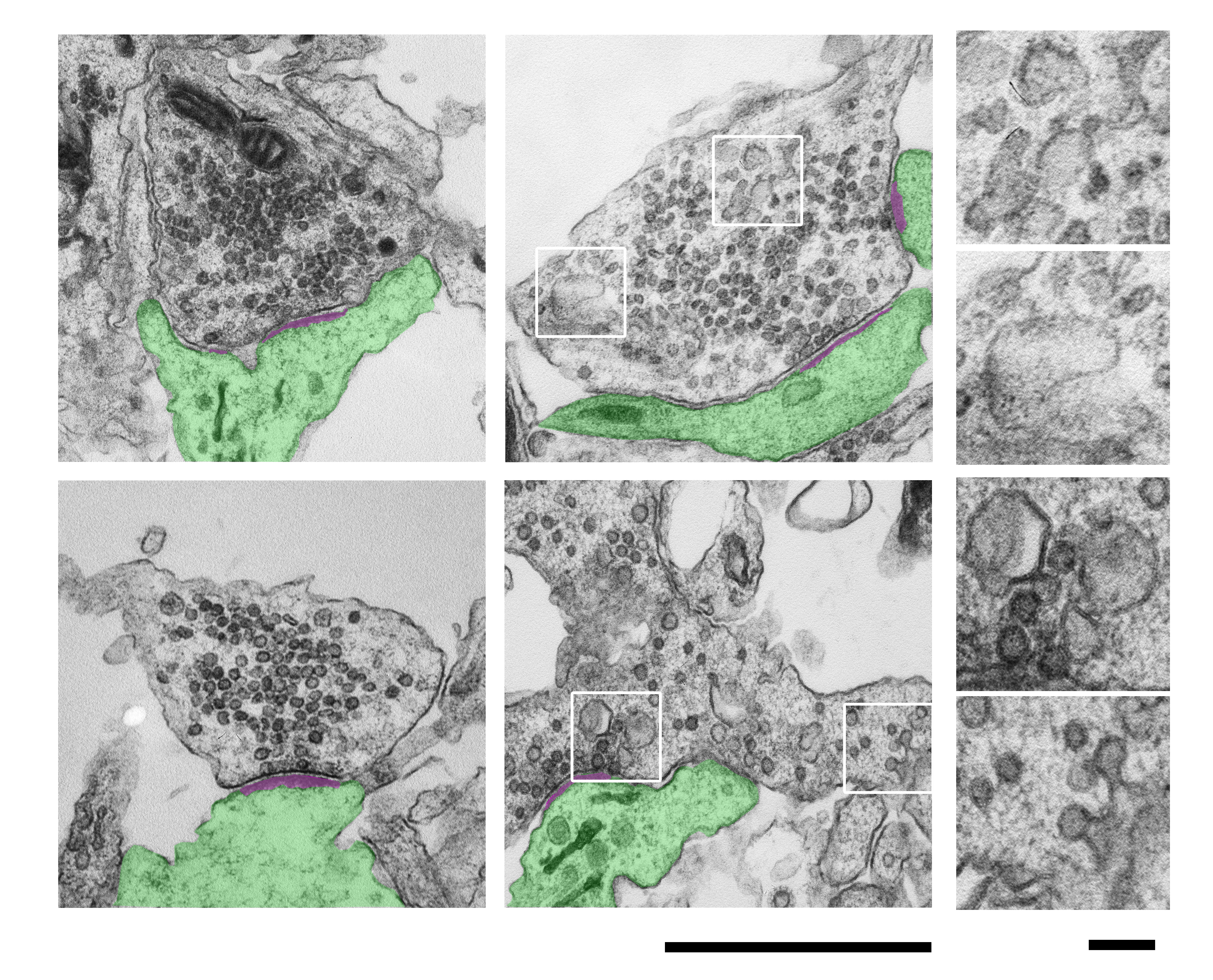

Supplement: Supplementary file 6 — Source Data for Figure 3 [file EMBJ-41-e109352-s005.zip › Figure_3_Source_Data+Statistics/Figure_3A.tif]

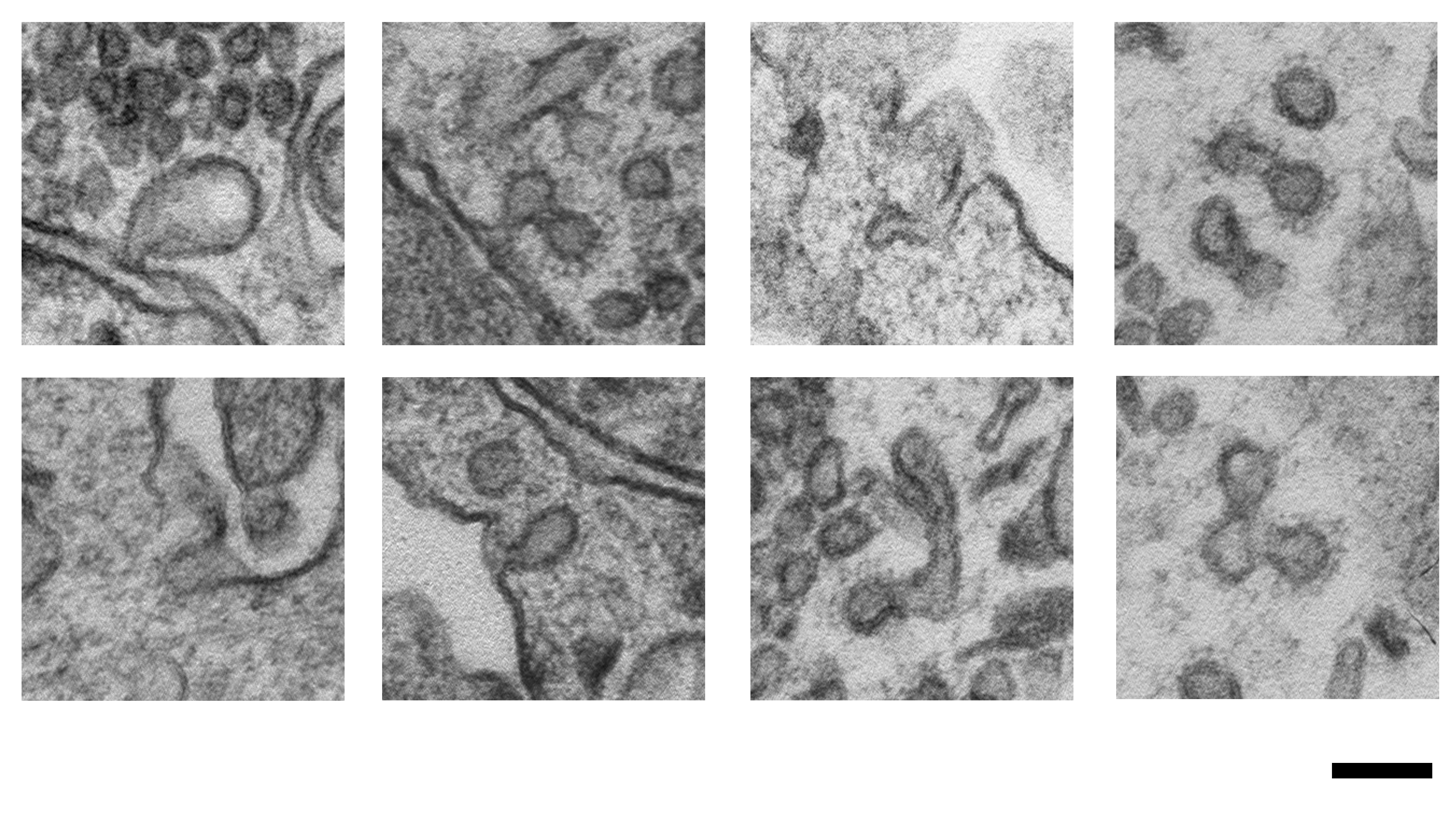

Supplement: Supplementary file 6 — Source Data for Figure 3 [file EMBJ-41-e109352-s005.zip › Figure_3_Source_Data+Statistics/Figure_3B.tif]

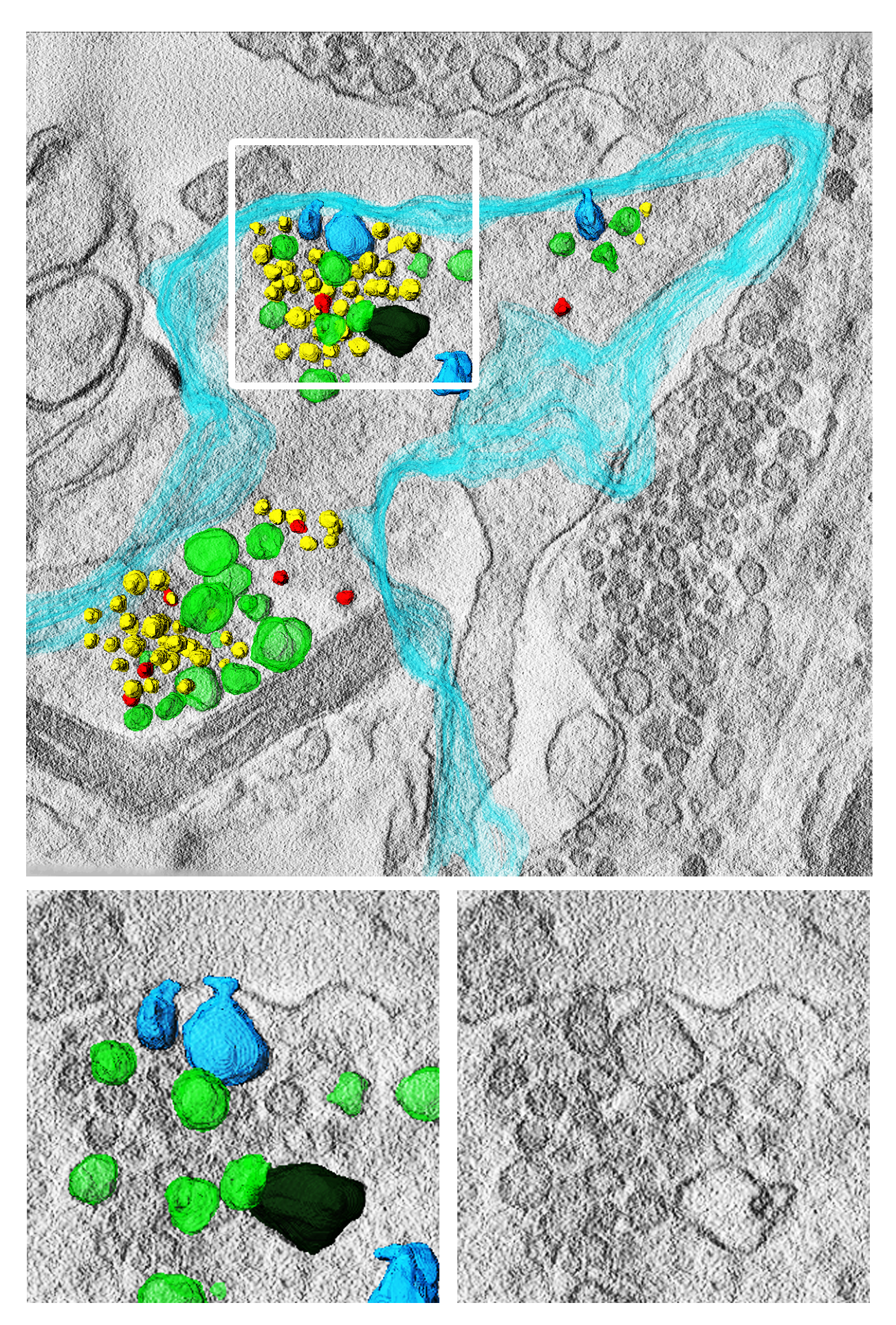

Supplement: Supplementary file 6 — Source Data for Figure 3 [file EMBJ-41-e109352-s005.zip › Figure_3_Source_Data+Statistics/Figure_3C.tif]

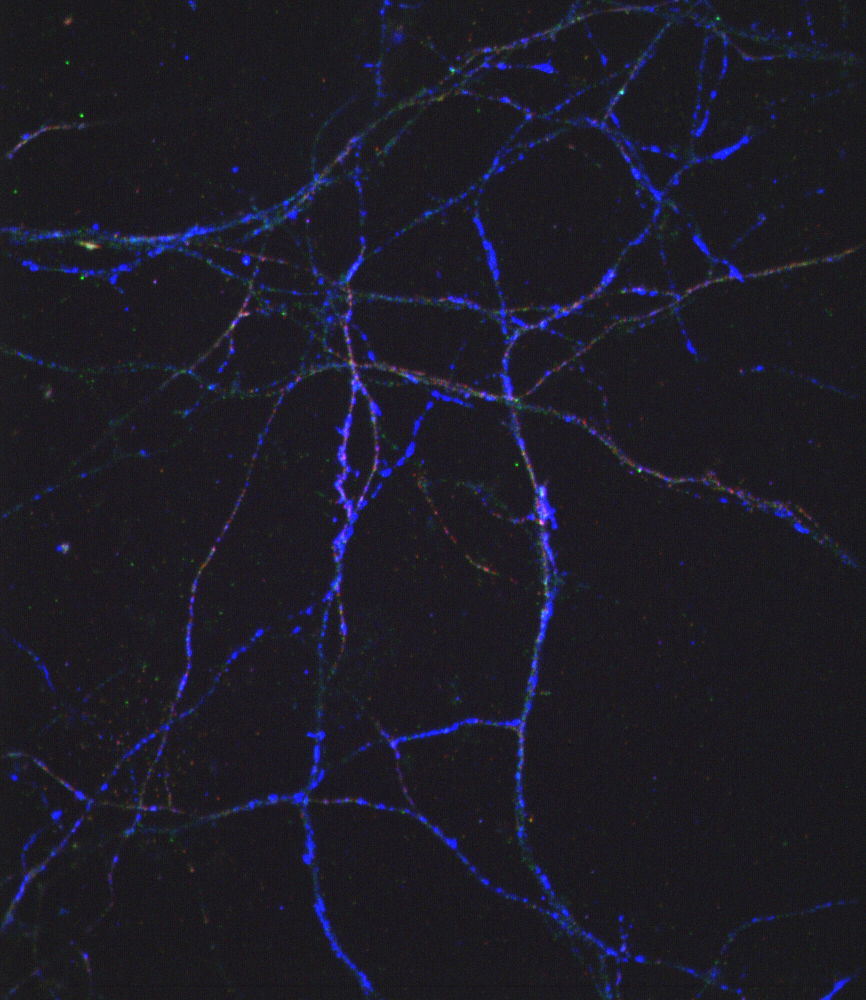

Supplement: Supplementary file 7 — Source Data for Figure 4 [file EMBJ-41-e109352-s003.zip › Figure_4_Source_Data+Statistics/Figure_4E-F/Figure_4F_Raw_Images/DMSO_nonStim/DMSO_syt_surf(R)total(G)vGlut(B)_1.tif]

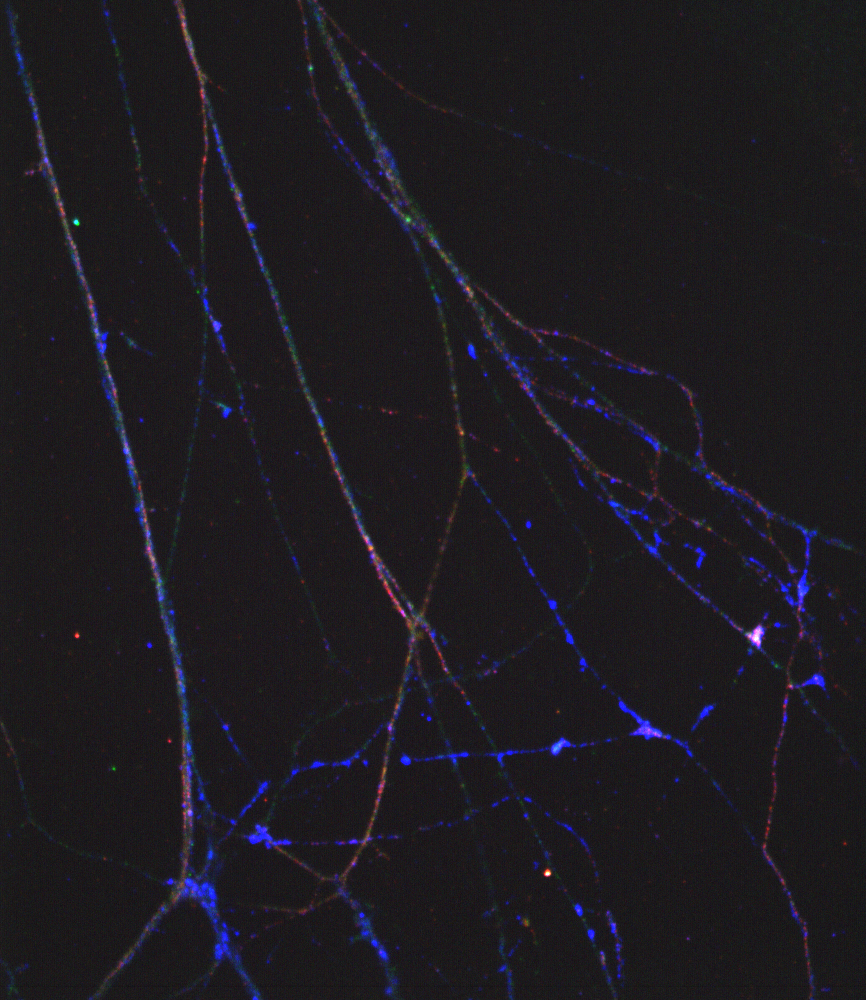

Supplement: Supplementary file 7 — Source Data for Figure 4 [file EMBJ-41-e109352-s003.zip › Figure_4_Source_Data+Statistics/Figure_4E-F/Figure_4F_Raw_Images/DMSO_nonStim/DMSO_syt_surf(R)total(G)vGlut(B)_10.tif]

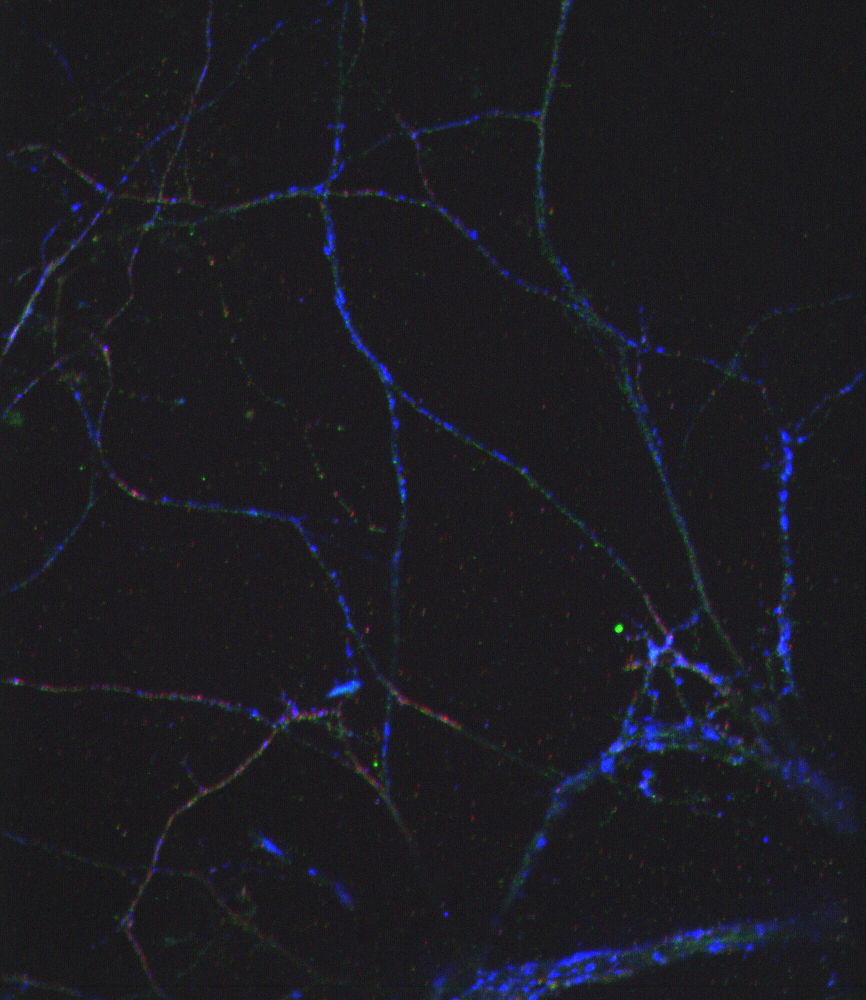

Supplement: Supplementary file 7 — Source Data for Figure 4 [file EMBJ-41-e109352-s003.zip › Figure_4_Source_Data+Statistics/Figure_4E-F/Figure_4F_Raw_Images/DMSO_nonStim/DMSO_syt_surf(R)total(G)vGlut(B)_2.tif]

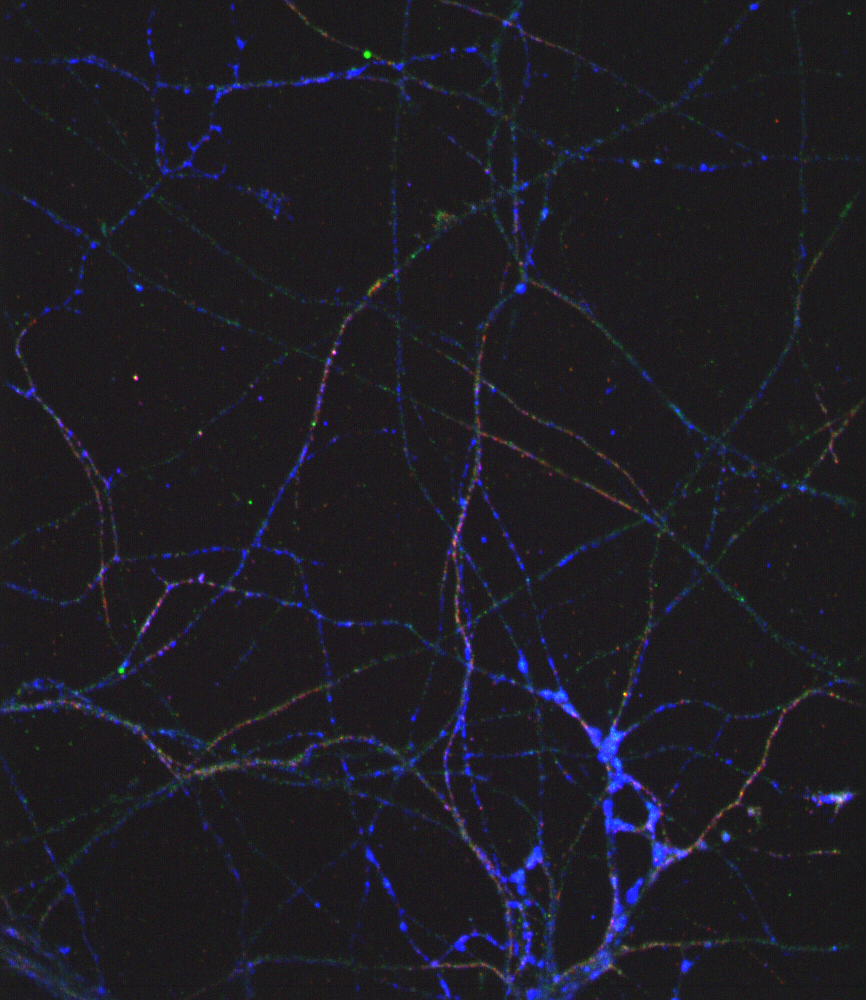

Supplement: Supplementary file 7 — Source Data for Figure 4 [file EMBJ-41-e109352-s003.zip › Figure_4_Source_Data+Statistics/Figure_4E-F/Figure_4F_Raw_Images/DMSO_nonStim/DMSO_syt_surf(R)total(G)vGlut(B)_3.tif]

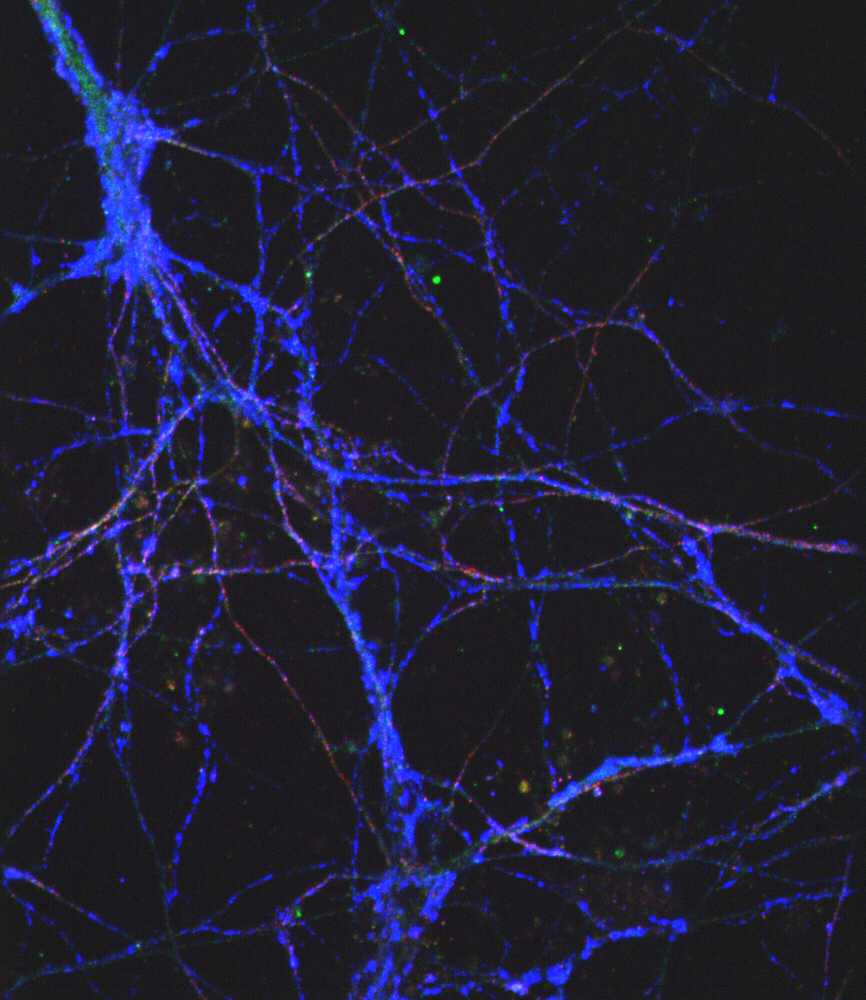

Supplement: Supplementary file 7 — Source Data for Figure 4 [file EMBJ-41-e109352-s003.zip › Figure_4_Source_Data+Statistics/Figure_4E-F/Figure_4F_Raw_Images/DMSO_nonStim/DMSO_syt_surf(R)total(G)vGlut(B)_4.tif]

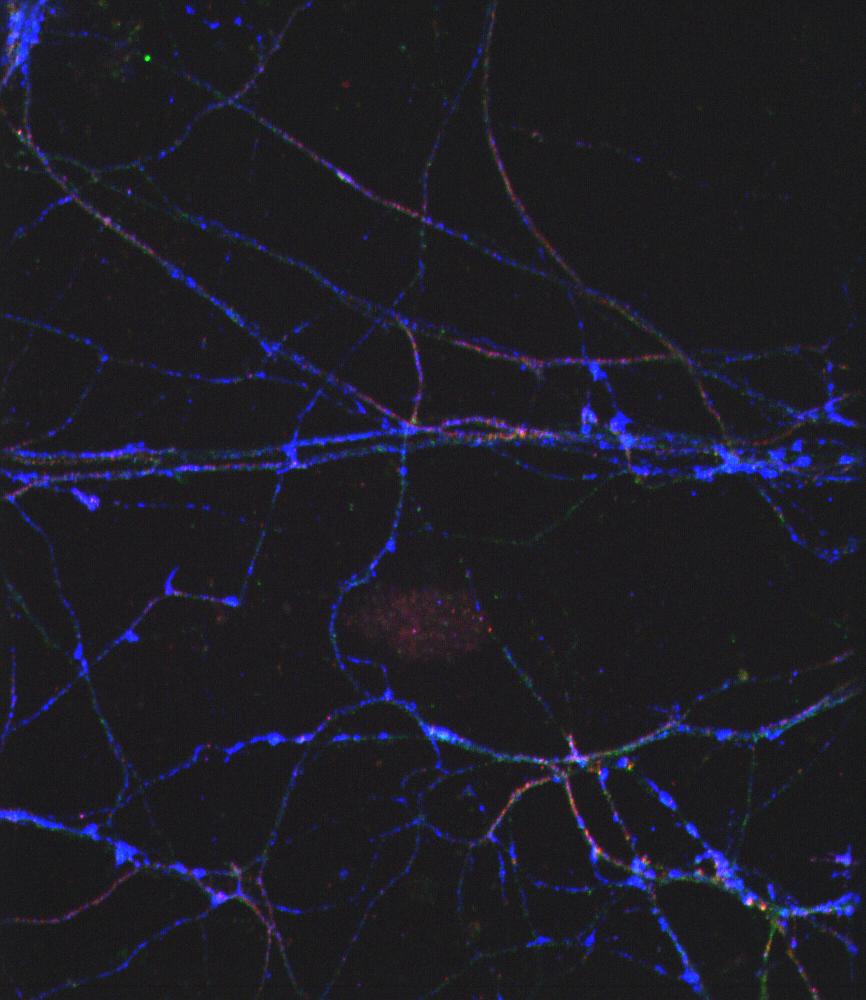

Supplement: Supplementary file 7 — Source Data for Figure 4 [file EMBJ-41-e109352-s003.zip › Figure_4_Source_Data+Statistics/Figure_4E-F/Figure_4F_Raw_Images/DMSO_nonStim/DMSO_syt_surf(R)total(G)vGlut(B)_5.tif]

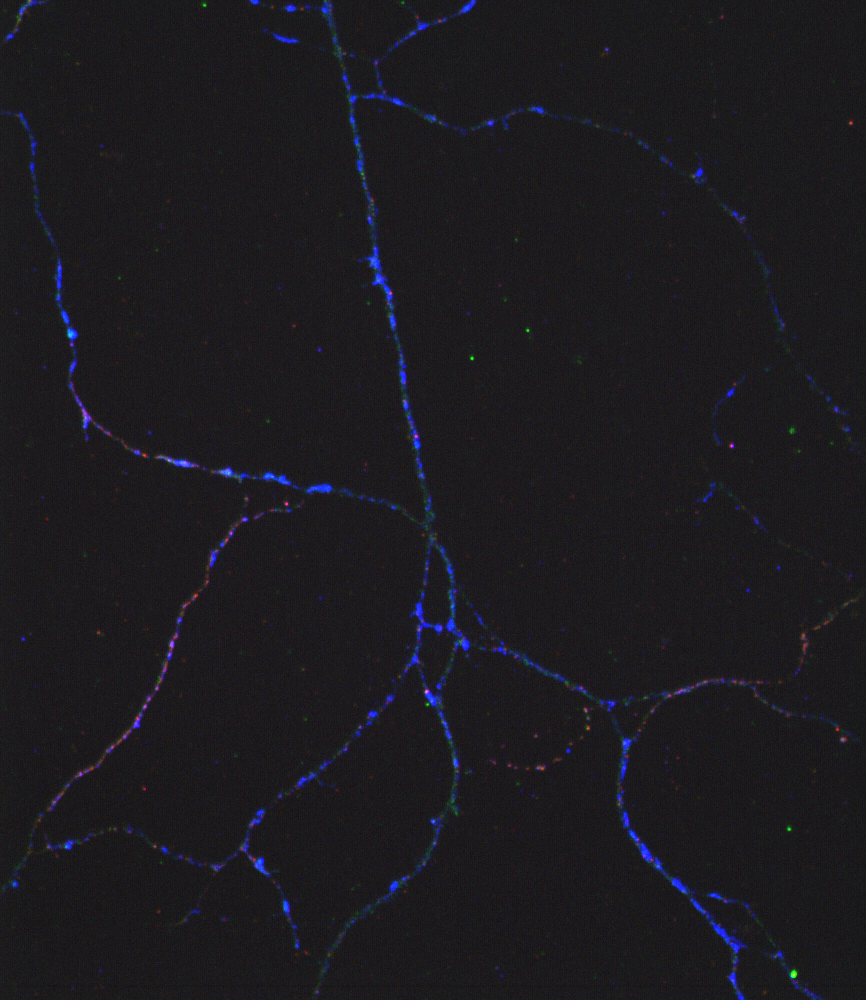

Supplement: Supplementary file 7 — Source Data for Figure 4 [file EMBJ-41-e109352-s003.zip › Figure_4_Source_Data+Statistics/Figure_4E-F/Figure_4F_Raw_Images/DMSO_nonStim/DMSO_syt_surf(R)total(G)vGlut(B)_6.tif]

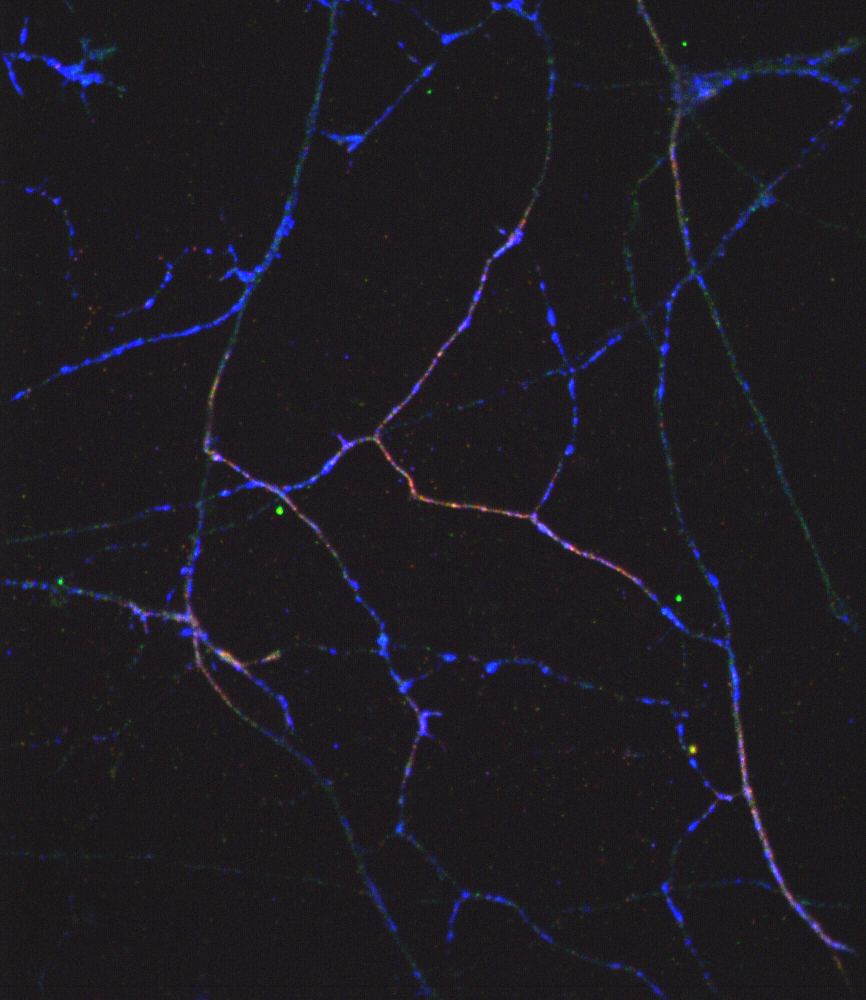

Supplement: Supplementary file 7 — Source Data for Figure 4 [file EMBJ-41-e109352-s003.zip › Figure_4_Source_Data+Statistics/Figure_4E-F/Figure_4F_Raw_Images/DMSO_nonStim/DMSO_syt_surf(R)total(G)vGlut(B)_7.tif]

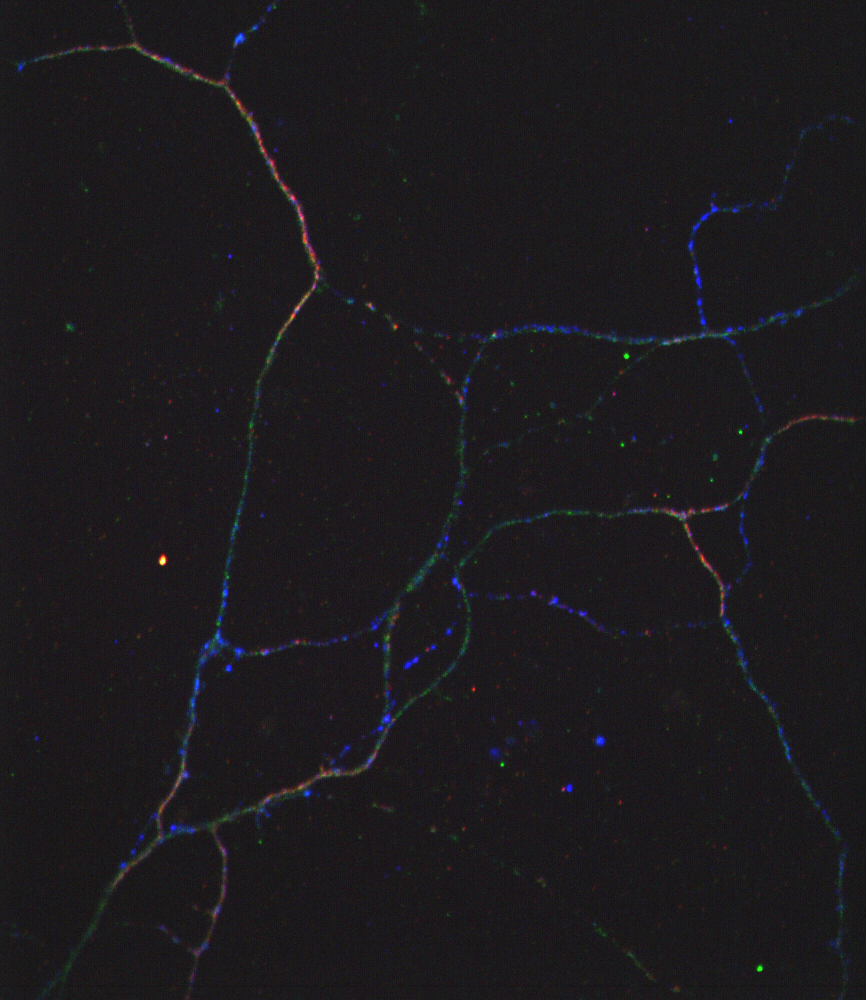

Supplement: Supplementary file 7 — Source Data for Figure 4 [file EMBJ-41-e109352-s003.zip › Figure_4_Source_Data+Statistics/Figure_4E-F/Figure_4F_Raw_Images/DMSO_nonStim/DMSO_syt_surf(R)total(G)vGlut(B)_8.tif]

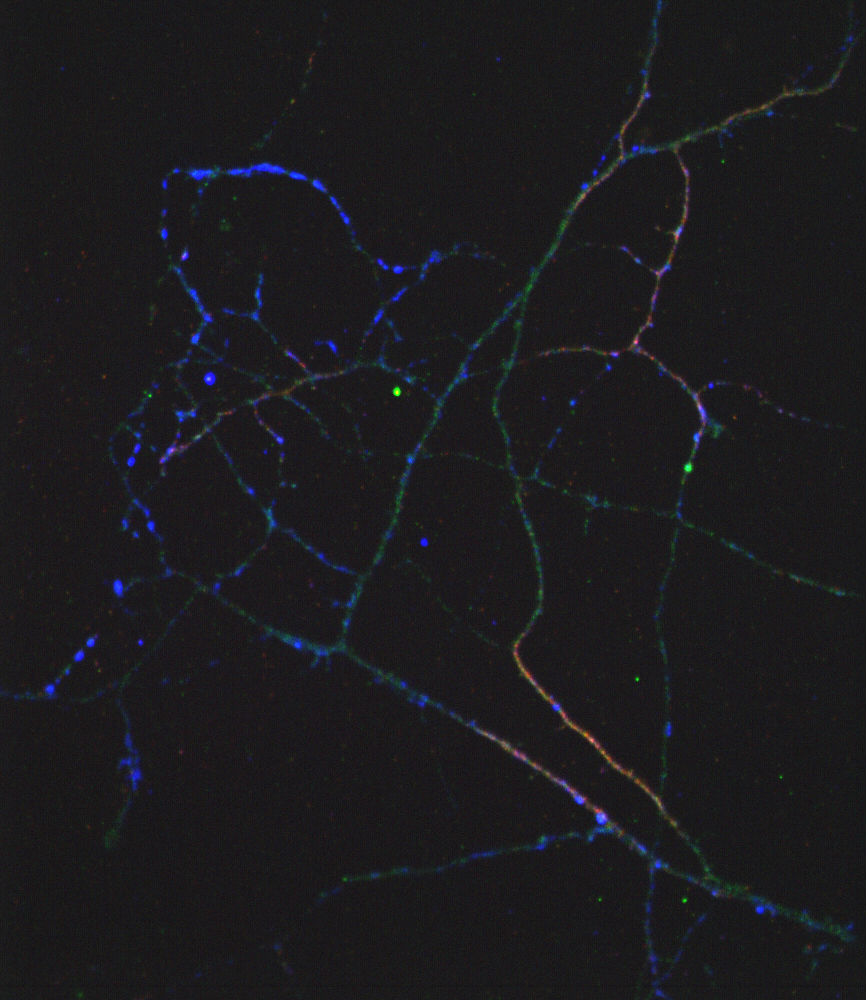

Supplement: Supplementary file 7 — Source Data for Figure 4 [file EMBJ-41-e109352-s003.zip › Figure_4_Source_Data+Statistics/Figure_4E-F/Figure_4F_Raw_Images/DMSO_nonStim/DMSO_syt_surf(R)total(G)vGlut(B)_9.tif]

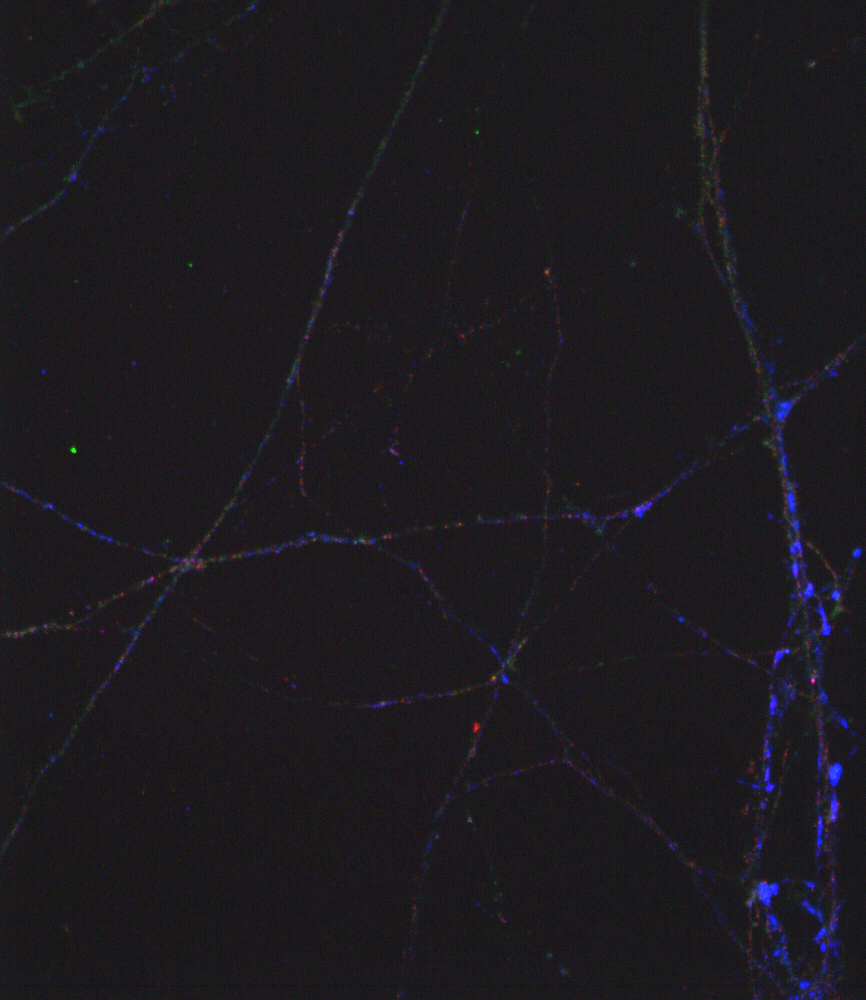

Supplement: Supplementary file 7 — Source Data for Figure 4 [file EMBJ-41-e109352-s003.zip › Figure_4_Source_Data+Statistics/Figure_4E-F/Figure_4F_Raw_Images/DMSO_Stim/DMSO_stim_syt_surf(R)total(G)vGlut(B).tif]

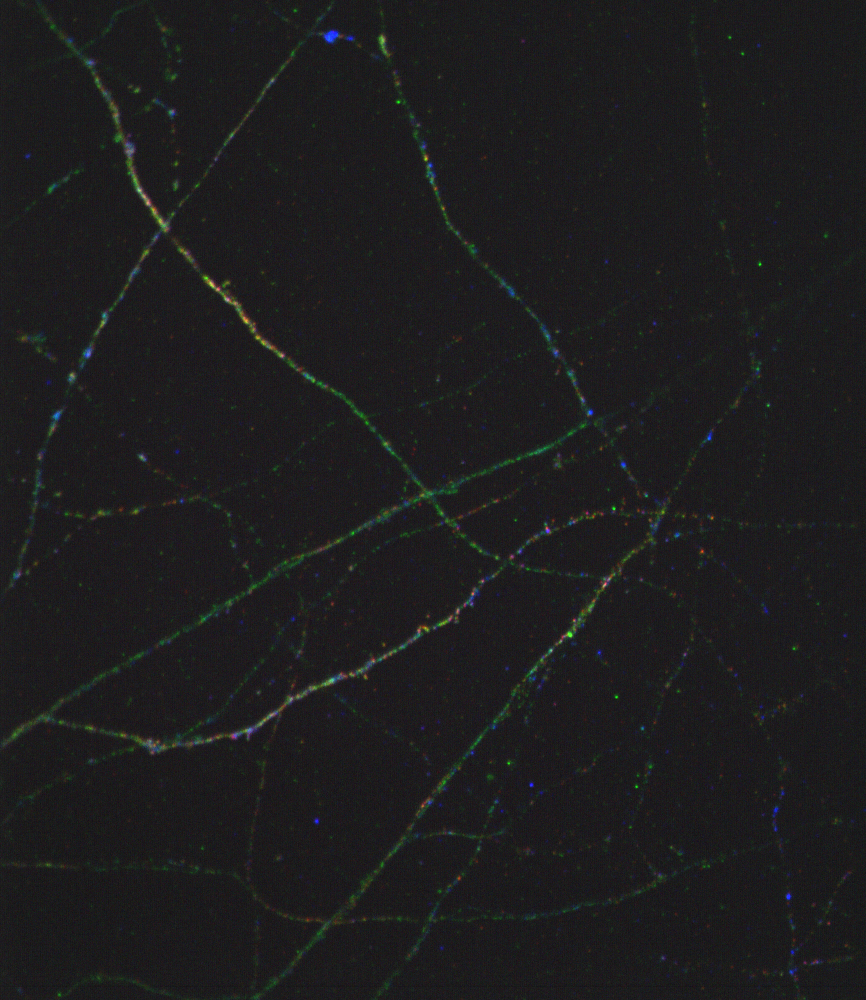

Supplement: Supplementary file 7 — Source Data for Figure 4 [file EMBJ-41-e109352-s003.zip › Figure_4_Source_Data+Statistics/Figure_4E-F/Figure_4F_Raw_Images/DMSO_Stim/DMSO_stim_syt_surf(R)total(G)vGlut(B)_10.tif]

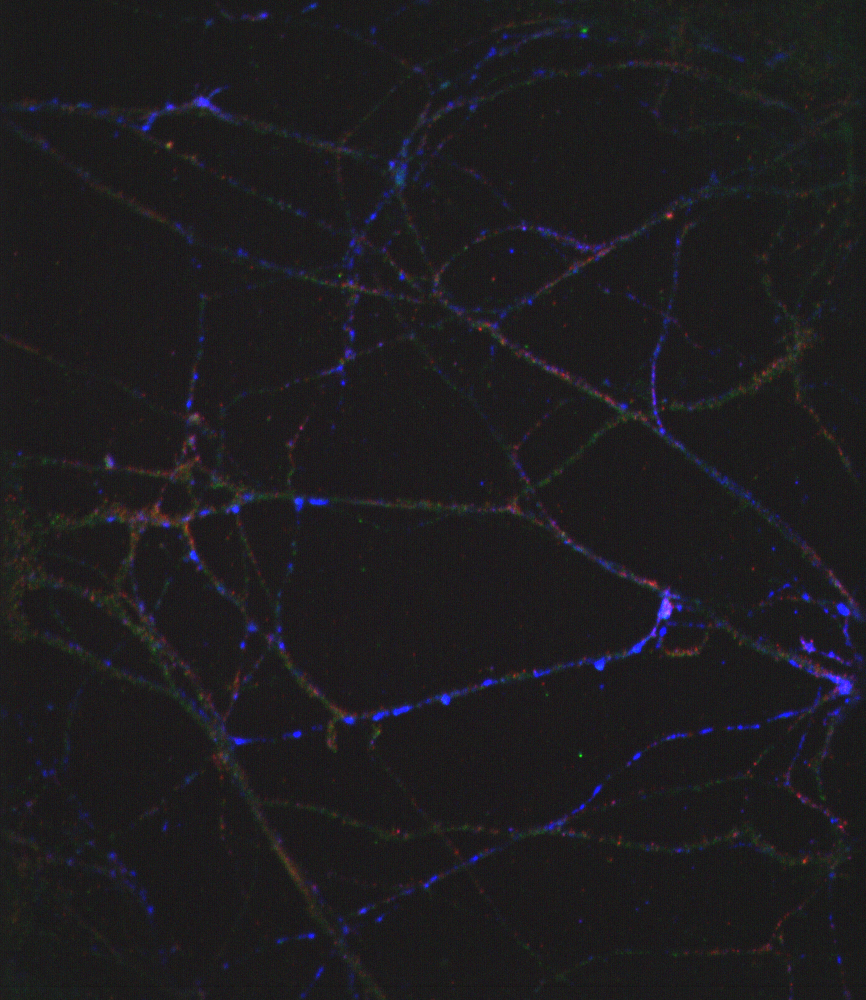

Supplement: Supplementary file 7 — Source Data for Figure 4 [file EMBJ-41-e109352-s003.zip › Figure_4_Source_Data+Statistics/Figure_4E-F/Figure_4F_Raw_Images/DMSO_Stim/DMSO_stim_syt_surf(R)total(G)vGlut(B)_2.tif]

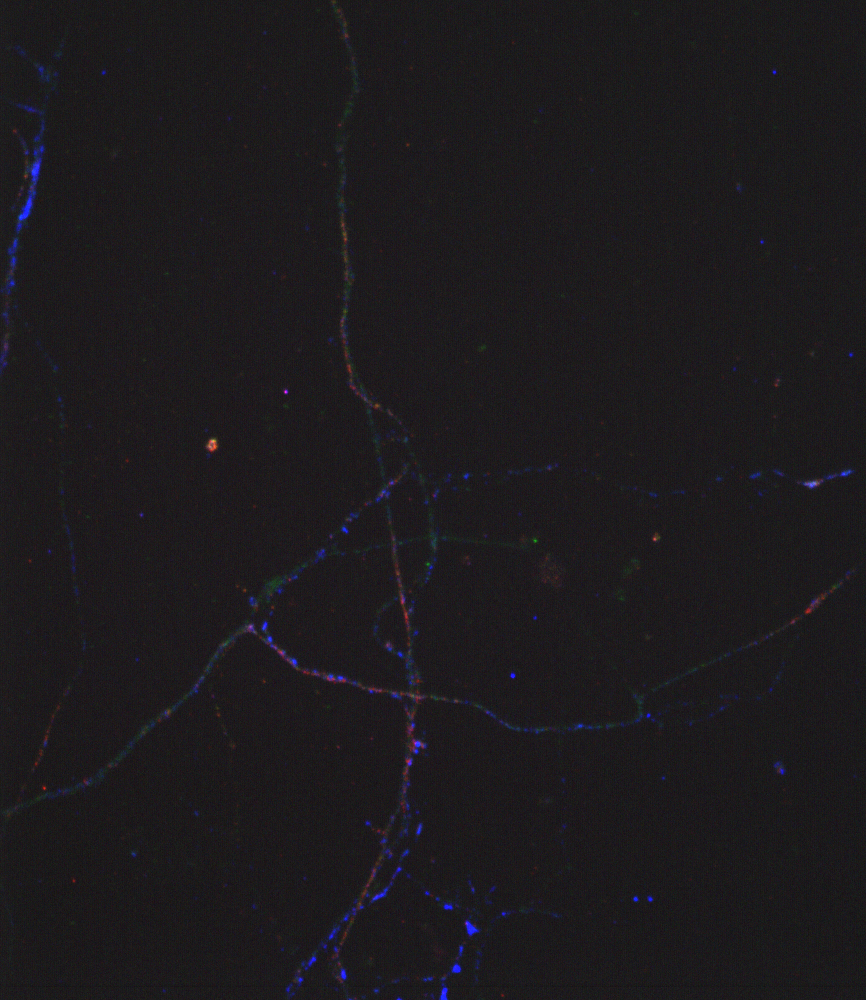

Supplement: Supplementary file 7 — Source Data for Figure 4 [file EMBJ-41-e109352-s003.zip › Figure_4_Source_Data+Statistics/Figure_4E-F/Figure_4F_Raw_Images/DMSO_Stim/DMSO_stim_syt_surf(R)total(G)vGlut(B)_3.tif]

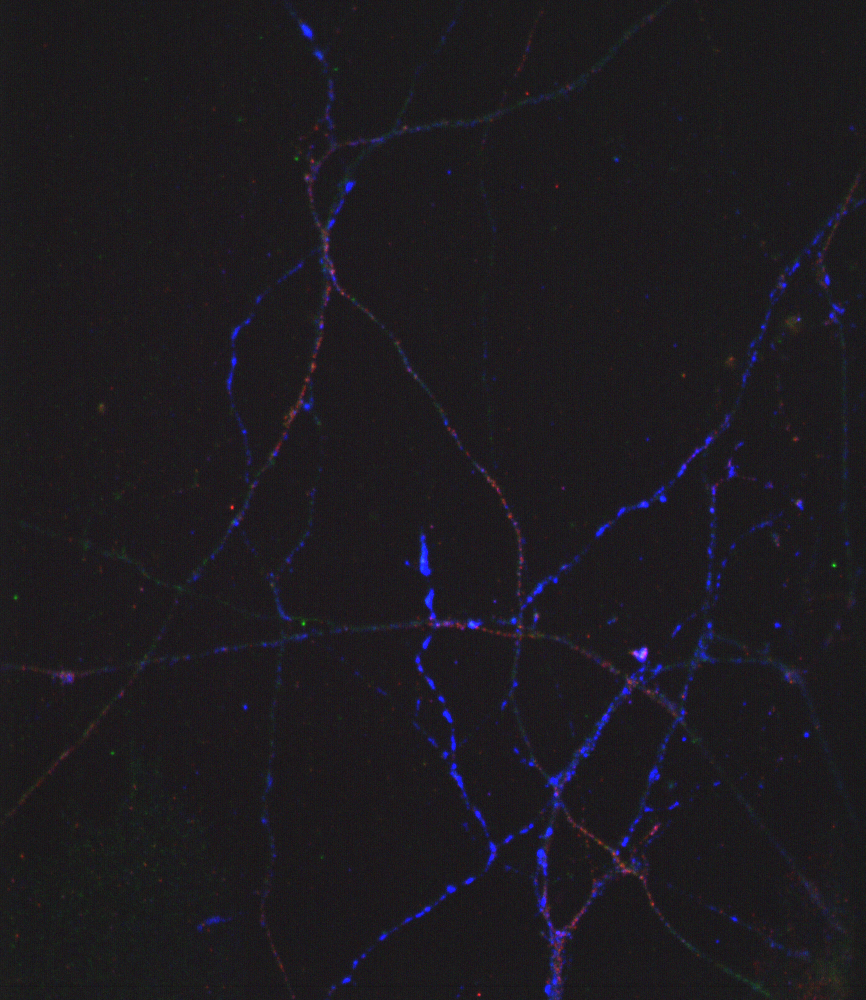

Supplement: Supplementary file 7 — Source Data for Figure 4 [file EMBJ-41-e109352-s003.zip › Figure_4_Source_Data+Statistics/Figure_4E-F/Figure_4F_Raw_Images/DMSO_Stim/DMSO_stim_syt_surf(R)total(G)vGlut(B)_4.tif]

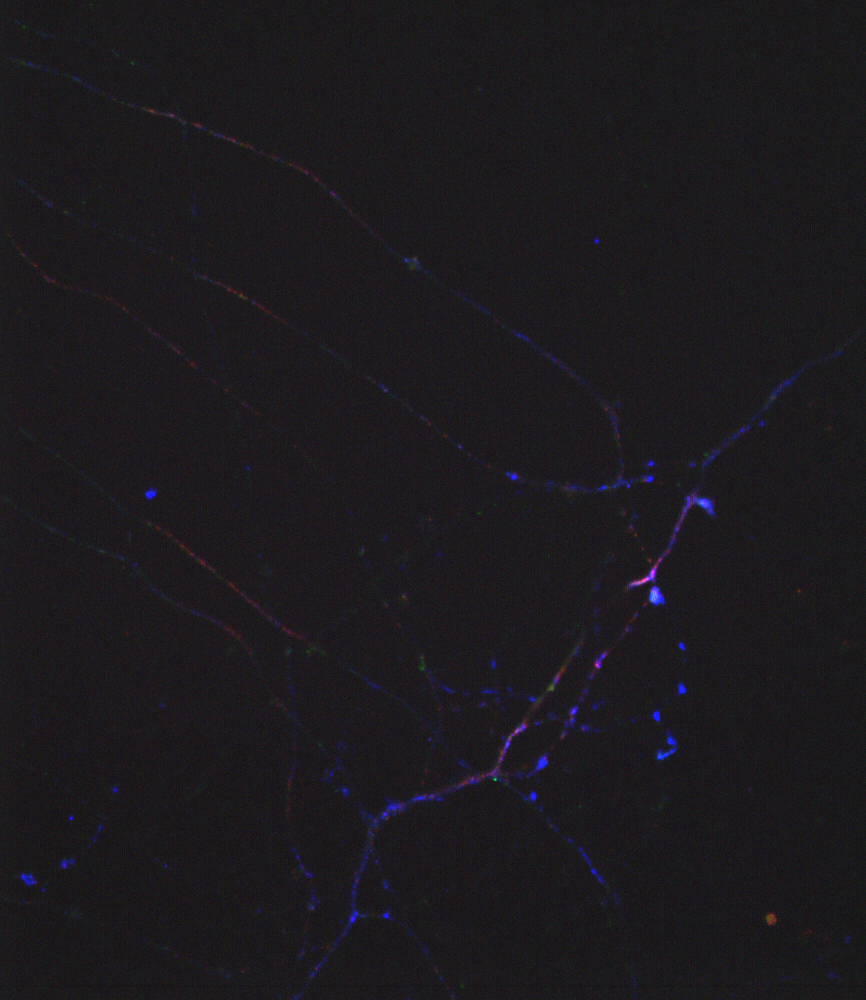

Supplement: Supplementary file 7 — Source Data for Figure 4 [file EMBJ-41-e109352-s003.zip › Figure_4_Source_Data+Statistics/Figure_4E-F/Figure_4F_Raw_Images/DMSO_Stim/DMSO_stim_syt_surf(R)total(G)vGlut(B)_5.tif]

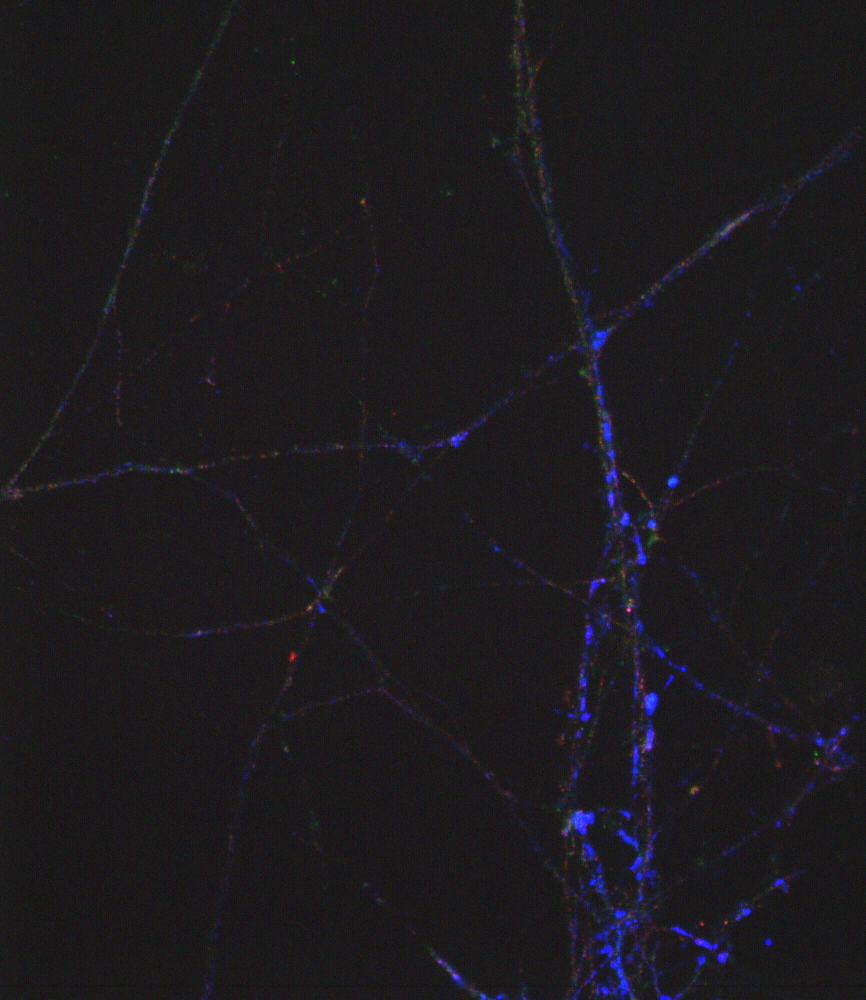

Supplement: Supplementary file 7 — Source Data for Figure 4 [file EMBJ-41-e109352-s003.zip › Figure_4_Source_Data+Statistics/Figure_4E-F/Figure_4F_Raw_Images/DMSO_Stim/DMSO_stim_syt_surf(R)total(G)vGlut(B)_6.tif]

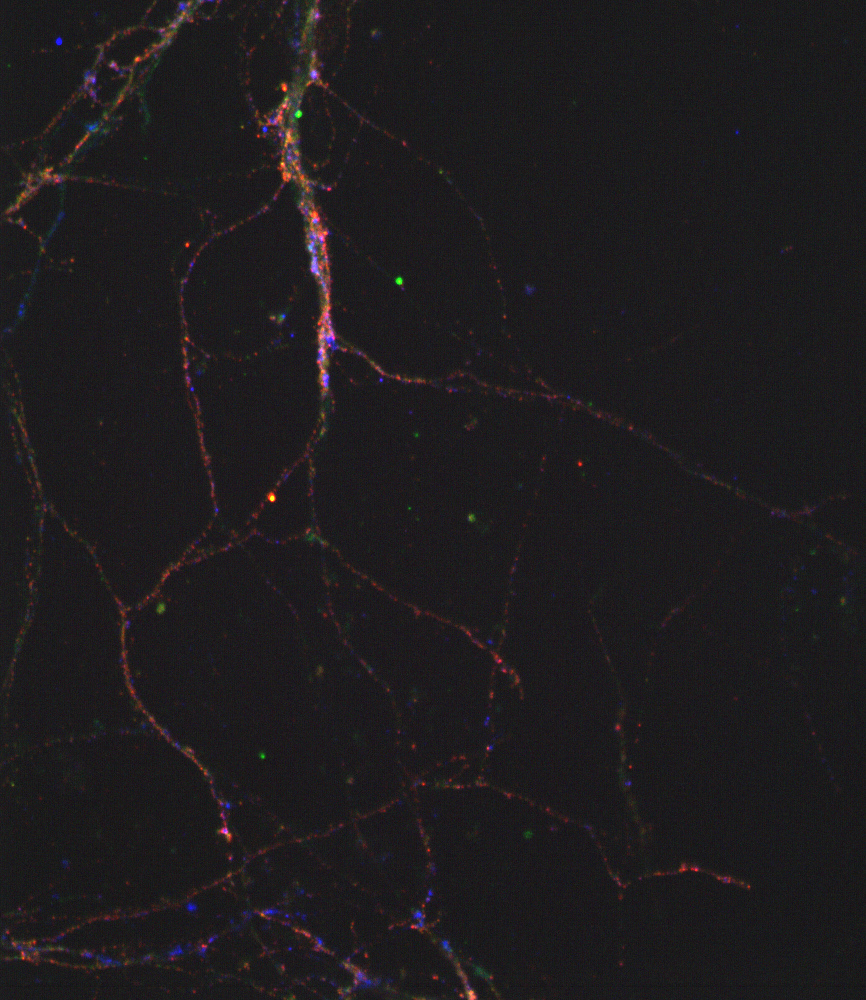

Supplement: Supplementary file 7 — Source Data for Figure 4 [file EMBJ-41-e109352-s003.zip › Figure_4_Source_Data+Statistics/Figure_4E-F/Figure_4F_Raw_Images/DMSO_Stim/DMSO_stim_syt_surf(R)total(G)vGlut(B)_7.tif]

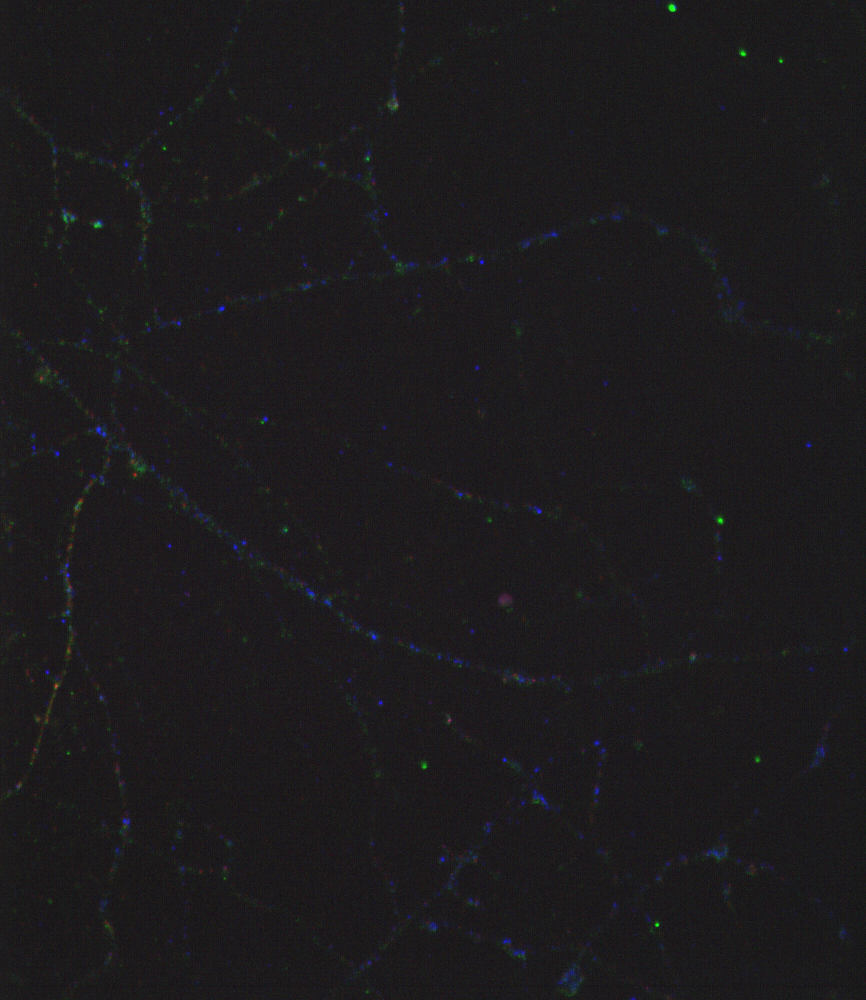

Supplement: Supplementary file 7 — Source Data for Figure 4 [file EMBJ-41-e109352-s003.zip › Figure_4_Source_Data+Statistics/Figure_4E-F/Figure_4F_Raw_Images/DMSO_Stim/DMSO_stim_syt_surf(R)total(G)vGlut(B)_8.tif]

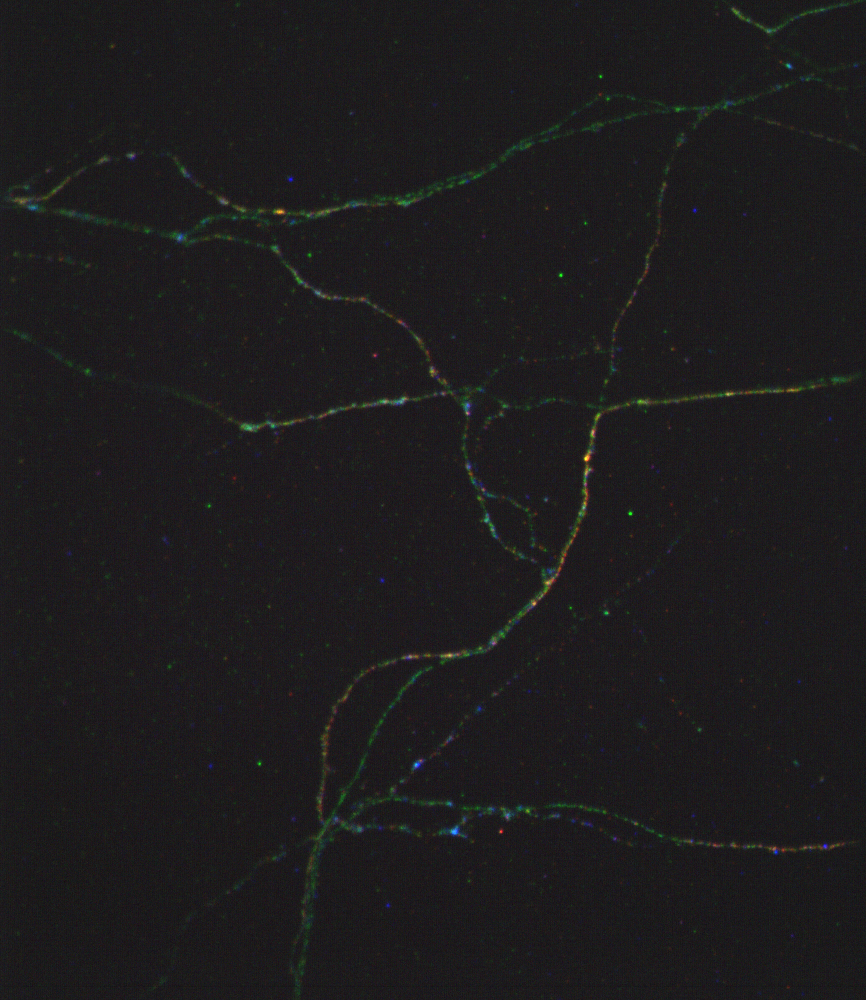

Supplement: Supplementary file 7 — Source Data for Figure 4 [file EMBJ-41-e109352-s003.zip › Figure_4_Source_Data+Statistics/Figure_4E-F/Figure_4F_Raw_Images/DMSO_Stim/DMSO_stim_syt_surf(R)total(G)vGlut(B)_9.tif]

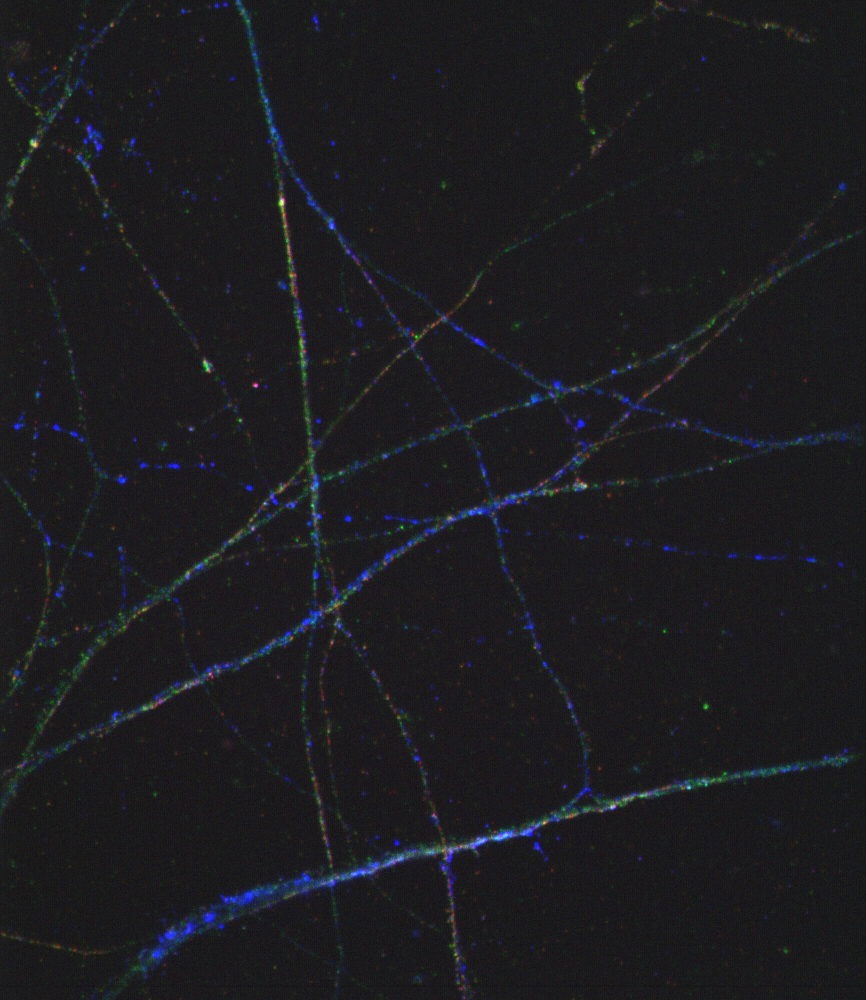

Supplement: Supplementary file 7 — Source Data for Figure 4 [file EMBJ-41-e109352-s003.zip › Figure_4_Source_Data+Statistics/Figure_4E-F/Figure_4F_Raw_Images/VPS34IN1_nonStim/34in_syt_surf(R)total(G)vGlut(B).tif]

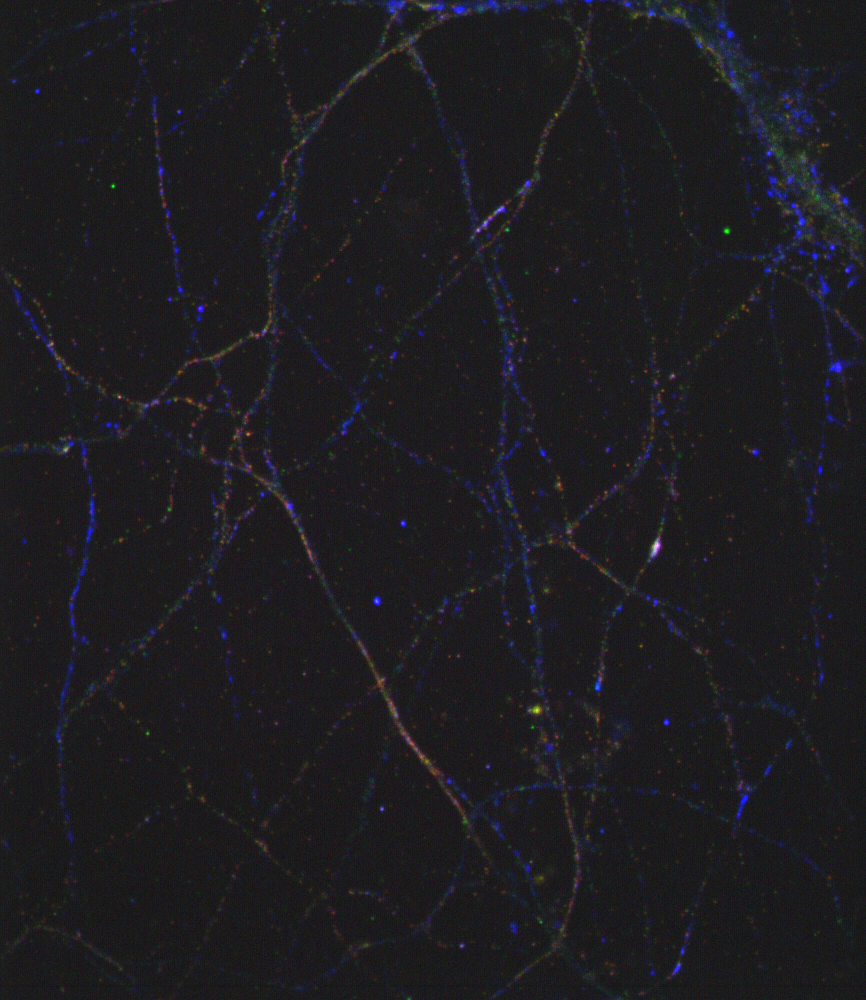

Supplement: Supplementary file 7 — Source Data for Figure 4 [file EMBJ-41-e109352-s003.zip › Figure_4_Source_Data+Statistics/Figure_4E-F/Figure_4F_Raw_Images/VPS34IN1_nonStim/34in_syt_surf(R)total(G)vGlut(B)_10.tif]

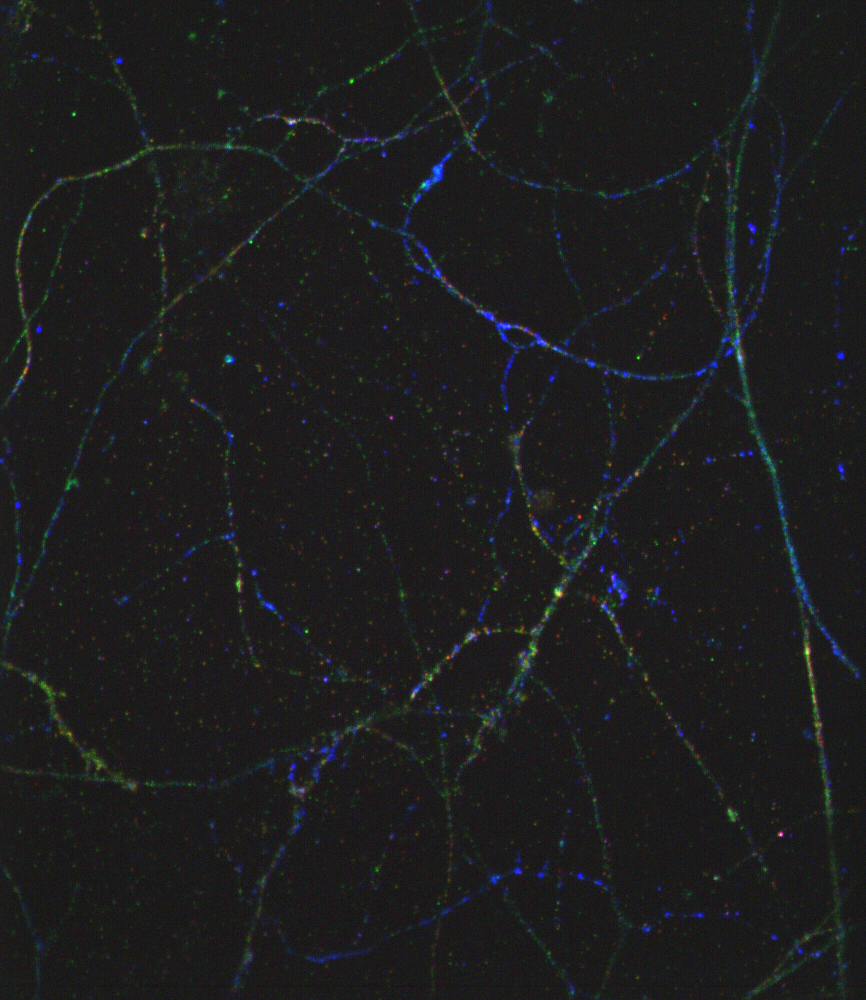

Supplement: Supplementary file 7 — Source Data for Figure 4 [file EMBJ-41-e109352-s003.zip › Figure_4_Source_Data+Statistics/Figure_4E-F/Figure_4F_Raw_Images/VPS34IN1_nonStim/34in_syt_surf(R)total(G)vGlut(B)_2.tif]

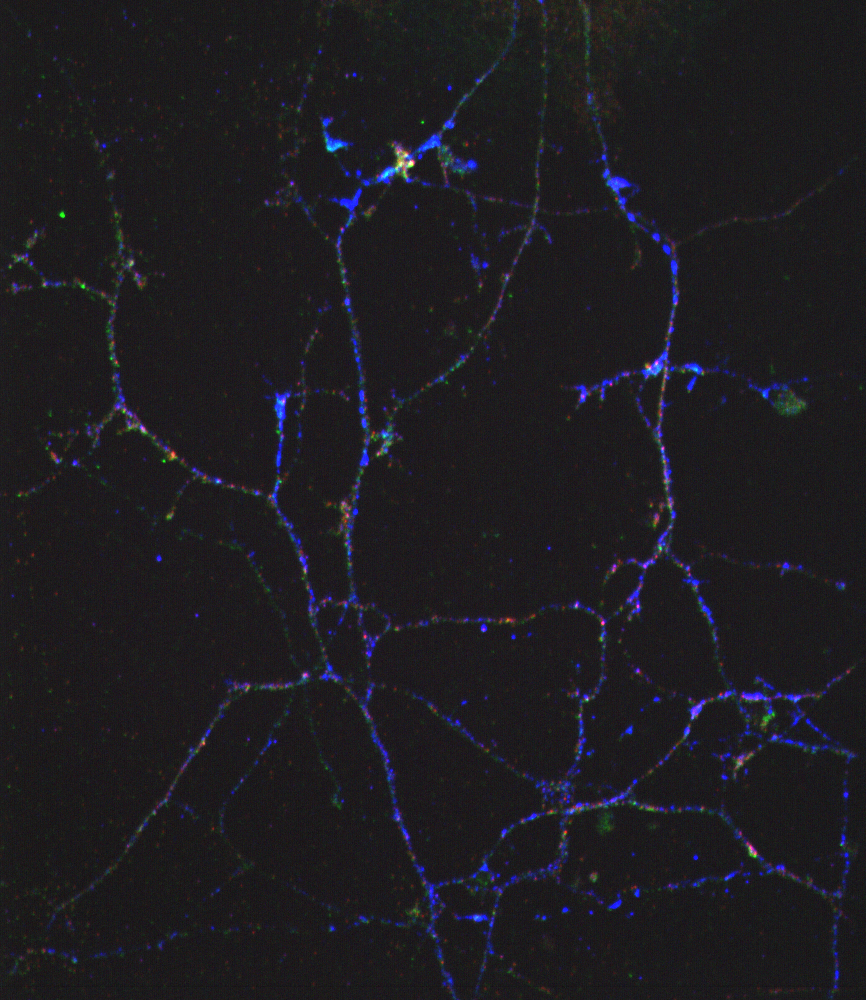

Supplement: Supplementary file 7 — Source Data for Figure 4 [file EMBJ-41-e109352-s003.zip › Figure_4_Source_Data+Statistics/Figure_4E-F/Figure_4F_Raw_Images/VPS34IN1_nonStim/34in_syt_surf(R)total(G)vGlut(B)_3.tif]

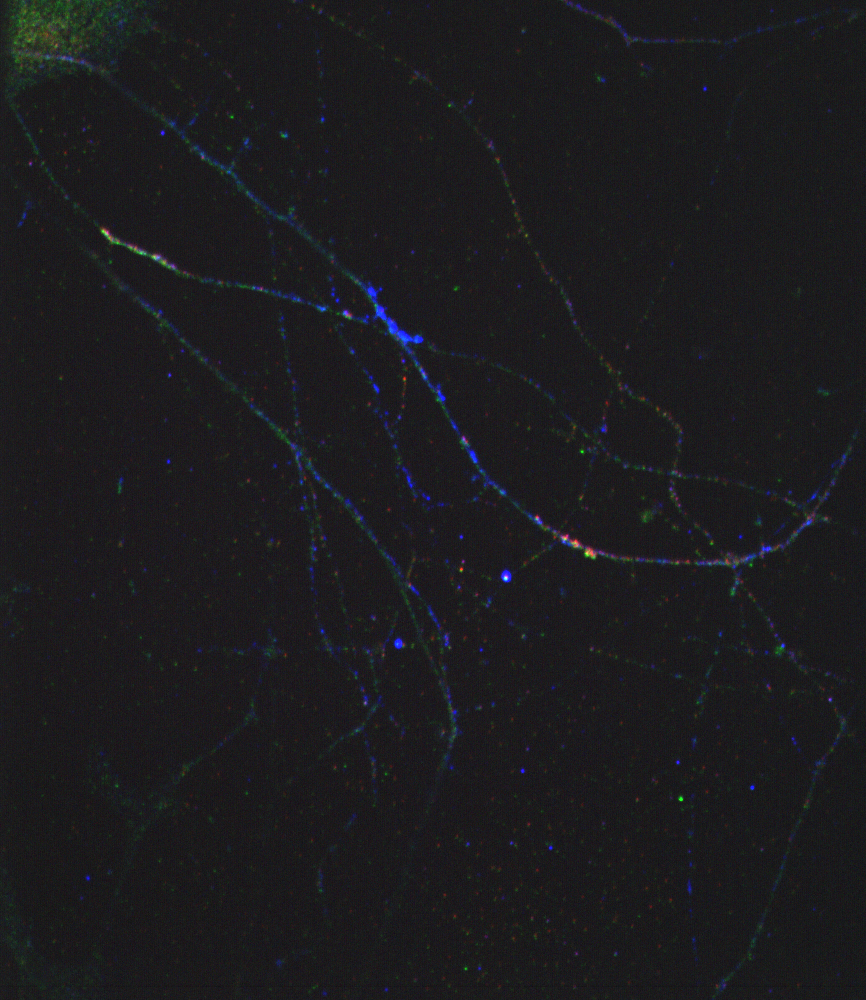

Supplement: Supplementary file 7 — Source Data for Figure 4 [file EMBJ-41-e109352-s003.zip › Figure_4_Source_Data+Statistics/Figure_4E-F/Figure_4F_Raw_Images/VPS34IN1_nonStim/34in_syt_surf(R)total(G)vGlut(B)_4.tif]

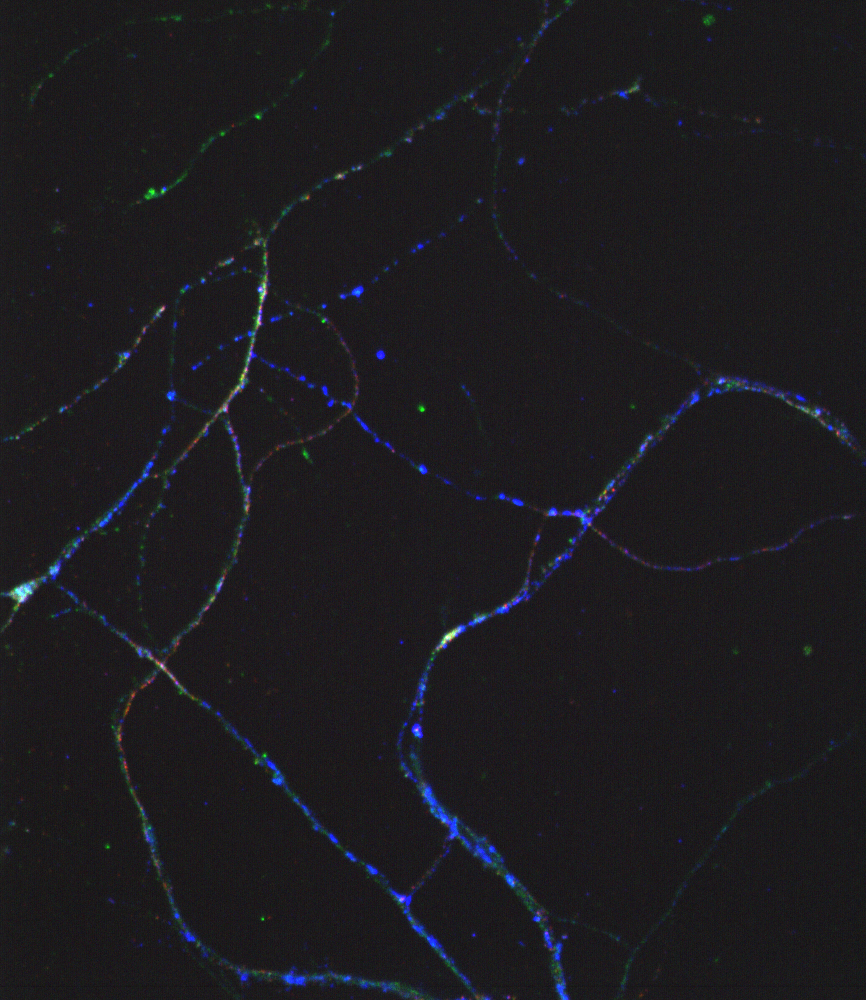

Supplement: Supplementary file 7 — Source Data for Figure 4 [file EMBJ-41-e109352-s003.zip › Figure_4_Source_Data+Statistics/Figure_4E-F/Figure_4F_Raw_Images/VPS34IN1_nonStim/34in_syt_surf(R)total(G)vGlut(B)_5.tif]

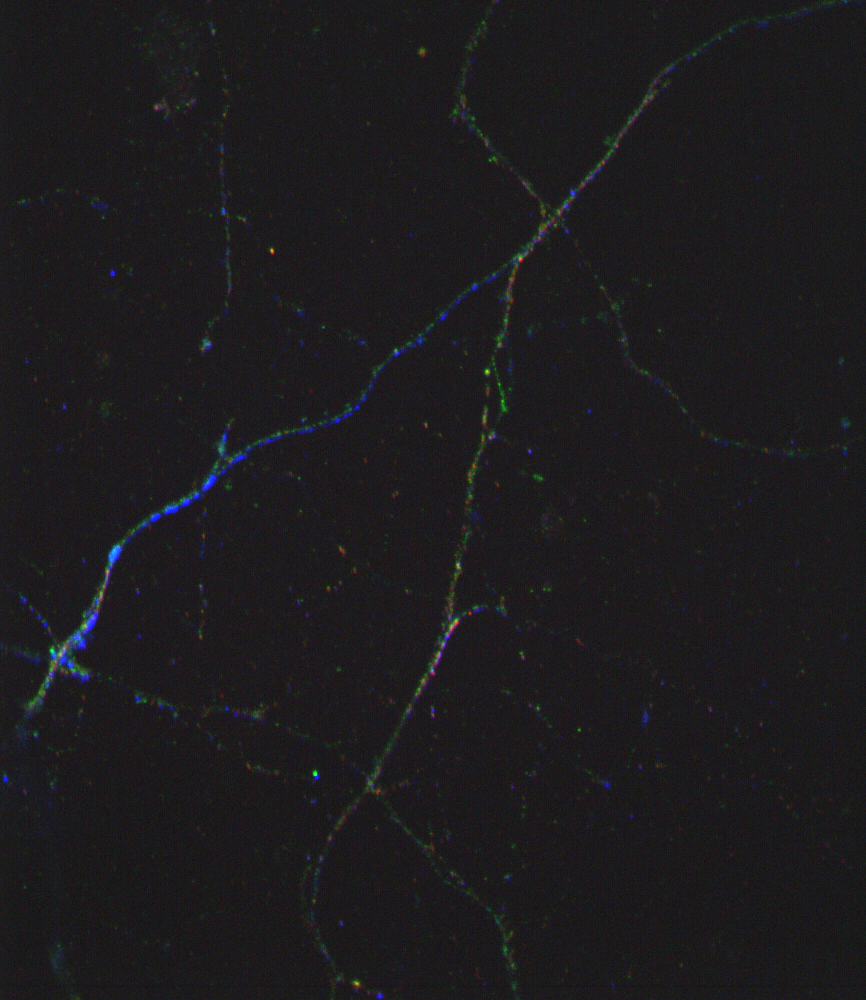

Supplement: Supplementary file 7 — Source Data for Figure 4 [file EMBJ-41-e109352-s003.zip › Figure_4_Source_Data+Statistics/Figure_4E-F/Figure_4F_Raw_Images/VPS34IN1_nonStim/34in_syt_surf(R)total(G)vGlut(B)_6.tif]

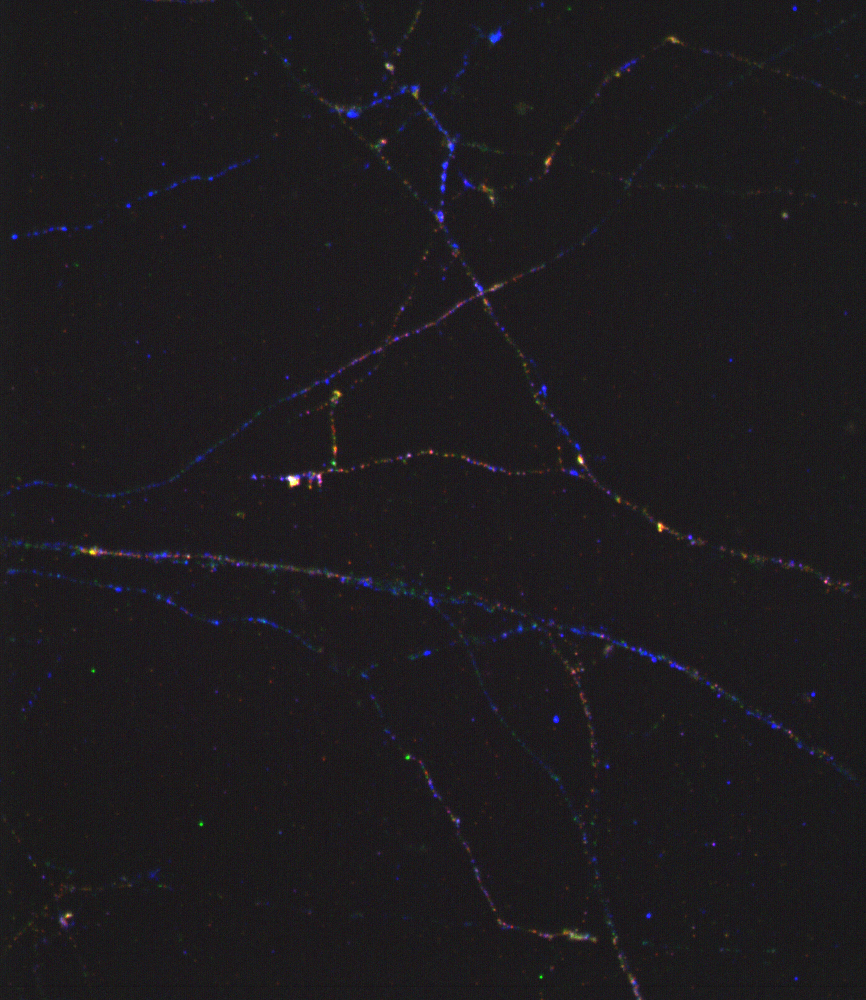

Supplement: Supplementary file 7 — Source Data for Figure 4 [file EMBJ-41-e109352-s003.zip › Figure_4_Source_Data+Statistics/Figure_4E-F/Figure_4F_Raw_Images/VPS34IN1_nonStim/34in_syt_surf(R)total(G)vGlut(B)_7.tif]

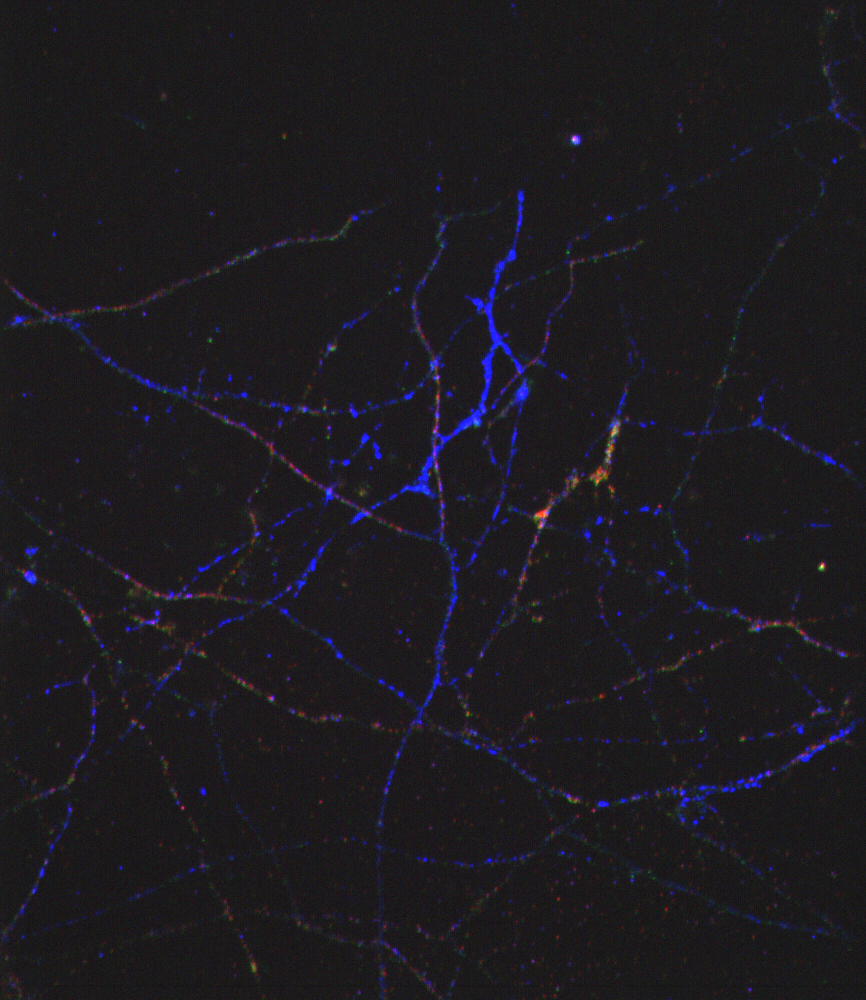

Supplement: Supplementary file 7 — Source Data for Figure 4 [file EMBJ-41-e109352-s003.zip › Figure_4_Source_Data+Statistics/Figure_4E-F/Figure_4F_Raw_Images/VPS34IN1_nonStim/34in_syt_surf(R)total(G)vGlut(B)_8.tif]

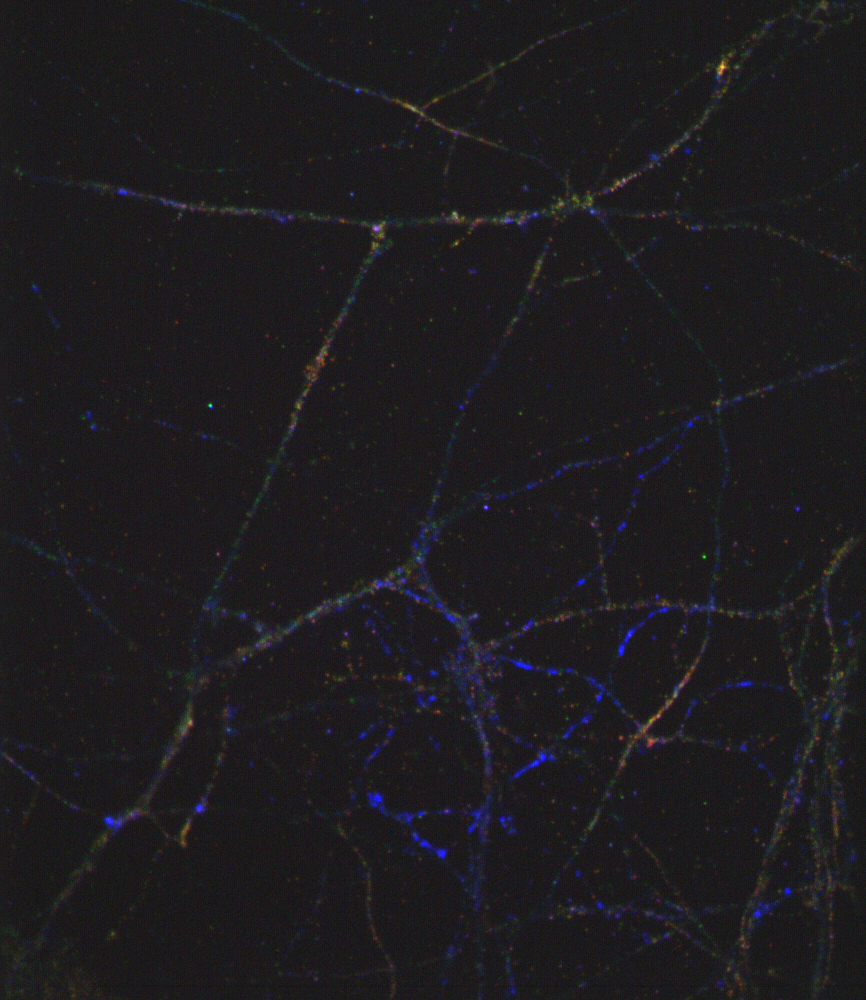

Supplement: Supplementary file 7 — Source Data for Figure 4 [file EMBJ-41-e109352-s003.zip › Figure_4_Source_Data+Statistics/Figure_4E-F/Figure_4F_Raw_Images/VPS34IN1_nonStim/34in_syt_surf(R)total(G)vGlut(B)_9.tif]

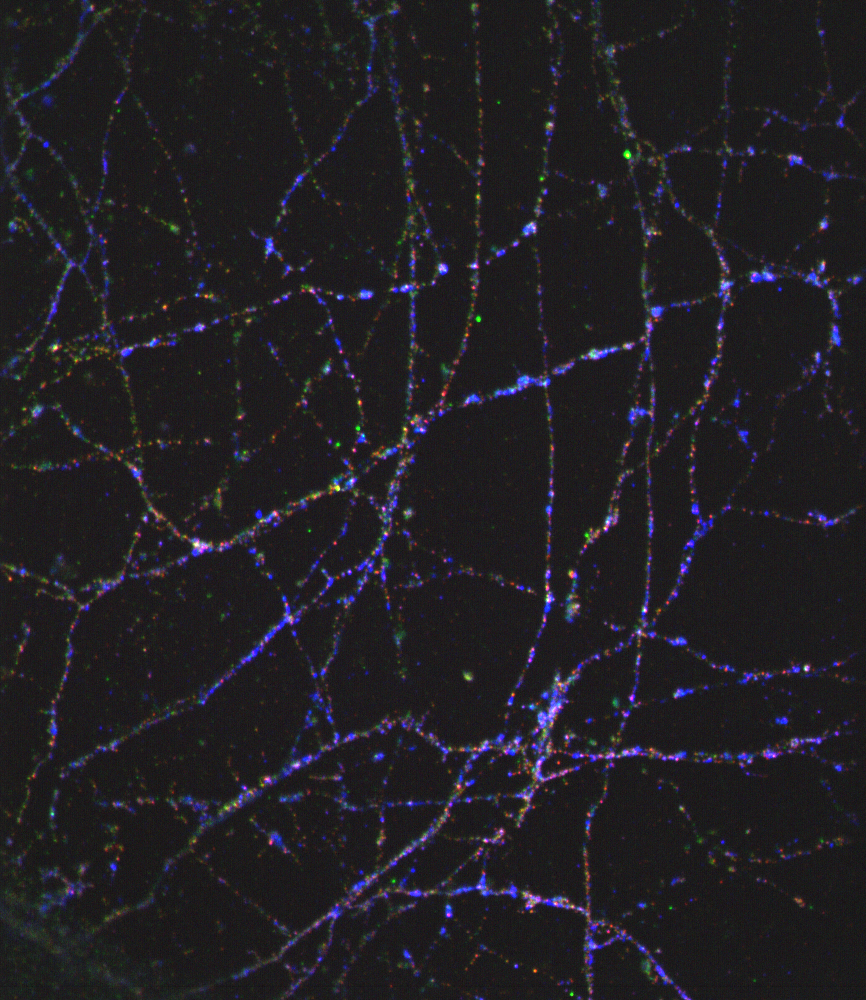

Supplement: Supplementary file 7 — Source Data for Figure 4 [file EMBJ-41-e109352-s003.zip › Figure_4_Source_Data+Statistics/Figure_4E-F/Figure_4F_Raw_Images/VPS34IN1_Stim/34in_stim_syt_surf(R)total(G)vGlut(B).tif]

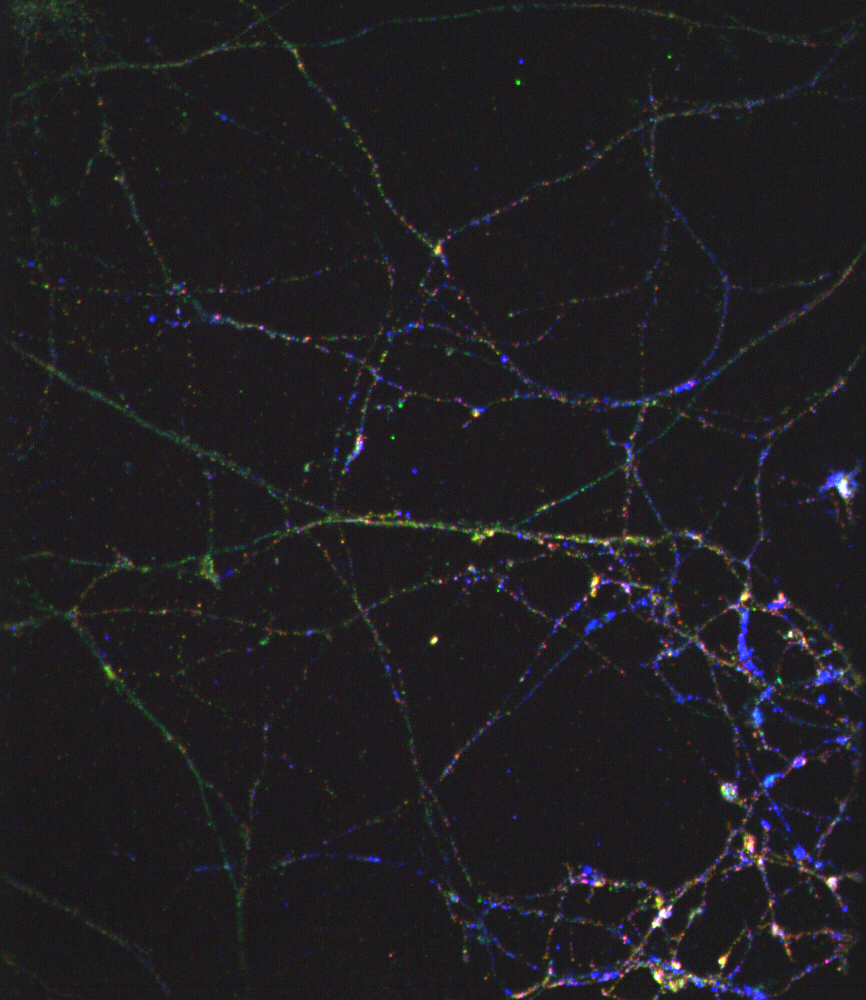

Supplement: Supplementary file 7 — Source Data for Figure 4 [file EMBJ-41-e109352-s003.zip › Figure_4_Source_Data+Statistics/Figure_4E-F/Figure_4F_Raw_Images/VPS34IN1_Stim/34in_stim_syt_surf(R)total(G)vGlut(B)_10.tif]

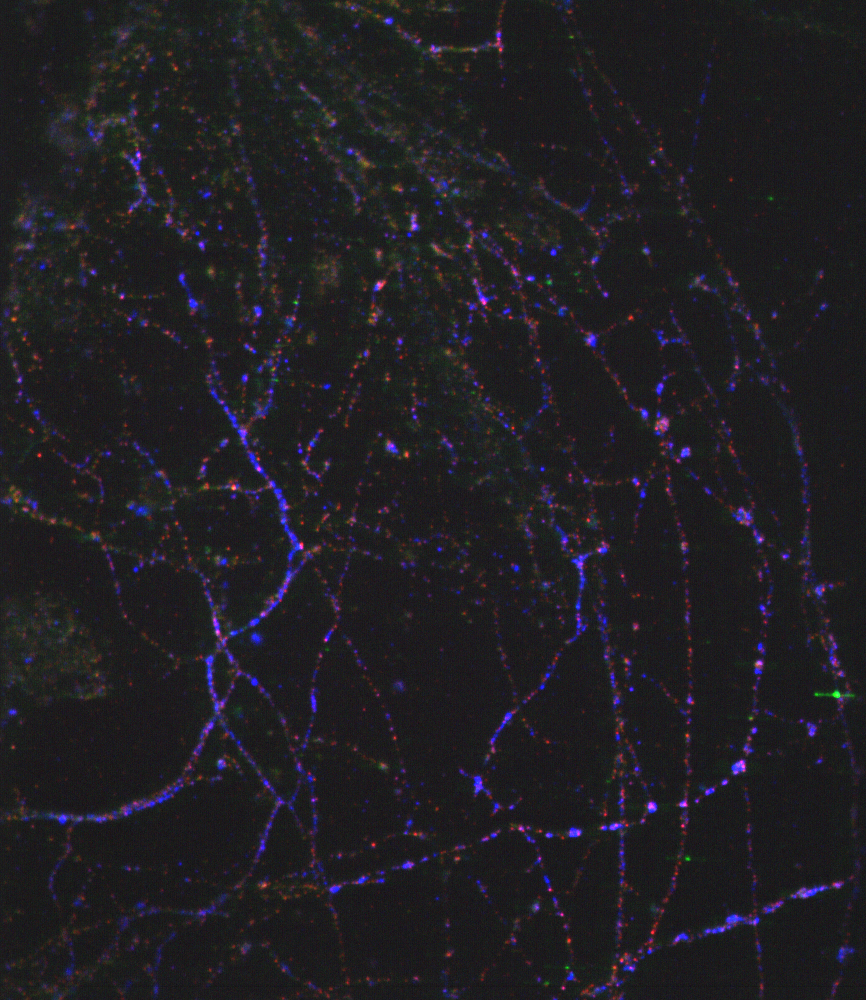

Supplement: Supplementary file 7 — Source Data for Figure 4 [file EMBJ-41-e109352-s003.zip › Figure_4_Source_Data+Statistics/Figure_4E-F/Figure_4F_Raw_Images/VPS34IN1_Stim/34in_stim_syt_surf(R)total(G)vGlut(B)_2.tif]

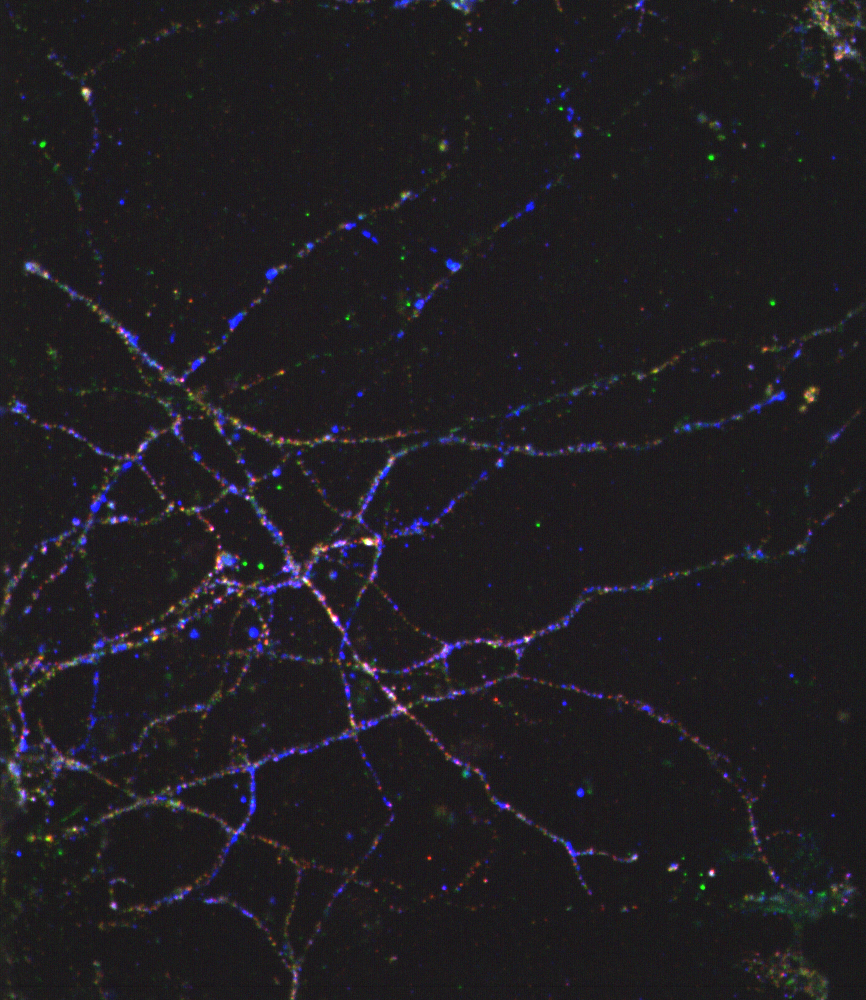

Supplement: Supplementary file 7 — Source Data for Figure 4 [file EMBJ-41-e109352-s003.zip › Figure_4_Source_Data+Statistics/Figure_4E-F/Figure_4F_Raw_Images/VPS34IN1_Stim/34in_stim_syt_surf(R)total(G)vGlut(B)_3.tif]

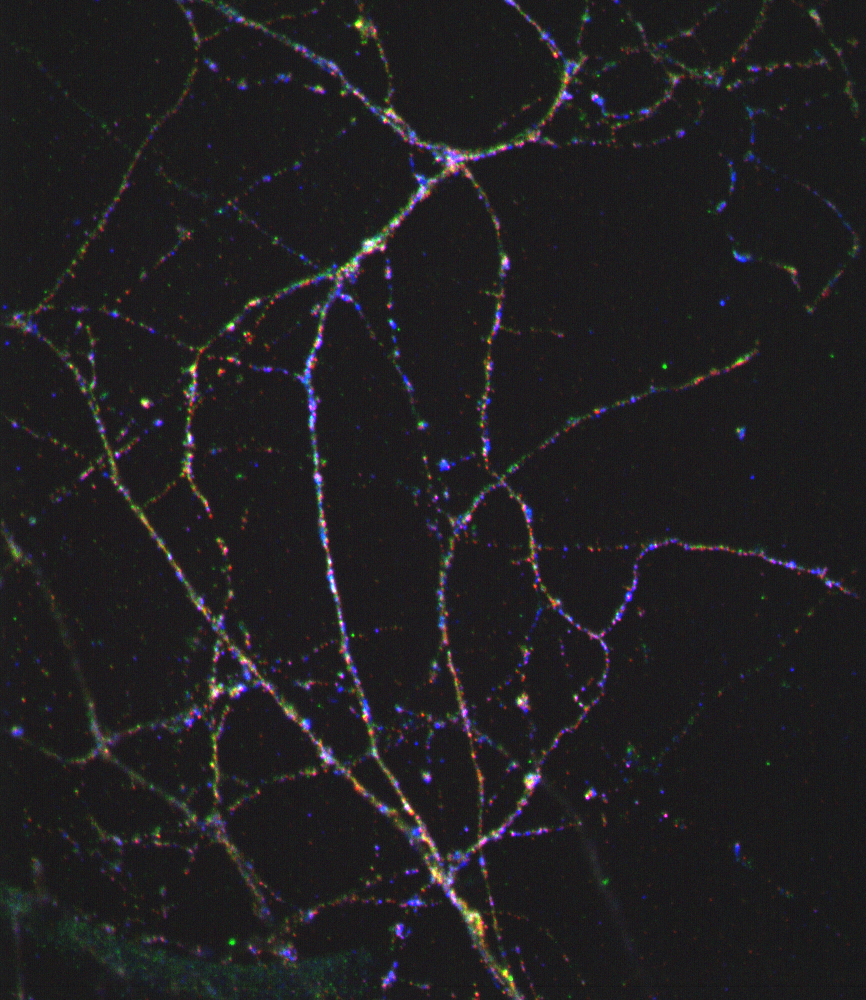

Supplement: Supplementary file 7 — Source Data for Figure 4 [file EMBJ-41-e109352-s003.zip › Figure_4_Source_Data+Statistics/Figure_4E-F/Figure_4F_Raw_Images/VPS34IN1_Stim/34in_stim_syt_surf(R)total(G)vGlut(B)_4.tif]

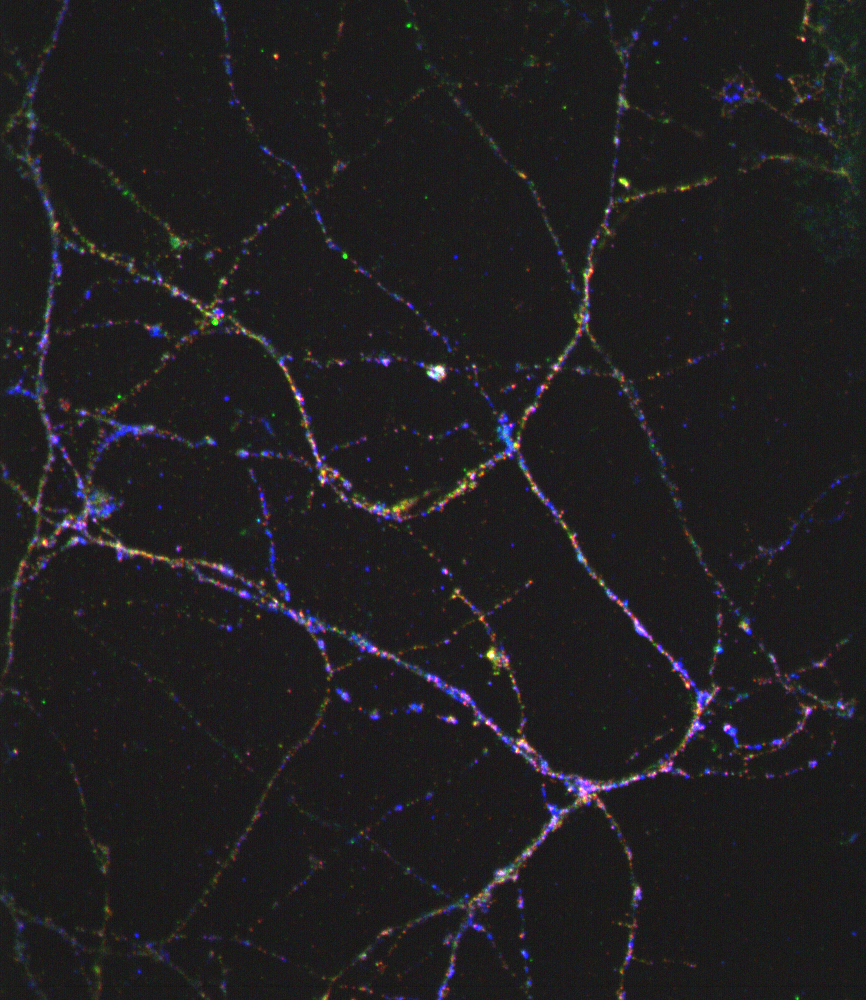

Supplement: Supplementary file 7 — Source Data for Figure 4 [file EMBJ-41-e109352-s003.zip › Figure_4_Source_Data+Statistics/Figure_4E-F/Figure_4F_Raw_Images/VPS34IN1_Stim/34in_stim_syt_surf(R)total(G)vGlut(B)_5.tif]

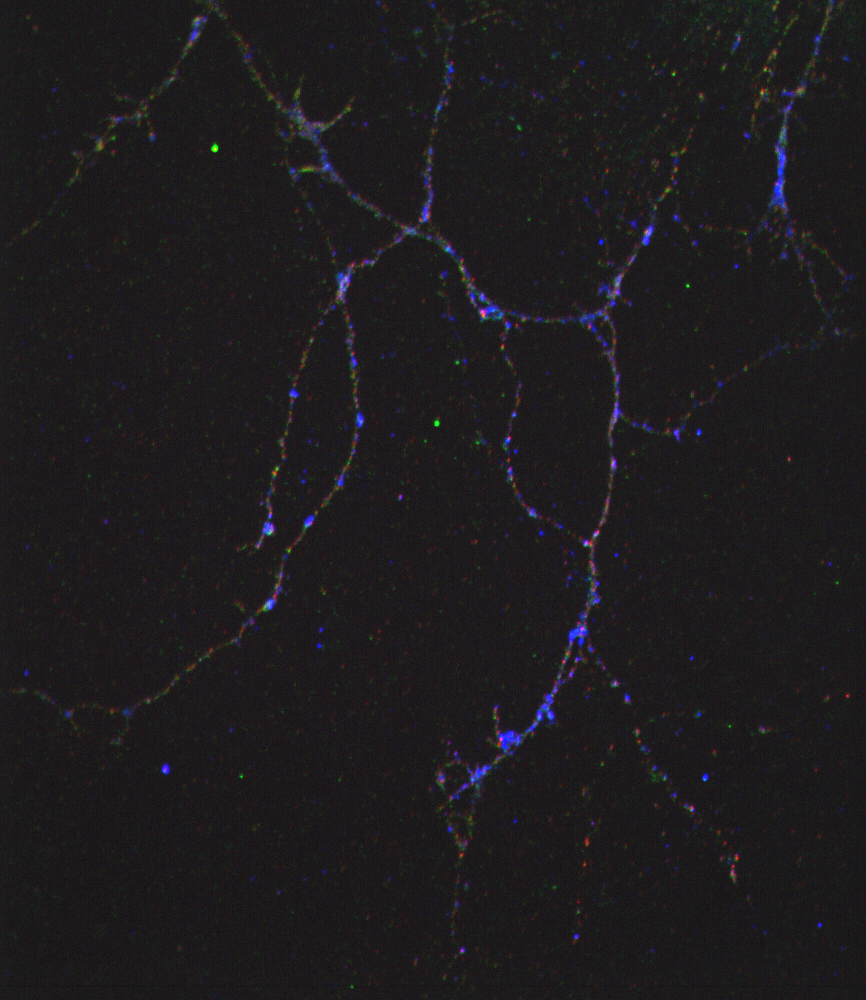

Supplement: Supplementary file 7 — Source Data for Figure 4 [file EMBJ-41-e109352-s003.zip › Figure_4_Source_Data+Statistics/Figure_4E-F/Figure_4F_Raw_Images/VPS34IN1_Stim/34in_stim_syt_surf(R)total(G)vGlut(B)_6.tif]

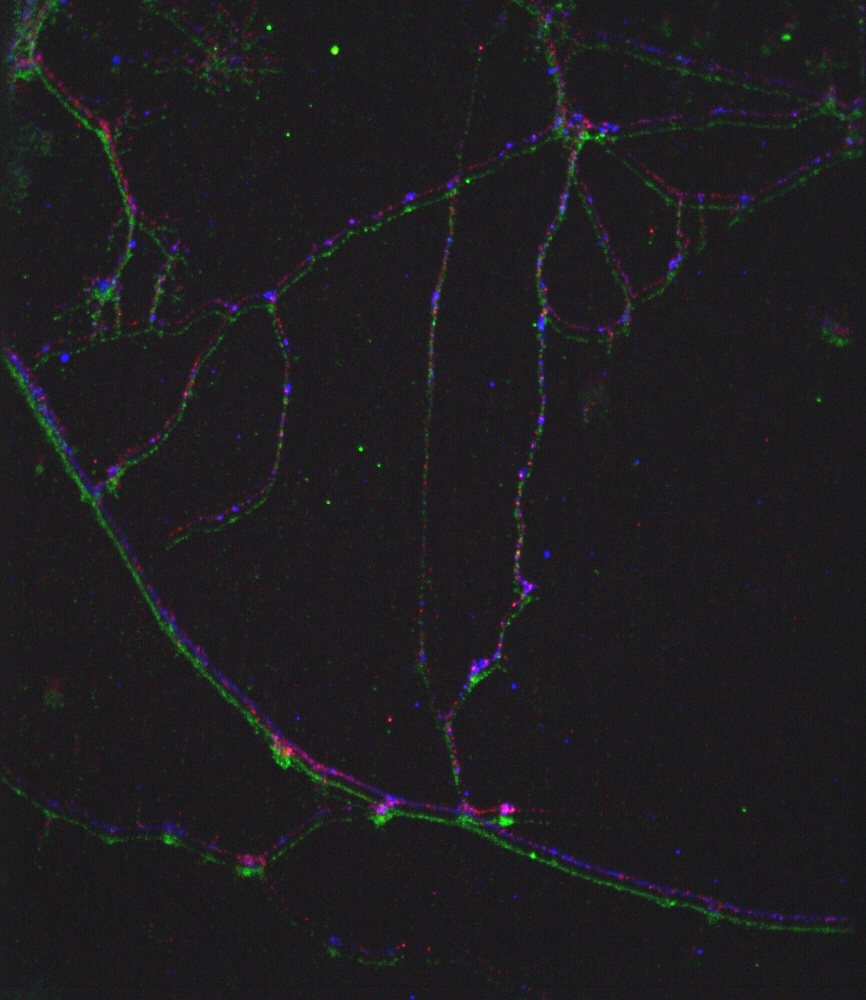

Supplement: Supplementary file 7 — Source Data for Figure 4 [file EMBJ-41-e109352-s003.zip › Figure_4_Source_Data+Statistics/Figure_4E-F/Figure_4F_Raw_Images/VPS34IN1_Stim/34in_stim_syt_surf(R)total(G)vGlut(B)_7.tif]

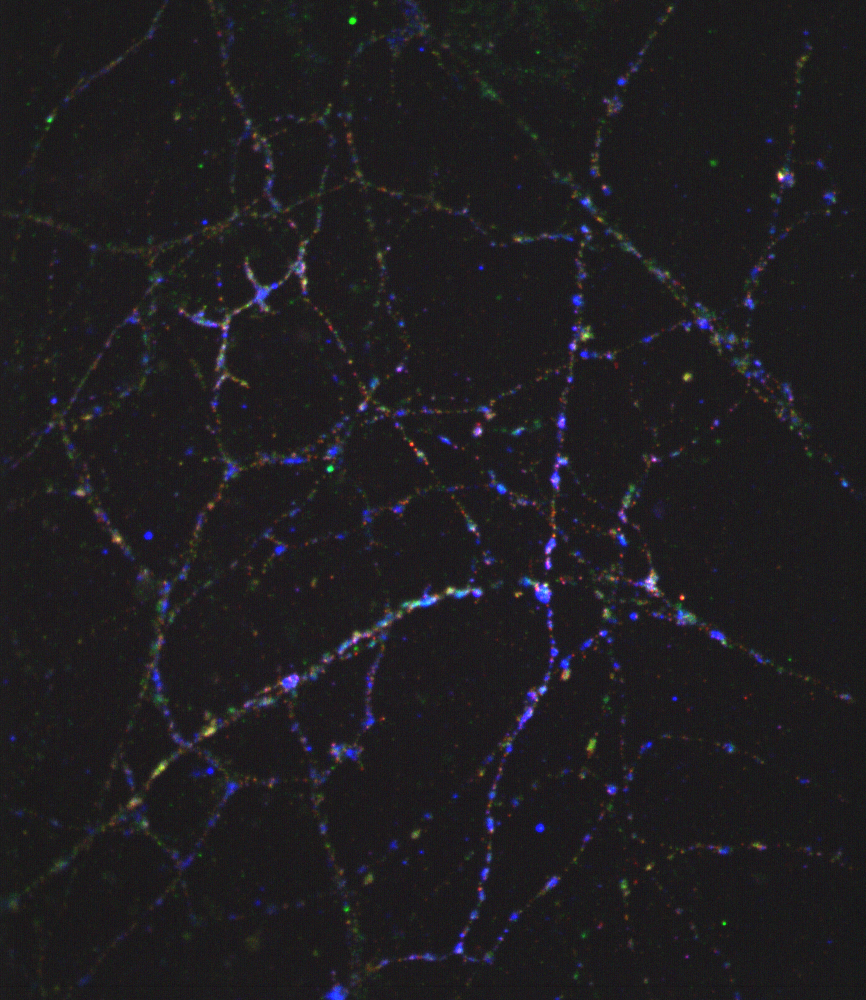

Supplement: Supplementary file 7 — Source Data for Figure 4 [file EMBJ-41-e109352-s003.zip › Figure_4_Source_Data+Statistics/Figure_4E-F/Figure_4F_Raw_Images/VPS34IN1_Stim/34in_stim_syt_surf(R)total(G)vGlut(B)_8.tif]

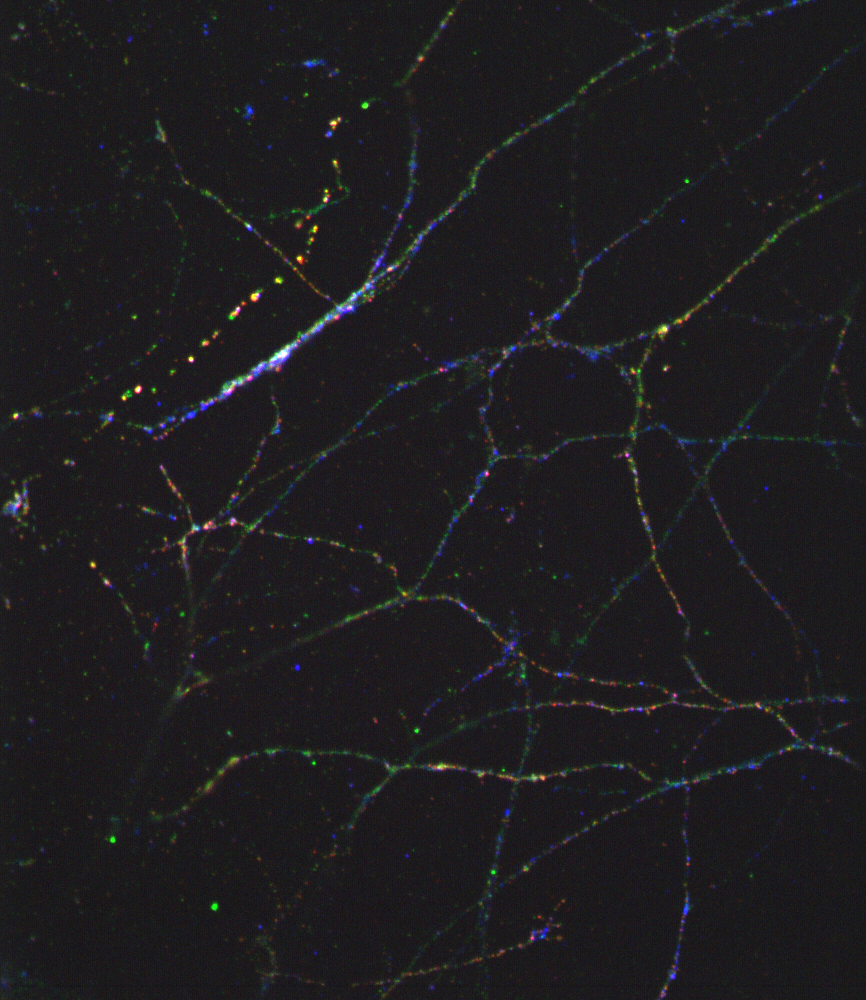

Supplement: Supplementary file 7 — Source Data for Figure 4 [file EMBJ-41-e109352-s003.zip › Figure_4_Source_Data+Statistics/Figure_4E-F/Figure_4F_Raw_Images/VPS34IN1_Stim/34in_stim_syt_surf(R)total(G)vGlut(B)_9.tif]

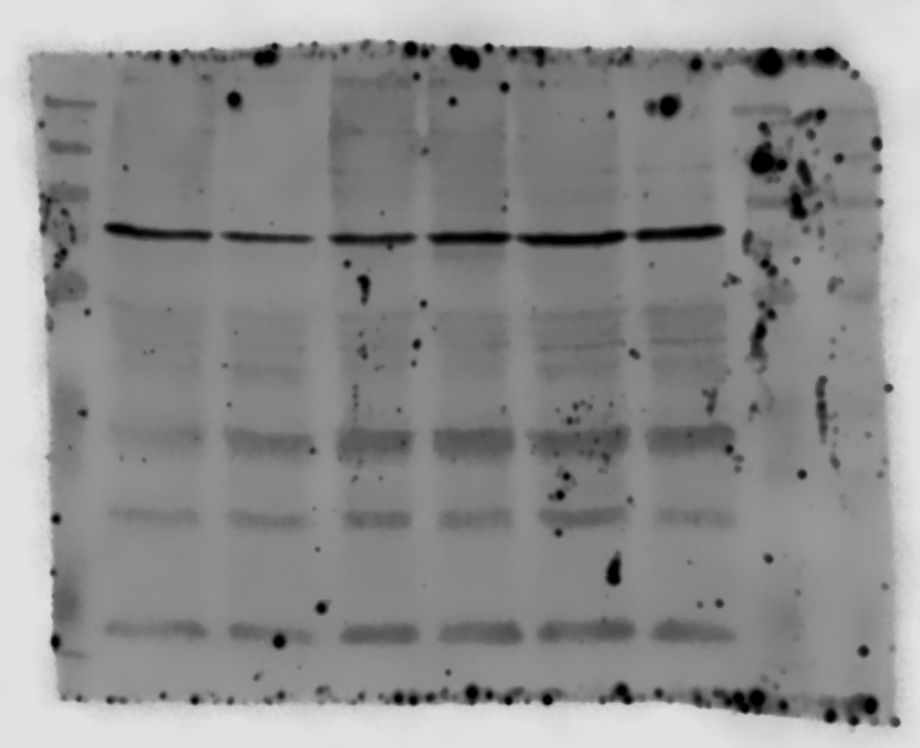

Supplement: Supplementary file 8 — Source Data for Figure 5 [file EMBJ-41-e109352-s008.zip › Figure_5_Source_Data+Statistics/Figure_5E/western_calpain2.tif]

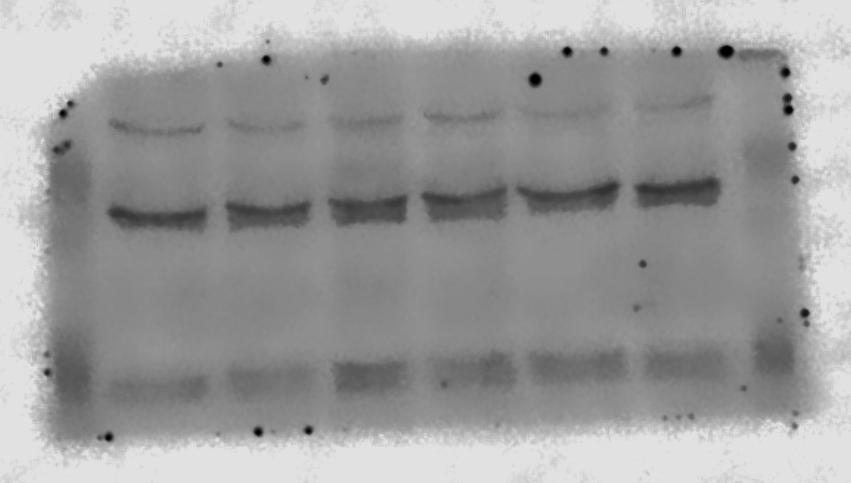

Supplement: Supplementary file 8 — Source Data for Figure 5 [file EMBJ-41-e109352-s008.zip › Figure_5_Source_Data+Statistics/Figure_5E/western_cdk5.tif]

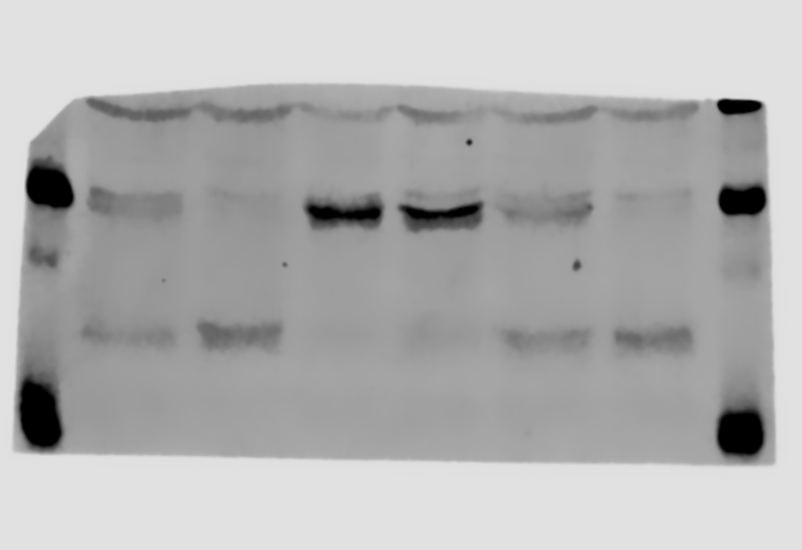

Supplement: Supplementary file 8 — Source Data for Figure 5 [file EMBJ-41-e109352-s008.zip › Figure_5_Source_Data+Statistics/Figure_5E/western_p35-p25.tif]

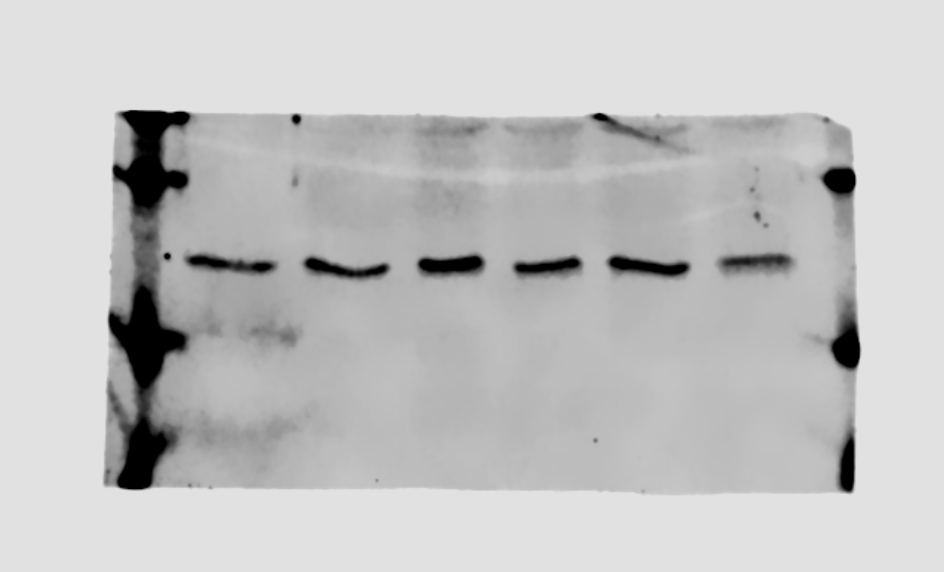

Supplement: Supplementary file 8 — Source Data for Figure 5 [file EMBJ-41-e109352-s008.zip › Figure_5_Source_Data+Statistics/Figure_5E/western_rab5.tif]

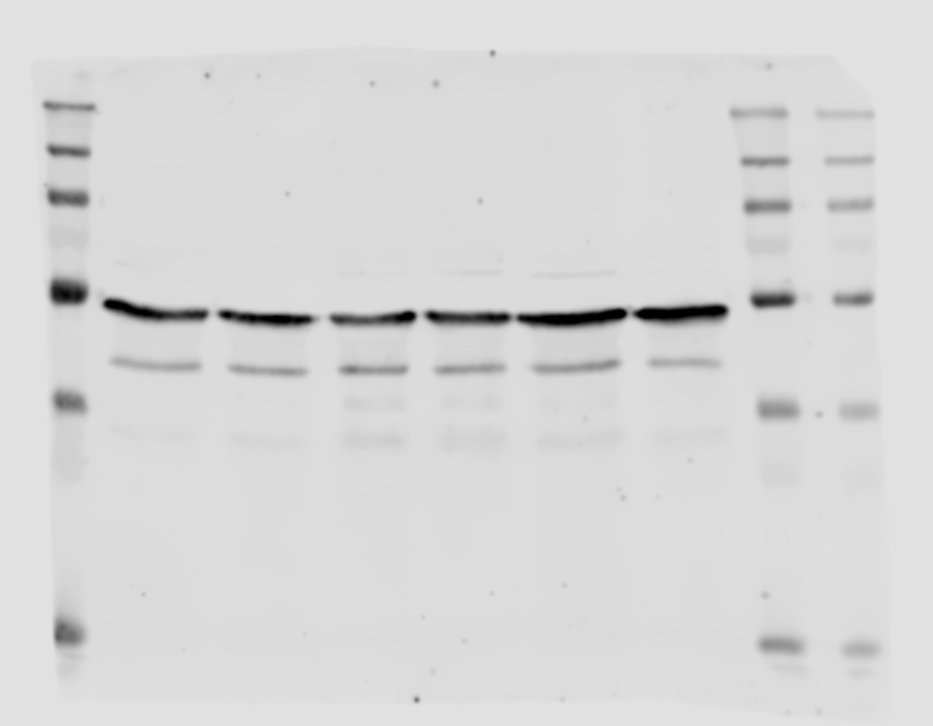

Supplement: Supplementary file 8 — Source Data for Figure 5 [file EMBJ-41-e109352-s008.zip › Figure_5_Source_Data+Statistics/Figure_5E/western_tubulin.tif]
