# Supplementary material for: Endosomal phosphatidylinositol 3‐phosphate controls synaptic vesicle cycling and neurotransmission
Source: EMBO J. 2022 Mar 22;41(9):e109352. doi: 10.15252/embj.2021109352 (PMC9058544; doi:10.15252/embj.2021109352)
Supplement: Supplementary file 11 [file EMBJ-41-e109352-s007.pdf]

THE EMBO JOURNAL

Endosomal phosphatidylinositol 3-phosphate controls synaptic vesicle cycling and neurotransmission

Guan-Ting Liu, Gaga Kochlamazashvili, Dmytro Puchkov, Rainer Mueller, Carsten Schultz, Albert Mackintosh, Dennis Vollweiter, Volker Haucke, and Tolga Soykan
DOI: 10.15252/embj.2021109352

Corresponding author(s): Volker Haucke (haucke@fmp-berlin.de) , Tolga Soykan (soykan@fmp-berlin.de)

Review Timeline:

Submission Date:	2nd Aug 21
Editorial Decision:	7th Sep 21
Revision Received:	24th Jan 22
Editorial Decision:	23rd Feb 22
Revision Received:	24th Feb 22
Accepted:	1st Mar 22

Editor: Karin Dumstrei

Transaction Report:

(Note: With the exception of the correction of typographical or spelling errors that could be a source of ambiguity, letters and reports are not edited. Depending on transfer agreements, referee reports obtained elsewhere may or may not be included in this compilation. Referee reports are anonymous unless the Referee chooses to sign their reports.)

1st Editorial Decision 7th Sep 2021

Dear Volker,

Thank you for submitting your manuscript to The EMBO Journal. Your study has now been seen by three referees and their comments are provided below.

As you can see the referees find the analysis interesting and are supportive of publication here. They raise different concerns that I anticipate that you should be able to address in a good way. I would therefore like to invite you to submit a revised version.

I think it would be helpful to discuss the raised points further and I am available to do so via email or video.

When preparing your letter of response to the referees' comments, please bear in mind that this will form part of the Review Process File, and will therefore be available online to the community. For more details on our Transparent Editorial Process, please visit our website: https://www.embopress.org/page/journal/14602075/authorguide#transparentprocess

I have attached a document with helpful tips on how to prepare the revised version. Please pay attention to the parts on the Data Availability Section and figure legends.

Thank you for the opportunity to consider your work for publication. I look forward to discussing your revisions further with you.

with best wishes

Karin

Karin Dumstrei, PhD
Senior Editor
The EMBO Journal

Guide For Authors: https://www.embopress.org/page/journal/14602075/authorguide

Revision to The EMBO Journal should be submitted online within 90 days, unless an extension has been requested and approved by the editor; please click on the link below to submit the revision online before 6th Dec 2021:

https://emboj.msubmit.net/cgi-bin/main.plex

Referee #1:

- General summary and opinion about the principal significance of the study

Liu et al. investigated the role of endosomal lipid PI(3)P in regulating SV recycling and presynaptic neurotransmission. They made several interesting findings including: endosomal PI(3)P was repressed by neuronal activity via Cdk5-dependent regulation of the lipid kinase VPS34; pharmacological inhibition of VPS34 disrupted SV recycling and neurotransmission; PI(3)P depletion impaired SV endocytosis and synaptic transmission, via likely Calpain 2-mediated hyperactivation of Cdk5; and, endosomal PI(3)P depletion restricted network activity by repressing excitatory neurotransmission, independent of the effects of synaptic inhibition. These results suggest an autoregulatory pathway through the regulation of the endosomal lipid PI(3)P to control neurotransmitter release and synaptic vesicle cycling. The findings in this study are interesting and significant as they connect endosomal signaling and synaptic activity.

- Specific major concerns essential to be addressed to support the conclusions

The interpretation of Figure 6D-E and Figure EV5 needs to be clarified. In Figure 6D-E, DMSO treatments are shown as controls. However, in this reviewer's opinion, the correct control for the VPS34IN1 + Roscovitine treatments should be Roscovitine alone, not DMSO. The way that these data are presented in the manuscript appears to be somewhat misleading. This is an important issue as it could change the conclusion regarding the role of CDK5 in synaptic changes induced by PI(3)P depletion. A "complete rescue" should bring synaptic activity back to the level of Roscovitine treatment alone, not that seen in the DMSO treatment. The interpretation of Figure 6D-E and Figure EV5 affects the following text.

On page 8 - "Pharmacological blockade of Cdk5 by the specific inhibitor Roscovitine (Shah & Lahiri, 2014; Tan et al., 2003) indeed fully rescued defective SV endocytosis under conditions of PI(3)P loss (Fig 6B)."

On page 10 - "The fact that acute inhibition of Cdk5 activity completely rescues defects in SV endocytosis during multiple trains of APs in the near complete absence of PI(3)P further indicates that PI(3)P-containing endosomes are unlikely to be absolutely essential for SV recycling in excitatory hippocampal neurons."

- Minor concerns that should be addressed

How does PI(3)P depletion affect PIs conversion? While VPS34IN1 is a specific blocker, PI(3)P depletion could lead to changes of the synthesis and turnover of other PIs. Have authors looked at other PIs such as PI(3,5)P2, PI(3,4)P2, and PI(4)P and determined if they were affected by PI(3)P depletion? If so, it may be helpful for the readers to see them.

Referee #2:

This study submitted by Liu et al. claims that phosphatidylinositol 3-phosphate (PI3P), a critical component for endosome function, controls synaptic vesicle recycling and synaptic transmission and regulating synaptic physiology. The authors examined that neuronal activity decreased the level of PI3P via VPS34s, consequently Decreased PI3P suppress neurotransmission eventually disrupts synaptic vesicle endocytosis. Finally, this process is mediated by Calpain2 dependent CDK5 activity, suggesting that endosomal PI3P regulates synaptic function (e.g., synaptic transmission and synaptic vesicle cycling) via activity-dependent calpain2-CDK5, which is an intrinsic autoregulatory system in the nervous system.

It is an interesting story. Particularly the authors have intensively explored with numerous experiments how endosomal PI3P is involved in synaptic vesicle trafficking at synapse, which is not much explored area in this field. However, some of the results seem not to fit the authors' model in this study; I have some major concerns which are the following.

1. PI3P participates in various internal membrane traffickings such as autophagy and various endosomal traffickings as the authors mentioned in the section of the introduction. I am wondering whether the phenotype in this study is a synaptic specific or indirect effect of PI3P in other pathways. Does VPS34 specifically or highly express in synapse? Or something else?
2. The authors showed dramatic reduction (more than 50%) of PI3P level after trains of activation without any disruption of VPS34 in fig1 and eventually suggested the model in fig7E (fig 6A also). I wonder why the results of control in this study that have utilized the trains of neural activation (fig2,4,5,6) do not merely show PI3P deficient defect. If this model is true, the trains of activity in normal synapses should show defects in synaptic transmission and endocytosis as activity goes by. Of course, the defect in control might be less than one drug-treated though.
3. The authors use surface retention as a useful phenotype. I am curious whether this surface retention is reversible. Sometimes, synapse can just show defective phenotype or dying (it gets bright) in the pHluorin based assay when it was treated drugs with strong or trains of stimulation. It would be good to show the surface retention change upon VPS34 activity or PIP level change in one cell.
4. Although the authors refer to the paper for the PI3P leveling experiment, adding more description in detail would help to understand how PI3P is leveled. How purified GFP-FYVE protein can get ENDOSMAL PI3P in the cytosol of cells after treatment? Is it endocytosed?

Referee #3:

In this study, Liu et al. investigated the relationship between neuronal endosomes, endosomal lipid phosphatidylinositol-3-phosphate (PI(3)P), and synaptic activity. They found that high neuronal activity decreases synthesis of PI(3)P by repressing activity of the main PI(3)P-synthesizing lipid kinase, VPS34 (also known as class III PI-3-kinase). They also found evidence for the converse, namely that neuronal activity itself is regulated by PI(3)P. Importantly depletion of PI(3)P achieved by pharmacological inhibition of Vps34 impairs neurotransmission due to negative pre-synaptic effects on synaptic vesicle recycling. Mechanistically, the authors determined that Vps34 inhibition, and thus, PI(3)P depletion, triggers calpain 2-mediated proteolytic processing of p35 to p25, which leads to hyperactivation of Cdk5 downstream of calcium influx.

This is an exciting study that reveals an unexpected role for PI(3)P-containing neuronal endosomes in regulation of neurotransmission, which has also important implications for neurological diseases, including neurodegenerative diseases. Vps34 and its product have primarily been studied in the context of endolysosomal function as well as autophagy, although most of these studies have focused on non-neuronal cells, with a few exceptions. This study is the first to take a deep dive into the role of neuronal PI(3)P in synaptic vesicle recycling and more generally, synaptic transmission. The authors make great use of various pharmacological tools, combined with powerful imaging techniques, electrophysiology and biochemistry to discovery and characterize this new pathway involving Vps34, calpain 2, p35 and Cdk5 (with feedback loop onto Vps34 itself). Overall, this is a strong candidate manuscript for this journal and there are only a few points that need to be addressed.

Main points:

1. As the authors are fully aware, phosphoinositide metabolism is largely regulated by lipid kinases and phosphatases. Can the authors rule out contributions of PI(3)P phosphatases (e.g. myotubularins, Synaptojanin 1's Sac1 domain) for the activity-dependent depletion of PI(3)P in neurons?

2. Can the authors confirm the role of Cdk5 in turning down Vps34 activity through reagents other than roscovitine? There are known off-target effects with this drug, so it is important to confirm these results with at least one alternative approach, namely, an independent pharmacological inhibitor/chemotype, a Cdk5 knockdown, or expression of a kinase-dead Cdk5 construct

3. The processing of p35 into p25 by calpain 2 seems very striking. Can the authors provide more direct biochemical evidence that this processing induced by PI(3)P depletion increases the kinase activity of Cdk5, by looking either at the kinase activity itself or direct Cdk5 substrates? Additionally, is this processing only observed in cerebellar neurons or can it be shown also in hippocampal/cortical neurons?

Other comments:

4. Morel et al. is cited as evidence for a role of PI(3)P in non-neuronal cells in the introduction, but this paper, as well as a follow-up study from the same group (Miranda et al. PMID: 29348617), explored the role of Vps34, and thus PI(3)P, in neurons both in vitro (both papers) and in vivo (only Miranda et al.), but not necessarily at synapses. They should probably be cited in the appropriate contexts. Additionally, the Miranda paper demonstrates a key role for neuronal PI(3)P in endolysosomal function as well as autophagy, which should perhaps also be mentioned in this manuscript.

5. In Figure EV1-C-D, the panels should indicate "Merge" for the merged channels

6. On page 4, it would be helpful to mention that Cdk5 controls Vps34 via phosphorylation of Thr159, when citing the Furuya et al. 2010 paper.

7. Since Cdk5 activity has been heavily implicated in Alzheimer's disease (AD) in part due to its role in tau hyperphosphorylation, is it conceivable that the PI(3)P deficiency observed in AD patients' brrains may also impact tau phosphorylation through hyperactivation of Cdk5? It is perhaps worth discussing this point in the Discussion, when thinking about potential implications for neurological diseases.

Response to the reviewers

We would like to thank the Editor as well as all three referees for their careful reading of our manuscript, for their highly constructive comments and questions. We were very happy to learn that all three reviewers agree on the high general interest and timeliness of our study and its suitability for publication in *The EMBO J*. In the revised manuscript we have addressed *their questions and concerns* as further detailed below (our response in blue).

Responses to reviewer #1:

Liu et al. investigated the role of endosomal lipid PI(3)P in regulating SV recycling and presynaptic neurotransmission. They made several interesting findings including: endosomal PI(3)P was repressed by neuronal activity via Cdk5-dependent regulation of the lipid kinase VPS34; pharmacological inhibition of VPS34 disrupted SV recycling and neurotransmission; PI(3)P depletion impaired SV endocytosis and synaptic transmission, via likely Calpain 2-mediated hyperactivation of Cdk5; and, endosomal PI(3)P depletion restricted network activity by repressing excitatory neurotransmission, independent of the effects of synaptic inhibition. These results suggest an autoregulatory pathway through the regulation of the endosomal lipid PI(3)P to control neurotransmitter release and synaptic vesicle cycling. The findings in this study are interesting and significant as they connect endosomal signaling and synaptic activity.

Response: We thank reviewer 1 for this lucid summary and for highlighting the general interest and significance of our study.

The interpretation of Figure 6D-E and Figure EV5 needs to be clarified. In Figure 6D-E, DMSO treatments are shown as controls. However, in this reviewer's opinion, the correct control for the VPS34IN1 + Roscovitine treatments should be Roscovitine alone, not DMSO. The way that these data are presented in the manuscript appears to be somewhat misleading. This is an important issue as it could change the conclusion regarding the role of CDK5 in synaptic changes induced by PI(3)P depletion. A "complete rescue" should bring synaptic activity back to the level of Roscovitine treatment alone, not that seen in the DMSO treatment. The interpretation of Figure 6D-E and Figure EV5 affects the following text.

Response: We agree that the previous arrangement of figures did not allow for an easy comparison of fEPSP responses in the presence of either Roscovitine alone (former EV5) or VPS34IN1 + Roscovitine. The purpose of these experiments was to find out whether Cdk5 inhibition by Roscovitine can rescue the rundown of fEPSP responses induced by depletion of PI(3)P in the presence of VPS34IN1 shown in Fig 2A,B. To allow for a better comparison between these conditions, we have now followed the suggestion of the referee to combine former figure panels 6D-E and the former Fig EV5 into a single **new Figure 6D,E**. Given that both measurements were carried out independently, the corresponding controls are indicated separately (with no distinguishable differences between different controls).

With respect to these experiments, we now state in the revised version of our manuscript on p. 8: *"Blockade of Cdk5 activity by Roscovitine largely occludes the adverse effects of VPS34IN1 on excitatory neurotransmission in acute hippocampal slice preparations. Instead, fEPSPs were facilitated in the presence of Roscovitine. This facilitatory effect of Roscovitine was similar albeit less pronounced than that seen in recordings from Roscovitine-treated control slices lacking*

VPS34IN1 (Fig 6D). Furthermore, Roscovitine reverted the increase in paired pulse ratios induced by VPS34IN1 alone (Fig 2B) and, instead, caused paired-pulse depression (Figs 6E)."

The slightly reduced facilitation of fEPSP responses in the presence of both VPS34IN1 + Roscovitine compared to Roscovitine alone suggests that apart from Cdk5 activation, Vps34IN1 may have some additional adverse effect on neurotransmission that could either be due to additional targets of PI(3)P apart from Calpain/ Cdk5 or may be due to low level side effects of drug application.

On page 8 - "Pharmacological blockade of Cdk5 by the specific inhibitor Roscovitine (Shah & Lahiri, 2014; Tan et al., 2003) indeed fully rescued defective SV endocytosis under conditions of PI(3)P loss (Fig 6B)."

On page 10 - "The fact that acute inhibition of Cdk5 activity completely rescues defects in SV endocytosis during multiple trains of APs in the near complete absence of PI(3)P further indicates that PI(3)P-containing endosomes are unlikely to be absolutely essential for SV recycling in excitatory hippocampal neurons."

Response: The two quoted statements refer to the rescue of defective Synaptophysin-pHluorin endocytosis seen upon PI(3)P depletion by application of Roscovitine. As shown in **Fig 6B** Roscovitine indeed completely rescued the delayed endocytosis kinetics induced by VPS34IN1 as correctly stated on p.8 of the original manuscript. To make clear that the quoted statement on p.10 refers to these data we have amended the main manuscript text as follows:

"The fact that acute inhibition of Cdk5 activity rescues defects in synaptophysin-pHluorin endocytosis during multiple trains of APs (compare Fig 6B) in the near complete absence of PI(3)P further indicates that PI(3)P-containing endosomes are unlikely to be absolutely essential for SV recycling in excitatory hippocampal neurons."

We apologize for not having this made clear in the original version of our manuscript.

Minor concerns:
How does PI(3)P depletion affect PIs conversion? While VPS34IN1 is a specific blocker, PI(3)P depletion could lead to changes of the synthesis and turnover of other PIs. Have authors looked at other PIs such as PI(3,5)P2, PI(3,4)P2, and PI(4)P and determined if they were affected by PI(3)P depletion? If so, it may be helpful for the readers to see them.

Response: We agree that the analysis of other phosphoinositides is an interesting endeavor for future studies. As shown in our original paper we did not detect any overt effects of VPS34IN1 on the levels of other phosphoinositide species (Ketel et al Nature 2016). To satisfy the referee, we have also conducted additional experiments to probe the effects of stimulation on presynaptic phosphatidylinositol 4,5-bisphosphate [PI(4,5)P_2] levels. As now stated in our revised manuscript on p.4, we confirmed the previously observed stimulation-induced elevation of presynaptic PI(4,5)P_2 at active synapses (Micheva et al J Cell Biol 2001).

Responses to reviewer #2:

This study submitted by Liu et al. claims that phosphatidylinositol 3-phosphate (PI3P), a critical component for endosome function, controls synaptic vesicle recycling and synaptic transmission and regulating synaptic physiology. The authors examined that neuronal activity decreased the level of PI3P via VPS34s, consequently Decreased PI3P suppress neurotransmission eventually disrupts synaptic vesicle endocytosis. Finally, this process is mediated by Calpain2 dependent CDK5 activity, suggesting that endosomal PI3P regulates synaptic function (e.g., synaptic transmission and synaptic vesicle cycling) via activity-dependent calpain2-CDK5, which is an intrinsic autoregulatory system in the nervous system.

It is an interesting story. Particularly the authors have intensively explored with numerous experiments how endosomal PI3P is involved in synaptic vesicle trafficking at synapse, which is not much explored area in this field.

Response: We thank reviewer 2 for the summary of our findings and for his/ her very positive assessment as well as for highlighting the general interest of our study.

However, some of the results seem not to fit the authors' model in this study; I have some major concerns which are the following.

1. PI3P participates in various internal membrane traffickings such as autophagy and various endosomal traffickings as the authors mentioned in the section of the introduction. I am wondering whether the phenotype in this study is a synaptic specific or indirect effect of PI3P in other pathways. Does VPS34 specifically or highly express in synapse? Or something else?

Response: We thank the referee for raising this interesting point. We show that the stimulation-induced depletion of PI(3)P occurs both at excitatory and inhibitory synapses and in neuronal somata with equal efficiency (Fig 1 + EV1). Our recombinant probe for PI(3)P further suggests that while all neuronal somata contain PI(3)P-positive endosomes, this is not the case for all synapses (although most synapses display some PI(3)P). Together with our observation that the effects of VPS34 inhibition are fully rescued by NMDA receptor inhibition (**see Fig 5C, AP5**) we favor a model whereby both somatic and synaptic pools of VPS34 impact on neurotransmission. As currently no tools exist that allow monitoring of the distribution of the endogenous VPS34 kinase complex in neurons, we hope to address this interesting question in future studies in more detail.

2. The authors showed dramatic reduction (more than 50%) of PI3P level after trains of activation without any disruption of VPS34 in fig1 and eventually suggested the model in fig7E (fig 6A also). I wonder why the results of control in this study that have utilized the trains of neural activation (fig2,4,5,6) do not merely show PI3P deficient defect. If this model is true, the trains of activity in normal synapses should show defects in synaptic transmission and endocytosis as activity goes by. Of course, the defect in control might be less than one drug-treated though.

Response: We thank the referee for this interesting question and suggestion that we have addressed experimentally as follows. We have designed a SynaptopHluorin-based assay to monitor SV exocytosis in response to 10 AP stimulation under conditions of PI(3)P depletion following trains of 200 AP stimulations. These experiments displayed in the **new Extended View Figure EV4A-B** reveal a significant downregulation of the exocytic responses following

train stimulation. Importantly, this effect is rescued by Cdk5 inhibition, further supporting the view that PI(3)P depletion causes suppression of presynaptic neurotransmission.

3. The authors use surface retention as a useful phenotype. I am curious whether this surface retention is reversible. Sometimes, synapse can just show defective phenotype or dying (it gets bright) in the pHluorin based assay when it was treated drugs with strong or trains of stimulation. It would be good to show the surface retention change upon VPS34 activity or PIP level change in one cell.

Response: This is an important point: To test whether PI(3)P depletion on SV endocytosis is reversible, we had originally performed a washout experiment, in which VPS34IN1 was removed after 1h treatment and the neurons were left to recover for 24h (**Fig EV2C in the current version**). This washout period was sufficient to completely revert the effects of VPS34IN1 on surface retention of synaptophysin-pHluorin, indicating that the phenotype is specific and not caused by irreversible damage to the neurons. This conclusion is further underscored by the ability of a variety of other manipulations, e.g. AP5, ALLN, Roscovitine, or calpain 2 knockdown to occlude defective SV endocytosis in the presence of VPS34IN1.

4. Although the authors refer to the paper for the PI3P leveling experiment, adding more description in detail would help to understand how PI3P is leveled. How purified GFP-FYVE protein can get ENDOSMAL PI3P in the cytosol of cells after treatment? Is it endocytosed?

Response: We use purified recombinant eGFP-2xFYVE protein as a probe to detect PI(3)P in fixed Digitonin-permeabilized neurons as shown in previous studies by us (e.g. Marat et al., Science 2017; Ketel et al Nature 2016) and others. The fluorescent signal from the PI(3)P-bound probe is further enhanced by treatment with a primary GFP and Alexa Fluor 488 conjugated secondary antibodies.

Responses to reviewer #3:
In this study, Liu et al. investigated the relationship between neuronal endosomes, endosomal lipid phosphatidylinositol-3-phosphate (PI(3)P), and synaptic activity. They found that high neuronal activity decreases synthesis of PI(3)P by repressing activity of the main PI(3)P-synthesizing lipid kinase, VPS34 (also known as class III PI-3-kinase). They also found evidence for the converse, namely that neuronal activity itself is regulated by PI(3)P. Importantly depletion of PI(3)P achieved by pharmacological inhibition of Vps34 impairs neurotransmission due to negative pre-synaptic effects on synaptic vesicle recycling. Mechanistically, the authors determined that Vps34 inhibition, and thus, PI(3)P depletion, triggers calpain 2-mediated proteolytic processing of p35 to p25, which leads to hyperactivation of Cdk5 downstream of calcium influx.

This is an exciting study that reveals an unexpected role for PI(3)P-containing neuronal endosomes in regulation of neurotransmission, which has also important implications for neurological diseases, including neurodegenerative diseases. Vps34 and its product have primarily been studied in the context of endolysosomal function as well as autophagy, although most of these studies have focused on non-neuronal cells, with a few exceptions. This study is the first to take a deep dive into the role of neuronal PI(3)P in synaptic vesicle recycling and more generally, synaptic transmission. The authors make great use of various pharmacological tools, combined with powerful imaging techniques, electrophysiology and biochemistry to discovery and characterize this new pathway involving Vps34, calpain 2, p35 and Cdk5 (with feedback loop onto Vps34 itself). Overall, this is a strong candidate manuscript for this journal and there are only a few points that need to be addressed.

Response: We thank reviewer 3 for the nice summary of our findings and for his/ her very positive assessment as well as for highlighting the general interest and suitability of our study to be published in *The EMBO J*.

Main points:
1. As the authors are fully aware, phosphoinositide metabolism is largely regulated by lipid kinases and phosphatases. Can the authors rule out contributions of PI(3)P phosphatases (e.g. myotubularins, Synaptojanin 1's Sac1 domain) for the activity-dependent depletion of PI(3)P in neurons?

Response: We cannot fully exclude the contributions of PI(3)P phosphatases to the observed PI(3)P regulation by neuronal activity (now acknowledged in the discussion on **p. 10** of the revised manuscript). That said, we have conducted additional experiments to further probe the contribution of Cdk5-mediated phosphorylation of VPS34 to the activity-dependent regulation of neuronal PI(3)P levels. To this aim, we have generated a non-phosphorylatable mutant (T159A) of VPS34 kinase that cannot be targeted by Cdk5. We then analyzed the levels of PI(3)P in Gabazine-treated neurons expressing either WT or non-phosphorylatable mutant (T159A) VPS34. Gabazine-dependent PI(3)P depletion was only observed in neurons expressing WT VPS34, whereas neurons expressing non-phosphorylatable mutant VPS34 displayed elevated levels of PI(3)P that remained in the presence of Gabazine. These data that are shown in the **new Fig. EV1I,J** suggest that phosphorylation of VPS34 by Cdk5 indeed is a major contributor to the activity-dependent regulation of neuronal PI(3)P levels.

2. Can the authors confirm the role of Cdk5 in turning down Vps34 activity through reagents other than roscovitine? There are known off-target effects with this drug, so it is important to confirm these results with at least one alternative approach, namely, an independent pharmacological inhibitor/chemotype, a Cdk5 knockdown, or expression of a kinase-dead Cdk5 construct.

Response: We confirmed these results using an alternative Cdk5 inhibitor Dinaciclib (see **new Figure EV1G-H**).

3. The processing of p35 into p25 by calpain 2 seems very striking. Can the authors provide more direct biochemical evidence that this processing induced by PI(3)P depletion increases the kinase activity of Cdk5, by looking either at the kinase activity itself or direct Cdk5 substrates? Additionally, is this processing only observed in cerebellar neurons or can it be shown also in hippocampal/cortical neurons?

Response: We have carried out additional experiments in hippocampal neurons. These experiments confirm that p35 is processed into p25 upon PI(3)P depletion as shown in **new Figure EV3C**.

To our knowledge no assays or probes exist that would enable us to assess the activity of Cdk5 in its natural environment in living neurons. As the pathway described in our study may be local, at least in part, we consider it unlikely that a bulk measurement of Cdk5 activity using available assays or artificial substrates would be suited to address this point.

To nonetheless address the referee's question, we have conducted additional immunoblot analysis using antibodies allegedly suitable to detect phosphorylated variants of Dynamin 1 and Tau. We note that these proteins besides Cdk5 are substrates of multiple other kinases that may act in different compartments. Hence, the bulk levels of Dynamin 1 or Tau phosphorylation may not be indicative of Cdk5 activity. Consistent with these caveats we have not been able to detect consistent changes in the levels of phosphorylated Dynamin 1 or Tau as shown in Figure 1 for referees appended below.

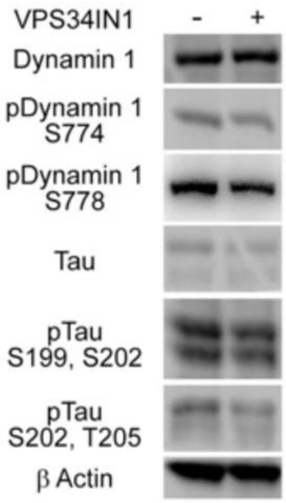

Figure 1 for referees: Levels of phosphorylated dynamin 1 and Tau proteins in cultured cerebellar granule neurons treated with DMSO (0.1%) or VPS34IN1 (10 μM) and analyzed by immunoblotting. The blot shown is representative of three independent experimental replicates with similar results.

We conclude that further studies, possibly using quantitative phosphoproteomic approaches will be required to address the question of Cdk5 targets and local activity. We hope that the referee will agree that such studies go beyond the scope of the present manuscript.

Other comments:

4. Morel et al. is cited as evidence for a role of PI(3)P in non-neuronal cells in the introduction, but this paper, as well as a follow-up study from the same group (Miranda et al. PMID: 29348617), explored the role of Vps34, and thus PI(3)P, in neurons both in vitro (both papers) and in vivo (only Miranda et al.), but not necessarily at synapses. They should probably be cited in the appropriate contexts. Additionally, the Miranda paper demonstrates a key role for neuronal PI(3)P in endolysosomal function as well as autophagy, which should perhaps also be mentioned in this manuscript.

Response: We apologize for the imprecision with reference to these important prior studies. We have amended the text on **p.3** of the revised manuscript accordingly. It now reads as follows:

"Surprisingly little is known about the distribution and dynamics of neuronal endosomes marked by PI(3)P, a lipid of crucial importance for endosome and lysosome function in non-neuronal cells (Balla, 2013; Di Paolo & De Camilli, 2006; Raiborg et al., 2013; Simonsen et al, 1998) and in neurons (Miranda et al, 2018; Morel et al, 2013)."

5. In Figure EV1-C-D, the panels should indicate "Merge" for the merged channels

Response: Done.

6. On page 4, it would be helpful to mention that Cdk5 controls Vps34 via phosphorylation of Thr159, when citing the Furuya et al. 2010 paper.

Response: This is a useful hint that we have gladly followed.

7. Since Cdk5 activity has been heavily implicated in Alzheimer's disease (AD) in part due to its role in tau hyperphosphorylation, is it conceivable that the PI(3)P deficiency observed in AD patients' brains may also impact tau phosphorylation through hyperactivation of Cdk5? It is perhaps worth discussing this point in the Discussion, when thinking about potential implications for neurological diseases.

Response: We thank the referee for the comment. We now discuss this explicitly in our revised manuscript on **p. 11**.

1st Revision - Editorial Decision 23rd Feb 2022

Dear Volker,

Thank you for submitting your revised manuscript to The EMBO Journal. Your revision has now been seen by three referees and their comments are provided below.

Referees #1 and 3 are happy with the revised version. Referee #2 has one remaining issue that can addressed with a better clarification and text changes.

When you submit the revised version, please also take care of the following points

- We need 3-5 keywords

- Conflict of Interest should be Disclosure Statement & Competing Interests see also guide to authors. https://www.embopress.org/page/journal/14602075/authorguide#conflictsofinterest

- please double check that there is a callout to FigEV3E

- Our publisher has also done their pre-publication check on your manuscript. When you log into the manuscript submission system you will see the file "Data Edited Manuscript file". Take a look at the word file and the comments regarding the figure legends and respond to the issues.

- Your source data looks super nice - thank you! There is a Source Data Statistics file that contain the statistics of all the figures. This excel file needs to be split into one file per figure and zipped with the other source data files for that particular figure.

That should be all!

Congratulations on a nice study!

With best wishes

Karin

Karin Dumstrei, PhD
Senior Editor
The EMBO Journal

Guide For Authors: https://www.embopress.org/page/journal/14602075/authorguide

We realize that it is difficult to revise to a specific deadline. In the interest of protecting the conceptual advance provided by the work, we recommend a revision within 3 months (24th May 2022). Please discuss the revision progress ahead of this time with the editor if you require more time to complete the revisions. Use the link below to submit your revision:

https://emboj.msubmit.net/cgi-bin/main.plex

Referee #1:

The authors have satisfactorily addressed the comments by the reviewers. In my opinion, this manuscript is interesting and should now be accepted for publication.

Referee #2:

The authors have addressed a few issues that I had raised. However, I am still concerned about a number of points.
There is a major concern that I cannot find a good fit between their model and current results. First, the authors claim that neuronal activity inhibits PI(3)P synthesis. Second, depletion of PI(3)P decreases neurotransmission. During repetitive neural activities or higher activity (50AP*200AP), PI(3)P synthesis should be suppressed, and consequently neurotransmission should be reduced in the VPS34IN1 or equivalent groups, and even in the CONTROL group if repetitive neural activities continue.

However, both DMSO and VPS34 inhibitor1 set of Fig 2C and Fig 4A-D showed little defect in synaptic transmission. Particularly in Fig.4A and C, the synaptic transmission is less defective in the train of 200 APs than in that of 50 APs. However, I am convinced that endosomal PI(3)P is required for SV endocytosis, since endocytosis seems to be slowed when VIP34IN1 is present. Surface retention is not a good indicator of the concurrent failure of synaptic transmission and SV endocytosis. It would be better if the data were analyzed separately by extracting synaptic transmission and endocytosis. For example, the amount of synaptic transmission and its' accumulation during repetition and the endocytic time constant or t1/2 or similar one would be more appropriate. As many of the results (Fig4, 6, 7, and EV2, 3) are based on these, I am not sure how accurately the effect of PI(3)P can be explained by the proposed model.
At the moment, I am afraid that this study is not sufficient to publish in EMBO J.

Referee #3:

The authors have addressed all my questions and concerns.

Response to the reviewers

We would like to thank the Editor as well as all three referees for their careful reading of our revised manuscript. We were very happy to learn that referees 1 and 3 were happy with our revisions and support publication in *The EMBO J*. Referee 2 has voiced a remaining concern that we have address as detailed below (our response in blue).

Response to reviewer #2:
The authors have addressed a few issues that I had raised. However, I am still concerned about a number of points. There is a major concern that I cannot find a good fit between their model and current results. First, the authors claim that neuronal activity inhibits PI(3)P synthesis. Second, depletion of PI(3)P decreases neurotransmission. During repetitive neural activities or higher activity (50AP*200AP), PI(3)P synthesis should be suppressed, and consequently neurotransmission should be reduced in the VPS34IN1 or equivalent groups, and even in the CONTROL group if repetitive neural activities continue. However, both DMSO and VPS34 inhibitor1 set of Fig 2C and Fig 4A-D showed little defect in synaptic transmission. Particularly in Fig.4A and C, the synaptic transmission is less defective in the train of 200 APs than in that of 50 APs. However, I am convinced that endosomal PI(3)P is required for SV endocytosis, since endocytosis seems to be slowed when VIP34IN1 is present. Surface retention is not a good indicator of the concurrent failure of synaptic transmission and SV endocytosis. It would be better if the data were analyzed separately by extracting synaptic transmission and endocytosis. For example, the amount of synaptic transmission and its' accumulation during repetition and the endocytic time constant or t1/2 or similar one would be more appropriate. As many of the results (Fig4, 6, 7, and EV2, 3) are based on these, I am not sure how accurately the effect of PI(3)P can be explained by the proposed model.

Response: We are sorry to hear that reviewer 2 has a remaining major concern pertaining to the data presented in Figs. 2 and 4 and their relationship to the reported role of PI(3)P in regulating neurotransmission. The issue the referee alludes to is related to the fact that with AP stimulation trains as in the experiments shown in Figs2C and 4A-D, which result in profound presynaptic calcium elevation, we were unable to see an overt defect in synaptic vesicle exocytosis monitored by SynaptopHluorin.

This is neither unexpected nor does this conflict with our electrophysiological data regarding exocytic neurotransmission shown in Figs. 2A, 6 and 7. Let me explain: In our electrophysiological recordings we monitor the exocytic response to *single depolarizing stimuli*. Under conditions of PI(3)P depletion we observe a depression of exocytic responses owed to a reduction in presynaptic release probability (Fig. 2A,B). We do not observe a blockade in neurotransmission. The specificity of these phenotypes and their causal relationship to PI(3)P depletion is demonstrated by the rescue experiments shown in Figs. 6 and 7, as acknowledged by all referees. It is well established that high level presynaptic calcium accumulation that is typically induced by *AP train stimulation* (see the seminal work of Katz and Miledi, *J. Physiol.* 1968), e.g. repeated AP trains as in Figs. 2C and 4A-D, can override such defects in release probability. The two datasets referred to by the referee as thus non-contradictory.

That said, the referee seems to have overlooked the new data displayed in **Extended View Figure EV4A-B**, in which we have used a SynaptopHluorin-based assay to monitor SV exocytosis in response to 10 AP stimulation following trains of 200 AP stimulations that cause a reduction in PI(3)P levels. These data reveal a significant downregulation of the exocytic responses following train stimulation even when using the comparably insensitive SynaptopHluorin assay as a readout. Importantly, the exocytic phenotype is rescued by the

Cdk5 inhibitor Roscovitine, demonstrating that reduced synaptic vesicle exocytosis is a consequence of Cdk5 hyperactivation downstream of PI(3)P depletion. These data thus confirm our results from electrophysiological analyses shown in Figs. 2A,B,6, and 7.

Aside from these considerations, we would like to emphasize that in our manuscript, we do not make specific predictions as to whether the reduced release probability demonstrated in Figs. 2A,B, 6, and 7 and in EV4A,B is a direct consequence of impaired SV endocytosis. In fact, it is conceivable that the PI(3)P- and calpain-dependent regulatory pathway described in our study controls presynaptic neurotransmitter release and SV endocytosis via *distinct* effector proteins downstream of Cdk5, i.e. exocytic depression and defective endocytosis are semi-independent phenotypes as discussed in the revised version of our manuscript on p.11.

To clarify these issues and to address the referee's concern, we have now amended the manuscript text as follows (new wording underlined):

Results

• p.5 - with respect to the electrophysiological analysis and the relationship between reduced presynaptic neurotransmission and vesicle cycling we now conclude:

"These data indicate that repression of VPS34-mediated PI(3)P synthesis reduces basal excitatory neurotransmission in response to depolarizing stimuli, likely via alterations in presynaptic release probability. As VPS34 operates mainly on endosomes and PI(3)P is absent from the plasma membrane (Balla, 2013; Di Paolo & De Camilli, 2006; Gaullier *et al.*, 1998; Ketel *et al.*, 2016; Morel *et al.*, 2013; Raiborg *et al.*, 2013), we speculated that reduced basal neurotransmission in PI(3)P-depleted neurons might be accompanied or partially caused by impaired SV endocytosis and/ or recycling, which may involve endosome-like intermediates (Chanaday *et al.*, 2019; Jahne *et al.*, 2015; Rizzoli, 2014; Watanabe *et al.*, 2014)."

• p.5 - with respect to the data shown in Fig. 2C, we have added the following sentence:

"Under these conditions of repeated AP train stimulation (see also Figure 4A-D) no overt defect in SV exocytosis was observed, suggesting that AP train-induced presynaptic calcium elevation can override a reduction in release probability caused by depletion of PI(3)P."

• p. 6/ top - we have amended our conclusion on the preceding paragraph to make clear that reduced release probability and impaired SV endocytosis may not necessarily be causally related (although they could be):

"These data show that PI(3)P depletion perturbs SV endocytosis and reduces presynaptic release probability."

Discussion

• Finally, we have added the following sentence (underlined) to the discussion on p.11:

"Second, given the multiple roles of Cdk5 in presynaptic neurotransmission and SV cycling, it is possible that hyperactivation of Cdk5 impinges not only on SV endocytosis (Armbruster *et al.*, 2013; Tan *et al.*, 2003) but may involve additional target proteins such as voltage-gated calcium channels (Kim & Ryan, 2010), crucial factors for the regulation of presynaptic release probability. Consistently, we observe that AP train-induced presynaptic calcium elevation can override the reduction in release probability caused by depletion of PI(3)P but not the defects in SV endocytosis (see Figs. 2C and 4A-D)."

2nd Revision - Editorial Decision 1st Mar 2022

Dear Volker,

Thank you for submitting your revised manuscript to The EMBO Journal. I have now had the chance to take a look at everything and all looks good.

I am therefore very pleased to accept the manuscript.

Congratulations on a nice study!

With best wishes

Karin

Karin Dumstrei, PhD
Senior Editor
The EMBO Journal

Please note that it is EMBO Journal policy for the transcript of the editorial process (containing referee reports and your response letter) to be published as an online supplement to each paper. If you do NOT want this, you will need to inform the Editorial Office via email immediately. More information is available here: https://www.embopress.org/page/journal/14602075/authorguide#transparentprocess

Your manuscript will be processed for publication in the journal by EMBO Press. Manuscripts in the PDF and electronic editions of The EMBO Journal will be copy edited, and you will be provided with page proofs prior to publication. Please note that supplementary information is not included in the proofs.

Please note that you will be contacted by Wiley Author Services to complete licensing and payment information. The required 'Page Charges Authorization Form' is available here: https://www.embopress.org/pb-assets/embo-site/tej_apc.pdf

Should you be planning a Press Release on your article, please get in contact with embojournal@wiley.com as early as possible, in order to coordinate publication and release dates.

If you have any questions, please do not hesitate to call or email the Editorial Office. Thank you for your contribution to The EMBO Journal.

** Click here to be directed to your login page: https://emboj.msubmit.net

EMBO Press Author Checklist

Corresponding Author Name: Tolga Soykan, Volker Haucke	
Journal Submitted to: The EMBO Journal	
Manuscript Number: EMBOJ-2021-109352	

USEFUL LINKS FOR COMPLETING THIS FORM
The EMBO Journal - Author Guidelines
EMBO Reports - Author Guidelines
Molecular Systems Biology - Author Guidelines
EMBO Molecular Medicine - Author Guidelines

Reporting Checklist for Life Science Articles (updated January 2022)
This checklist is adapted from Materials Design Analysis Reporting (MDAR) Checklist for Authors. MDAR establishes a minimum set of requirements in transparent reporting in the life sciences (see Statement of Task: 10.31222/osf.io/9sm4x). Please follow the journal's guidelines in preparing your manuscript.
Please note that a copy of this checklist will be published alongside your article.

Abridged guidelines for figures
1. Data
The data shown in figures should satisfy the following conditions:
- the data were obtained and processed according to the field's best practice and are presented to reflect the results of the experiments in an accurate and unbiased manner.
- ideally, figure panels should include only measurements that are directly comparable to each other and obtained with the same assay.
- plots include clearly labeled error bars for independent experiments and sample sizes. Unless justified, error bars should not be shown for technical replicates.
- if n<5, the individual data points from each experiment should be plotted. Any statistical test employed should be justified.
- Source Data should be included to report the data underlying figures according to the guidelines set out in the authorship guidelines on Data Presentation.

2. Captions
Each figure caption should contain the following information, for each panel where they are relevant:
- a specification of the experimental system investigated (eg cell line, species name).
- the assay(s) and method(s) used to carry out the reported observations and measurements.
- an explicit mention of the biological and chemical entity(ies) that are being measured.
- an explicit mention of the biological and chemical entity(ies) that are altered/varied/perturbed in a controlled manner.
- the exact sample size (n) for each experimental group/condition, given as a number, not a range;
- a description of the sample collection allowing the reader to understand whether the samples represent technical or biological replicates (including how many animals, litters, cultures, etc.).
- a statement of how many times the experiment shown was independently replicated in the laboratory.
- definitions of statistical methods and measures:
  - common tests, such as t-test (please specify whether paired vs. unpaired), simple χ2 tests, Wilcoxon and Mann-Whitney tests, can be unambiguously identified by name only, but more complex techniques should be described in the methods section;
  - are tests one-sided or two-sided?
  - are there adjustments for multiple comparisons?
  - exact statistical test results, e.g., P values = x but not P values < x;
  - definition of 'center values' as median or average;
  - definition of error bars as s.d. or s.e.m.

Please complete ALL of the questions below.
Select "Not Applicable" only when the requested information is not relevant for your study.

Materials

**Newly Created Materials**	**Information included in the manuscript?**	**In which section is the information available?** (Reagents and Tools Table, Materials and Methods, Figures, Data Availability Section)
New materials and reagents need to be available; do any restrictions apply?	Yes	Data availability

**Antibodies**	**Information included in the manuscript?**	**In which section is the information available?** (Reagents and Tools Table, Materials and Methods, Figures, Data Availability Section)
For **antibodies** provide the following information:<br>- Commercial antibodies: RRID (if possible) or supplier name, catalogue number and or/clone number<br>- Non-commercial: RRID or citation	Yes	Reagents and Tools Table

**DNA and RNA sequences**	**Information included in the manuscript?**	**In which section is the information available?** (Reagents and Tools Table, Materials and Methods, Figures, Data Availability Section)
**Short novel DNA or RNA including primers, probes**: provide the sequences.	Not Applicable	

**Cell materials**	**Information included in the manuscript?**	**In which section is the information available?** (Reagents and Tools Table, Materials and Methods, Figures, Data Availability Section)
**Cell lines:** Provide species information, strain. Provide accession number in repository **OR** supplier name, catalog number, clone number, and/**OR** RRID.	Not Applicable	
**Primary cultures:** Provide species, strain, sex of origin, genetic modification status.	Yes	Materials and Methods (Preparation of primary hippocampal and cerebellar neuron cultures and transfection)
Report if the cell lines were recently **authenticated** (e.g., by STR profiling) and tested for mycoplasma contamination.	Not Applicable	

**Experimental animals**	**Information included in the manuscript?**	**In which section is the information available?** (Reagents and Tools Table, Materials and Methods, Figures, Data Availability Section)
**Laboratory animals or Model organisms:** Provide species, strain, sex, age, genetic modification status. Provide accession number in repository **OR** supplier name, catalog number, clone number, **OR** RRID.	Yes	Materials and Methods (Animals for neuron cultures and acute slice preparations)
**Animal observed in or captured from the field:** Provide species, sex, and age where possible.	Not Applicable	
Please detail **housing and husbandry conditions**.	Yes	Materials and Methods (Animals for neuron cultures and acute slice preparations)

**Plants and microbes**	**Information included in the manuscript?**	**In which section is the information available?** (Reagents and Tools Table, Materials and Methods, Figures, Data Availability Section)
**Plants:** provide species and strain, ecotype and cultivar where relevant, unique accession number if available, and source (including location for collected wild specimens).	Not Applicable	
**Microbes:** provide species and strain, unique accession number if available, and source.	Not Applicable	

**Human research participants**	**Information included in the manuscript?**	**In which section is the information available?** (Reagents and Tools Table, Materials and Methods, Figures, Data Availability Section)
If collected and within the bounds of privacy constraints report on age, sex and gender or ethnicity for all study participants.	Not Applicable	

**Core facilities**	**Information included in the manuscript?**	**In which section is the information available?** (Reagents and Tools Table, Materials and Methods, Figures, Data Availability Section)
If your work benefited from core facilities, was their service mentioned in the acknowledgments section?	Not Applicable	

Design

**Study protocol**	**Information included in the manuscript?**	**In which section is the information available?** (Reagents and Tools Table, Materials and Methods, Figures, Data Availability Section)

If study protocol has been **pre-registered, provide DOI in the manuscript**. For clinical trials, provide the trial registration number **OR** cite DOI.	Not Applicable	
Report the **clinical trial registration number** (at ClinicalTrials.gov or equivalent), where applicable.	Not Applicable	

Laboratory protocol	Information included in the manuscript?	In which section is the information available? (Reagents and Tools Table, Materials and Methods, Figures, Data Availability Section)
Provide DOI OR other citation details if **external detailed step-by-step protocols** are available.	Not Applicable	

Experimental study design and statistics	Information included in the manuscript?	In which section is the information available? (Reagents and Tools Table, Materials and Methods, Figures, Data Availability Section)
Include a statement about **sample size** estimate even if no statistical methods were used.	Yes	Materials and Methods (Statistics and Reproducibility)
Were any steps taken to minimize the effects of subjective bias when allocating animals/samples to treatment (e.g. **randomization procedure**)? If yes, have they been described?	Not Applicable	
Include a statement about **blinding** even if no blinding was done.	Yes	Materials and Methods (Statistics and Reproducibility)
Describe **inclusion/exclusion criteria** if samples or animals were excluded from the analysis. Were the criteria pre-established?<br><br>If sample or data points were omitted from analysis, report if this was due to attrition or intentional exclusion and provide justification.	Not Applicable	
For every figure, are **statistical tests** justified as appropriate? Do the data meet the assumptions of the tests (e.g., normal distribution)? Describe any methods used to assess it. Is there an estimate of variation within each group of data? Is the variance similar between the groups that are being statistically compared?	Yes	Materials and Methods (Statistics and Reproducibility)

Sample definition and in-laboratory replication	Information included in the manuscript?	In which section is the information available? (Reagents and Tools Table, Materials and Methods, Figures, Data Availability Section)
In the figure legends: state number of times the experiment was **replicated** in laboratory.	Yes	Figure Legends
In the figure legends: define whether data describe **technical or biological replicates**.	Yes	Figure Legends, Materials and Methods (Statistics and Reproducibility)

Ethics

Ethics	Information included in the manuscript?	In which section is the information available? (Reagents and Tools Table, Materials and Methods, Figures, Data Availability Section)
Studies involving **human participants**: State details of **authority granting ethics approval** (IRB or equivalent committee(s), provide reference number for approval.	Not Applicable	
Studies involving **human participants**: Include a statement confirming that **informed consent** was obtained from all subjects and that the experiments conformed to the principles set out in the WMA Declaration of Helsinki and the Department of Health and Human Services Belmont Report.	Not Applicable	
Studies involving **human participants:** For publication of **patient photos**, include a statement confirming that consent to publish was obtained.	Not Applicable	
Studies involving experimental **animals**: State details of **authority granting ethics approva**l (IRB or equivalent committee(s), provide reference number for approval. Include a statement of compliance with ethical regulations.	Yes	Materials and Methods (Animals for neuron cultures and acute slice preparations)
Studies involving **specimen and field samples:** State if relevant **permits** obtained, provide details of authority approving study; if none were required, explain why.	Not Applicable	

Dual Use Research of Concern (DURC)	Information included in the manuscript?	In which section is the information available? (Reagents and Tools Table, Materials and Methods, Figures, Data Availability Section)
Could your study fall under dual use research restrictions? Please check biosecurity documents and list of **select agents and toxins** (CDC): https://www.selectagents.gov/sat/list.htm	Not Applicable	
If you used a select agent, is the security level of the lab appropriate and reported in the manuscript?	Not Applicable	
If a study is subject to dual use research of concern regulations, is the name of the **authority granting approval and reference number** for the regulatory approval provided in the manuscript?	Not Applicable	

Reporting

The MDAR framework recommends adoption of discipline-specific guidelines, established and endorsed through community initiatives. Journals have their own policy about requiring specific guidelines and recommendations to complement MDAR.

Adherence to community standards	Information included in the manuscript?	In which section is the information available? (Reagents and Tools Table, Materials and Methods, Figures, Data Availability Section)
State if relevant guidelines or checklists (e.g., **ICMJE, MIBBI, ARRIVE, PRISMA**) have been followed or provided.	Not Applicable	
For **tumor marker prognostic studies,** we recommend that you follow the **REMARK** reporting guidelines (see link list at top right). See author guidelines, under 'Reporting Guidelines'. Please confirm you have followed these guidelines.	Not Applicable	
For **phase II and III randomized controlled trials**, please refer to the **CONSORT** flow diagram (see link list at top right) and submit the CONSORT checklist (see link list at top right) with your submission. See author guidelines, under 'Reporting Guidelines'. Please confirm you have submitted this list.	Not Applicable	

Data Availability

Data availability	Information included in the manuscript?	In which section is the information available? (Reagents and Tools Table, Materials and Methods, Figures, Data Availability Section)
Have **primary datasets** been deposited according to the journal's guidelines (see 'Data Deposition' section) and the respective accession numbers provided in the Data Availability Section?	Not Applicable	
Were **human clinical and genomic datasets** deposited in a public access-controlled repository in accordance to ethical obligations to the patients and to the applicable consent agreement?	Not Applicable	
Are **computational models** that are central and integral to a study available without restrictions in a machine-readable form? Were the relevant accession numbers or links provided?	Not Applicable	
If publicly available data were reused, provide the respective **data citations in the reference list**.	Not Applicable	
