# Supplementary material for: Endosomal phosphatidylinositol 3‐phosphate controls synaptic vesicle cycling and neurotransmission
Source: EMBO J. 2022 Mar 22;41(9):e109352. doi: 10.15252/embj.2021109352 (PMC9058544; doi:10.15252/embj.2021109352)
Supplement: Supplementary file 3 — Source Data for Expanded View and Appendix [file EMBJ-41-e109352-s010.zip › Figure_EV5_Source_Data_Statistics.zip!/Figure EV5_Source Data+Statistics/Figure EV5F/Picrotoxin.pdf]

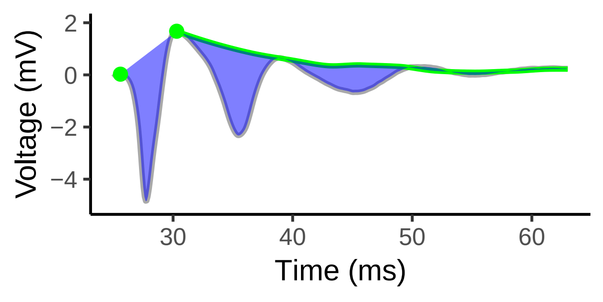

Voltage (mV)
2
0
−2
−4
30
40
50
60
Time (ms)
